# Synthesis and multifaceted exploration of dibenzoxepinones: *in vitro* antimicrobial and ct-DNA binding, DFT/TD-DFT, molecular docking and simulation studies†

**DOI:** 10.1039/d5ra01068c

**Published:** 2025-05-30

**Authors:** Shilpa Yadav, Mansi, Priyanshu, Pratibha Chanana, Pankaj Khanna, Asmita Singh, Leena Khanna

**Affiliations:** a University School of Basic & Applied Sciences, Guru Gobind Singh Indraprastha University Dwarka New Delhi-110078 India leenakhanna@ipu.ac.in; b University School of Chemical Technology, Guru Gobind Singh Indraprastha University Dwarka New Delhi-110078 India; c Synthesis & In-Silico Drug Design Laboratory, Department of Chemistry, Acharya Narendra Dev College, University of Delhi Kalkaji New Delhi-110019 India

## Abstract

In the present study, ten novel derivatives of dibenzoxepine-11-one, have been synthesized and thoroughly characterized using spectroscopic techniques, including ^1^H and ^13^C NMR, FTIR, and HRMS. Density Functional Theory (DFT) calculations were carried out to analyze the geometrical structure and vibrational modes, enabling the identification of the most stable conformation of the lead compound. Time-Dependent DFT (TD-DFT) was employed to investigate the electronic transitions within the UV-Vis spectrum. Molecular docking studies were performed for all derivatives using various target proteins with PDB IDs:IKZN (for *E. coli*), 1IYL (for *C. albicans*), and the DNA dodecamer structure (PDB ID: 1BNA). The results revealed favorable binding interactions across all targets. Additionally, molecular dynamics (MD) simulations were conducted for 50 ns using the most promising compound, 7a, confirming the stability of its binding conformation. From *in vitro* studies, antibacterial activity was assessed for all the synthesized derivatives against Gram-positive strains (*B. subtilis*, *L. rhamnosus*) and a Gram-negative strain (*E. coli*). Compounds 7a, 7b, 7c, 7d, and 7f exhibited strong antibacterial efficacy, with minimum inhibitory concentration (MIC) values of 8 μg ml^−1^ for *E. coli* and *L. rhamnosus* and 16 μg ml^−1^ for *B. subtilis* bacterial strains. Additionally, compound 7a exhibited good antifungal activity, with a maximum zone of inhibition of 18 mm against the fungal strain *C. albicans*. Further UV-Vis absorption and fluorescence quenching studies, conducted for compounds 7a, 7b, and 7e with calf thymus DNA (ct-DNA), suggested a groove-binding interaction. Compound 7a demonstrated the strongest binding affinity, with a binding constant (*K*_b_) of 3.61 × 10^5^ M^−1^ and a Gibbs free energy change (Δ*G*) of −31.70 kJ mol^−1^. Therefore, these novel dibenzoxepine-11-ones derivatives are multifaceted in their action as potential antimicrobial and DNA-binding agents, and will be useful in developing new therapeutics.

## Abstract 2

In the present study, ten novel derivatives of dibenzoxepine-11-one, have been synthesized and thoroughly characterized using spectroscopic techniques, including ^1^H and ^13^C NMR, FTIR, and HRMS.

## Graphical abstract

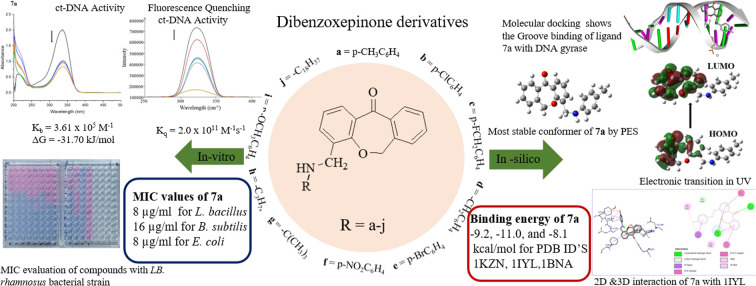

## 1. Introduction

Dibenzoxepinone is a tricyclic heterocycle featuring two fused benzene rings and a seven-membered oxepine ring. This chemical structure serves as the foundation for various pharmacological applications such as antibacterial, antifungal, antitumor, antihistamine, antidepressant, and anti-inflammatory.^1–3^ A prominent example of a dibenzoxepinone derivative is doxepin, which treats depression, anxiety, and chronic skin conditions such as urticaria.^4,5^ Its other derivatives, such as Oxepinoquinones and Napthodibenzoxepine, disrupt bacterial membrane integrity, leading to bacterial cell death and also preventing bacterial DNA replication. Halogenated dibenzoxepinone inhibits fungal membrane integrity and reduces infection persistence.^6^ These compounds often disrupt bacterial cell walls, inhibit DNA replication, or target specific bacterial enzymes. Modifying the core dibenzoxepinone framework can improve antibacterial activity against various bacterial strains, including both Gram-negative and Gram-positive bacteria.^7^ Furthermore, additional derivatives of dibenzoxepinone are under investigation as potential anticancer agents, as their structure can be altered to interact with key processes in cancer cells. They have the potential to block cancer cell growth by interfering with DNA, inhibiting crucial enzymes, or triggering cell death.^8,9^Thus, motivated by the diverse importance of dibenzoxepinone and as active researchers of heterocyclic compounds,^10–14^ we propose synthesizing novel derivatives of dibenzoxepin-11-one. The methodology involves synthesizing dibenzoxepin-11-one, followed by introducing various amines to one of its aromatic rings *via* a plausible route to assess their biological applications. Ten novel compounds were prepared and characterized using various spectroscopic techniques. DFT studies provided a potential energy scan for different conformations of the best compound. UV and FTIR calculations were also performed to observe the electronic transitions and vibrations of the compound and compare the data with the experimental results.^15,16^ Molecular docking was conducted with potential targets of antibacterial, antifungal, and ct-DNA using PDB IDs: 1KZN, 1IYL, and 1BNA through AutoDock 4.2.^17–19^ This aimed to determine the binding energy and uncover how proteins interact with ligands, along with the types of bonds formed between proteins and ligands. An MD simulation study was carried out for 50 ns to evaluate the stability of the protein-ligand complex in a biological system.^20,21^ Antibacterial and antifungal *in vitro* studies were performed to inhibit the growth of bacterial strains (*E. coli*, *L. rhamnosus* and *B. subtilis*) and a fungal strain (*C. albicans*) by MIC and Zone of inhibition. Additionally, UV-vis absorption and fluorescence quenching *in vitro* experiments were conducted to evaluate the DNA binding abilities of the dibenzoxepinone derivatives.^22,23^

## 2. Experimental techniques

### 2.1 Materials and methods

Reagents used in the synthesis of products were purchased from Sigma Aldrich. Positive control drugs Ampicillin and Fluconazole were purchased from SRL. Solvents were purchased by Merck and SRL. TLC plates of silica gel mesh 60F_254_ (Merck) were used as a preliminary test to check the progress of the reaction by the spots located in UV and Iodine vapors. Jeol Spectrometer of 400 MHz was used for ^1^H and ^13^C (TMS as a reference). FTIR spectra were recorded by PerkinElmer Spectrum BX spectrophotometer. HRMS was recorded by ESI-MS Thermo LTQ Orbitrap XL in methanol. Absorbance for the ct-DNA binding activity and UV Fluorescence quenching study property was taken by UV-1900 Shimadzu UV VIS Spectrophotometer, Double Beam.

### 2.2 Synthesis

Dibenzoxepinone derivatives were synthesized in four steps. First step involved the formation of aryl benzyl ether (3) *via* Williamson ether synthesis. The second step is the cyclodehydration or intramolecular Friedel–Crafts acylation reaction using lewis acid FeCl_3_ and DCME to form dibenzoxepin-11-one (4). Third step is bromination of methyl group present in the aromatic ring of dibenzoxepin-11-one *via* Wohl–Zeigler bromination reaction *i.e.* allylic bromination using with *N*-bromosuccinimide. Fourth step is the nucleophilic displacement of bromine group by various aliphatic and aromatic amines to get the desired novel dibenzoxepinone derivatives (7a–j) as the product.^24–27^

#### 2.2.1 Synthesis for 2-((*o*-tolyl oxy)methyl)benzoic acid (3)

1 mmol of *o*-cresol (2) and 1.5 mmol of KH was added to the 15 ml of dry DMF under nitrogen, stirred the reaction mixture for 15–20 min. Then, 1 mmol of phthalide (1) solution in toluene was added, and the reaction mixture was refluxed for 24 h. Ice-cooled water was added to quench the reaction and then acidified with conc. HCl and residue obtained was filtered.^28^ Color-pale yellow, yield 75%, mp 116–118 °C; ^1^H-NMR (400 MHz, CDCl_3_): *δ* 2.34 (s, 1H, C**H**_**3**_), 5.52 (s, 2H, O–C**H**_**2**_), 6.87 (m, 2H, ArH), 7.14 (m, 2H, Ar–H), 7.40 (t, 1H, *J* = 8Hz, ArH) 7.62 (t, 1H, *J* = 8Hz, ArH), 7.84 (t, 1H, *J* = 8Hz), 8.13 (d, 1H, *J* = 8Hz). HRMS calcd [M + H]^+^ for C_15_H_14_O_3_: 243.0979, found: 243.1024.

#### 2.2.2 Synthesis for 4-methyl-6*H*-dibenzo[*b*,*e*]oxepine-11-one (4)

**Scheme 1:**
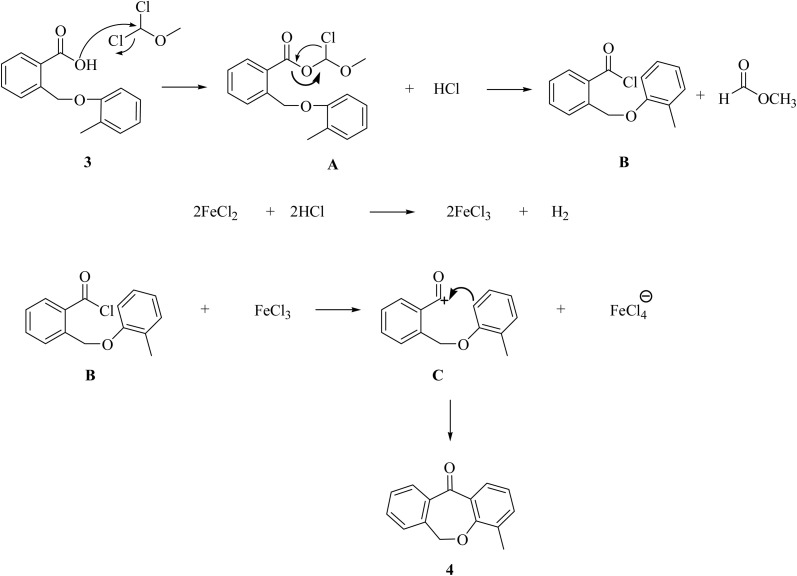
Plausible mechanism of intramolecular cyclization for step (ii).

1 mmol of synthesized product (3), 1.5 mmol of anhydrous FeCl_2_ or anhydrous CeCl_3_, 1 mmol of DCME (dichloromethyl methyl ether) were added in DCM under nitrogen and stirred for 4 h at room temperature. It was mentioned in the literature that by using different Lewis acid catalysts like SnCl_4_, FeCl_3_, AlCl_3_, and FeBr_3_ for this intramolecular cyclization, no or less yield was obtained but the maximum yield was found in the case of FeCl_2_.^28^ However, when we have taken a new Lewis acid catalyst CeCl_3_,^29^ and observed that FeCl_2_ formed the product in 90% yield while CeCl_3_ formed the product in 65% only. Color – light yellow, yield 70%, mp 132–133 °C; ^1^H-NMR (400 MHz, CDCl_3_): *δ* 2.26 (s, 1H, C**H**_**3**_), 5.22 (s, 2H, O–C**H**_**2**_), 7.00 (t, 1H, *J* = 8Hz, ArH), 7.36 (t, 1H, *J* = 8Hz, Ar–H), 7.45 (m, 2H, ArH). 7.54 (t, 1H, *J* = 8Hz, ArH), 7.86 (d, 1H, *J* = 8Hz), 8.06 (d, 1H, *J* = 8Hz). ^13^C-NMR: *δ* 16.81 (**C**H_3_), 73.62 (O–**C**H_2_), 121.58, 127.62, 129.29, 129.42, 129.52, 129.86, 132.66, 135.85, 136.44 (aromatic region). HRMS calcd [M + H]^+^ for C_15_H_12_O_2_: 225.0869, found: 225.1024 (Scheme 1).

For this step we have proposed a mechanism to highlight the importance of Lewis acid FeCl_2_ over FeCl_3_ in this cyclization. This is an intramolecular cyclization *via* the Friedel–Crafts acylation mechanism in which a molecule containing both an aromatic ring and an acyl group forms cyclic ketone. The reaction begins with the conversion of the carboxylic group into an acid chloride group in the presence of DCME (dimethyl methyl ether) which is a well-known reagent for this conversion to form intermediate acid chloride (B) *via*A.^30^ In this process HCl is liberated which reacts with FeCl_2_ to generate FeCl_3_*in situ*. The FeCl_3_ further acts as a lewis acid for B and helps to generate acylium ion (C).^31^ The acylium group then attacks the aromatic ring and intramolecular acylation occurs within the same molecule and creating a new cyclic structure as 4-methyl-dibenzoxepin-11-one (4).

#### 2.2.3 Synthesis for 4-bromomethyl-6*H*-dibenzo[*b*,*e*]oxepine-11-one (5)

1 mmol of (4), 1.5 mmol of NBS (*N*-bromosuccinimide), 2 mmol of DBP (dibenzoyl peroxide) were mixed in CCl_4_, after 10–15 min. The red-colored solution was observed, then again stirred the reaction mixture until the red color disappeared. The solution was kept stirred for 2 h and then added water, extracted by ethyl acetate, and evaporated the organic layer to get the product.^32^ Color – yellow, yield 95%, mp 103–105 °C; ^1^H-NMR (400 MHz, CDCl_3_): *δ* 3.49 (s, 2H, C**H**_**2**_–Br) 5.20 (s, 2H, O–C**H**_**2**_), 7.07 (m, ArH), 7.44 (m, Ar–H). ^13^C-NMR: *δ* 29.81 (**C**H_2_ Br), 74.20 (O–**C**H_2_), 120.83, 122.35, 128.10, 129.60, 129.78, 132.26, 133.03, 135.60, 135.66, 161.42 (carbonyl carbon). HRMS calcd [M + H]^+^ for C_15_H_11_BrO_2_: 301.99, found: 303.0006.

#### 2.2.4 Synthesis of (7a–j)

1 mmol of different amines (6a–j), 1.5 mmol of KH was taken in 15 ml dry DMF under nitrogen after stirring of 15–20 min, 1 mmol of 5 was added to it and again stirred for 22 h at ambient temperature. The reaction was quenched with ice-cooled water and the product was extracted from ethyl acetate and the solvent was evaporated to get the desired product.^27^ All these compounds were fully characterized by various spectroscopic techniques and the spectrums were provided in a ESI file.†

#### 2.2.5 7-(*p*-Toylyamino-methyl)-6*H*-dibenzo[*b*,*e*]oxepin-11-one (7a)

Color – brown, yield 94%, mp 324–327 °C; ^1^H-NMR (400 MHz, CDCl_3_): *δ* 2.13 (s, 3H, C**H**_**3**_) 4.45 (s, 1H, N**H**), 4.95 (s, 2H, N–C**H**_**2**_), 5.25 (s, 2H, O–C**H**_**2**_), 6.93 (t, 2H, *J* = 8Hz, ArH), 7.10 (m, 3H, Ar–H), 7.41 (d, 2H, *J* = 8Hz, Ar–H), 7.53 (d, 2H, *J* = 8Hz, Ar–H), 7.70 (m, 3H–Ar–H). ^13^C-NMR: *δ* 16.84 (**C**H_3_), 50.80 (N–**C**H_2_), 73.62 (O–**C**H_2_), 121.58, 127.62, 129.29, 129.42, 129.52, 129.86, 132.66, 136.44, 142.68, 145.87, 146.45 (aromatic region). HRMS calcd [M + H]^+^ for C_22_H_19_NO_2_: 330.14, found: 330.1520.

#### 2.2.6 7-[(*p*-Chloro-phenylamino)-methyl]-6*H*-dibenzo[*b*,*e*]oxepin-11-one (7b)

Color – light brown, yield 97%, mp 356–358 °C; ^1^H-NMR (400 MHz, CDCl_3_): *δ* 4.12 (s, 1H, N**H**) 4.98 (s, 2H, N–C**H**_**2**_), 5.24 (s, 2H, O–C**H**_**2**_), 6.87 (t, 2H, *J* = 8Hz, ArH), 7.18 (m, 3H, Ar–H), 7.40 (t, 2H, *J* = 8Hz, ArH), 7.62 (d, 2H, *J* = 8Hz, ArH), 7.84 (d, 1H, *J* = 8Hz, Ar–H), 8.14 (m, 1H, Ar–H). ^13^C-NMR: *δ* 55.41 (N–**C**H_2_), 74.06 (O–**C**H_2_), 114.44, 114.68, 114.93, 117.35, 124.03, 127.29, 128.27, 128.68, 129.93, 130.07, 130.29, 133.16, 138.06, 153.45, 170.80 (carbonyl carbon). HRMS calcd [M + H]^+^ for C_21_H_16_ClNO_2_: 351.09, found: 351.0979.

#### 2.2.7 7-[(*p*-Fluoro-phenylamino-methyl)]-6*H*-dibenzo[*b*,*e*]oxepin-11-one (7c)

Color – light brown, yield 95%, mp 275–276 °C; ^1^H-NMR (400 MHz, CDCl_3_): *δ* 4.01 (s, 1H, N**H**), 4.76 (s, 2H, N–C**H**_**2**_), 4.95 (s, 2H, N–C**H**_**2**_), 5.21 (s, 2H, O–C**H**_**2**_), 6.87 (t, 2H, *J* = 8Hz, Ar–H), 7.18 (m, 2H, ArH), 7.40 (t, 2H, *J* = 8Hz, Ar–H), 7.62 (t, 2H, *J* = 8Hz, Ar–H), 7.84 (d, 2H, Ar–H), 8.14 (d, 1H, *J* = 8Hz, Ar–H). ^13^C-NMR: *δ* 52.80 (N–**C**H_2_), 54.99 (N–**C**H_2_), 72.94(O–**C**H_2_), 119.99, 121.86, 122.87, 124.01, 126.55, 129.88, 131.00, 132.86, 133.87, 142.99, 147.45, 170.45 (carbonyl carbon). HRMS calcd [M + H]^+^ for C_22_H_18_FNO_2_: 348.13, found: 348.1407.

#### 2.2.8 7-[(Benzylamino-methyl)]-6*H*-dibenzo[*b*,*e*]oxepin-11-one (7d)

Color – brown, yield 97%, mp 249–250 °C; ^1^H-NMR (400 MHz, CDCl_3_): *δ* 4.46 (s, 1H, N**H**), 4.81 (s, 2H, N–C**H**_**2**_), 5.06 (s, 2H, N–C**H**_**2**_), 5.18 (s, 2H, O–C**H**_**2**_), 6.92 (t, 1H, *J* = 8Hz, Ar–H), 7.09 (m, 2H, ArH), 7.13 (m, 3H, ArH), 7.82 (d, 2H, *J* = 8Hz, Ar–H), 8.22 (d, 1H, *J* = 8Hz, Ar–H), 8.34 (d, 2H, *J* = 8Hz, Ar–H). ^13^C-NMR: *δ* 46.85 (N–**C**H_2_), 52.49 (N–**C**H_2_), 75.47 (O–**C**H_2_), 128.46, 128.85, 129.29, 129.78, 130.17, 133.74, 137.28, 137.77, 138.16, 141.74, 142.28, 171.81 (carbonyl carbon). HRMS calcd [M + H]^+^ for C_22_H_19_NO_2_: 330.09, found: 330.1494.

#### 2.2.9 7-[(*p*-Bromo-phenylamino-methyl)]-6*H*-dibenzo[*b*,*e*]oxepin-11-one (7e)

Color – brown, yield 95%, mp 360–363 °C; ^1^H-NMR (400 MHz, CDCl_3_): *δ* 4.73 (s, 1H, N**H**), 4.99 (s, 2H, N–C**H**_**2**_), 5.34 (s, 2H, O–C**H**_**2**_), 6.89 (m, 2H, ArH), 7.39 (t, 1H, *J* = 8Hz, Ar–H), 7.61 (t, 1H, *J* = 8Hz, Ar–H), 7.85 (d, 2H, *J* = 8Hz, Ar–H), 7.93 (d, 1H, *J* = 8Hz, Ar–H), 8.15 (d, 2H, *J* = 8Hz, Ar–H), 8.23 (m, 2H, Ar–H). ^13^C-NMR: *δ* 52.64 (N–**C**H_2_), 73.62 (O–**C**H_2_), 121.58, 127.62, 129.29, 129.42, 129.52, 129.86, 132.66, 135.85, 136.44, 141.57, 147.62, 149.29, 149.42, 149.52, 149.85, 152.66, 155.85, 156.44 (aromatic region). HRMS calcd [M + H]^+^ for C_21_H_16_BrNO_2_: 394.04, found: 394.0433.

#### 2.2.10 7-[(*p*-Nitro-phenylamino-methyl)]-6*H*-dibenzo[*b*,*e*]oxepin-11-one (7f)

Color – yellow, yield 96%, mp 355–356 °C; ^1^H-NMR (400 MHz, CDCl_3_): *δ* 4.45 (s, 1H, N**H**), 4.83 (s, 2H, N–C**H**_**2**_), 5.14 (s, 2H, O–C**H**_**2**_), 6.94 (t, 2H, *J* = 8Hz, Ar–H), 7.15 (m, 3H, ArH), 7.39 (t, 1H, *J* = 8Hz, Ar–H), 7.51 (t, 1H, *J* = 8Hz, Ar–H), 7.63 (m, 3H, Ar–H), 7.94 (d, 1H, *J* = 8Hz, Ar–H). ^13^C-NMR: *δ* 50.84 (N–**C**H_2_), 73.62 (O–**C**H_2_), 127.62, 129.29, 129.42, 129.52, 129.86, 132.66, 135.85, 136.44, 142.68, 145.87, 146.45, 151.50 (aromatic region). HRMS calcd [M + H]^+^ for C_21_H_16_N_2_O_4_: 361.11, found: 361.1921.

#### 2.2.11 7-[(*tert*-Butylamino-methyl)]-6*H*-dibenzo[*b*,*e*]oxepin-11-one (7g)

Color – light brown, yield 87%, mp 233–236 °C; ^1^H-NMR (400 MHz, CDCl_3_): *δ* 1.35 (s, 9H, 3C**H**_**3**_), 3.92 (s, 1H, N**H**), 4.54 (s, 2H, N–C**H**_**2**_), 5.24 (s, 2H, O–C**H**_**2**_), 6.98 (m, 2H, Ar–H), 7.28 (t, 1H, *J* = 8Hz, ArH), 7.39 (t, 1H, *J* = 8Hz, Ar–H), 7.60 (t, 1H, *J* = 8Hz, Ar–H), 7.78 (d, 1H, *J* = 8Hz, Ar–H), 8.13 (d, 1H, *J* = 8Hz, Ar–H). ^13^C-NMR: *δ* 14.14 (*tert*-C), 22.71 (C–**C**H_3_), 29.17 (C–**C**H_3_), 31.63 (C–**C**H_3_), 76.71 (O–**C**H_2_), 114.08, 115.90, 123.49, 124.03, 135.16, 139.29, 142.80, 151.91 (aromatic region). HRMS calcd [M + H]^+^ for C_19_H_21_NO_2_: 296.16, found: 296.1453.

#### 2.2.12 7-(Allylamino-methyl)-6*H*-dibenzo[*b*,*e*]oxepin-11-one (7h)

Colour – light brown, yield 85%, mp 213–216 °C; ^1^H-NMR (400 MHz, CDCl_3_): *δ* 3.92 (t, 2H, *J* = 8Hz, C–C**H**_**2**_), 4.52 (d, 2H, *J* = 8Hz, N–C**H**_**2**_), 5.02 (s, 2H, N–C**H**_**2**_), 5.52 (s, 2H, O–C**H**_**2**_), 5.62 (d, 2H, allyl protons), 6.98 (t, 2H, *J* = 8Hz, Ar–H), 7.28 (m, 3H, ArH), 7.41 (t, 1H, *J* = 8Hz, Ar–H), 7.58 (t, 1H, *J* = 8Hz, Ar–H), 7.78 (m, 1H, Ar–H), 8.12 (m, 1H, Ar–H). ^13^C-NMR: *δ* 33.74 (N–**C**H_2_), 34.84 (N–**C**H_2_), 76.70, 114.07, 115.89, 123.48, 124.01, 128.61, 135.15, 139.27, 142.73, 151.97 (aromatic region). HRMS calcd [M + H]^+^ for C_18_H_17_NO_2_: 280.13, found: 280.1407.

#### 2.2.13 7-[(*p*-Methoxy-phenylamino-methyl)]-6*H*-dibenzo[*b*,*e*]oxepin-11-one (7i)

Color – dark brown, yield 94%, mp 373–375 °C; ^1^H-NMR (400 MHz, CDCl_3_): *δ* 3.52 (s, 3H, OC**H**_**3**_), 4.45 (s, 1H, N**H**), 4.95 (s, 2H, N–C**H**_**2**_), 5.25 (s, 2H, O–C**H**_**2**_), 6.94 (t, 2H, *J* = 8Hz, Ar–H), 7.10 (m, 2H, ArH), 7.39 (d, 2H, *J* = 8Hz, Ar–H), 7.54 (d, 2H, *J* = 8Hz, Ar–H), 7.73 (m, 3H, Ar–H). ^13^C-NMR: *δ* 35.00 (O–**C**H_3_), 54.46 (N–**C**H_2_), 74.25 (O–**C**H_2_), 118.02, 119.54, 126.92, 128.74, 130.78, 132.40, 132.23, 138.01, 139.53, 142.55, 144.01, 145.53 (aromatic region). HRMS calcd [M + H]^+^ for C_22_H_19_NO_3_: 346.14, found: 346.1453.

#### 2.2.14 7-(Octadecyl methyl)-6*H*-dibenzo[*b*,*e*]oxepin-11-one (7j)

Color – dark brown, yield 88%, mp 210–211 °C; ^1^H-NMR (400 MHz, CDCl_3_): *δ* 0.98 (m, 4H), 1.13–1.25 (m, 26H), 4.31 (s,N–H, 1H), 4.50 (s, 2H, oleyl chain N–CH_2_), 5.22 (s, 2H, O–CH_2_), 5.32 (m, 2H, –CH=CH–, oleyl chain), 7.12 (m, 3H, ArH), 7.53 (t, 2H, *J* = 8Hz, Ar–H), 7.82 (d, 1H, Ar–H, *J* = 8Hz), 8.11 (d, 2H, Ar–H, *J* = 8Hz). ^13^C-NMR: *δ* 11.42, 14.42, 19.73, 22.70, 26.85, 27.18, 29.38, 30.05, 32.76, 38.22 (aliphatic region), 121.3, 129.74, 129.98, 132.82, 161.20, 164.78 (carbonyl carbon). HRMS calcd [M + H]^+^ for C_33_H_49_NO_2_: 492.38, found: 492.3755.

## 3. *In silico* study

### 3.1 DFT study

A Density Functional Theory study is a computational quantum model used to investigate the electronic structure, energy levels, molecular orbitals, and mechanism of a compound. DFT study also helps to find out the theoretical NMR, FTIR, and UV-visible. The desired structure is first made in Gauss view 6.0 and set the calculation for optimization in Gaussian 09W at B3LYP/6-31+G(d,p) basis set. After optimizing the structure, the Potential energy surface (PES) study was performed on the flexible bonds by changing the bond angle from 90° to 120° by a transformation of 0.5° and the bond length of the NH bond by 0.05 Å each time of compound 7a.^15^ The frequency calculation was also done on the same basis set for analyzing the vibrations in a compound as FTIR spectra.^33,34^ No imaginary frequency in the output file approves the stationary state of the compound. Further, a UV-visible theoretical study was performed *via* the TD-SCF method at B3LYP/6-31+G(d,p) basis set.^35^ UV-visible and FTIR theoretical results were compared with experimental data.

### 3.2 Molecular docking

#### 3.2.1 Protein preparation

The crystal structure of different proteins DNA gyrase (PDB ID:1KZN, antibacterial), *N*-myristoyl-transferase (PDB ID:1IYL, antifungal), and DNA dodecamer (PDB ID:1BNA, ct-DNA activity) as drug targets were chosen from RCSB Protein Data Bank (https://www.pdb.org) were attained from RCSB Protein Data Bank.^36–38^ All crystal structures have more than 1 subunit of proteins, so all the chains except chain A, water molecules, and hetero atoms were removed. During the process, Kollman charges and polar hydrogen atoms were added to chain A which also includes some different amino acids for binding with ligands.

#### 3.2.2 Binding site

The binding site refers to the specific region of a target molecule, usually a protein, where a ligand binds. It is typically composed of a specific amino acid residue on the protein surface or within a cavity that interacts with the ligands through various forces. Table 2 mentions the binding sites of different proteins, such as clorobiocin, *N*-myristoyl-transferase, and DNA dodecamer.

#### 3.2.3 Validation of docking

The crystal structures of DNA gyrase and *N*-myristoyl transferase proteins are bound to chain A. The active sites were identified by consulting binding site information from the literature and visualizing it using Biovia Discovery Studio 2021.^39^ The docking procedure was validated by redocking the extracted DNA gyrase and *N*-myristoyl transferase substrate with chain A of the IKZN and 1IYL protein using the same coordinates. Docking accuracy was evaluated based on the RMSD score, which ideally should be less than 2 Å. This validation step is crucial for assessing the reliability of the chosen docking methodology.

#### 3.2.4 Docking procedure

The compounds were subjected to molecular docking using AutoDock 4.2. All the compounds and proteins were set in a cubic grid box having dimensions 60 Å (12.614*x*, 47.765*y*, −0.440*z*) for antifungal activity against *C. albicans* (PDB:1D:1IYL), 60 Å (19.577*x*, 19.108*y*, 43.257*z*) for antibacterial activity against *E. coli* (PDB:ID 1KZN), and 120 Å (15.091*x*, 21.550*y*, 8.822*z*) for ct-DNA (PDB:ID 1BNA) with a default spacing 0.375 Å. 10 conformations were obtained for every compound and a grid map was added using Autogrid 4.2. Docking calculation was performed by using AutoDock 4.2. The analysis of interactions between the protein and compounds was evaluated using Biovia Discovery Studio 2021.

### 3.3 ADME and physicochemical (drug likeliness) properties

ADME study analyses the chemical behavior of pharmacological compounds in a living organism. All the pharmacokinetic parameters were calculated from the PreADMET online server. This study helps to find out the detailed behavior of compounds by absorption, distribution, metabolism, and excretion in the body. These factors are crucial in drug development and its therapeutic effects and minimizing the side effects. Numerous parameters such as Blood–brain Barrier (BBB), Plasma Protein Binding (PPB), Skin permeability, *etc.*, are used to describe drug toxicity.^40^

Physicochemical parameters help to predict whether a compound has some features to become an effective drug or not. The parameters such as log *P*, PSA (Polar Surface Area), molecular weight, no. of heavy atoms, *etc.*, were calculated from https://www.molinspiratiom.com. All these parameters are a set of guidelines for Lipinski's rule that assess the drug likeliness of a compound. Any compound with only one violation of Lipinski's rule becomes an orally active drug.^41^

Bioactivity scores, such as those of GPCR ligands, kinase or protease inhibitors, and ion exchange modulators, illustrate the relationship between drugs and receptors. The mol-inspiration online server (https://www.molinspiration.com) helps to calculate the bioactivity score of ligands. Any drug is considered to be active if its bioactivity score is greater than 0.0, moderately active if its score lies between −5.0 and 0.0, and inactive if the score is lesser than −5.0.^42^

### 3.4 MD simulation

The optimal ligand 7a was subjected to molecular dynamics (MD) simulations by GROMACS (Version 2020) to assess the stability of docked complexes.^43^ The PRODRG server-generated ligand topology files, and GROMOS96_43a1 applied the force field for the simulations, which were conducted over 50 nanoseconds. The protein–ligand combination was solubilized in a cubic box utilizing the SPC (single point charge) water model. The system was neutralized by the addition of 4 Na^+^ ions.

To reduce solvent fluctuations and eliminate any residual strain, energy minimization was performed using the steepest descent algorithm with a maximum of 50 000 steps and a step size of 0.01. Following this, equilibration was conducted at 300 K using the NVT and NPT ensembles. The LINCS algorithm was employed to constrain protein bond lengths. The velocity-Verlet method, along with the Macro model, was used for the Particle-Mesh Ewald (PME) approach to compute long-range electrostatic interactions, with a cutoff distance of 1.2 nm for short-range electrostatic forces.

Temperature control at 300 K was maintained using the Berendsen thermostat, and pressure was held at 1 atm with the Parrinello–Rahman method to avoid any system clashes. Trajectory analysis was carried out using VMD, yielding RMSD, RMSF, hydrogen bond count, and the radius of gyration.^44^

## 4. *In vitro* studies

### 4.1 Anti-microbial study by zone of inhibition method

Antimicrobial activity refers to a substance's capability to prevent, kill, or inhibit the growth of microorganisms such as bacteria and fungi. The Kirby Bauer Disk Diffusion method was used to demonstrate this activity.^45^

#### 4.1.1 Antibacterial activity

The antibacterial activity of dibenzoxepinone derivative 7a was studied against Gram-positive and Gram-negative bacteria by the agar plate diffusion method. Culture was obtained from Microbial Type Culture Collection, Chandigarh, India – Gram-positive *Bacillus subtilis* (MTCC 441) and Gram-negative *Escherichia coli* (MTCC 443). Bacterial stock cultures were maintained on nutrient agar slants at 4 °C, and glycerol stocks were kept at −80 °C. Briefly, sterilized Nutrient Agar medium was poured into Petri dishes and solidified at room temperature. Using aseptic techniques, the agar plates were swabbed from an overnight bacterial primary culture to form a lawn. A sterile yellow tip (7 mm) was used to make the wells.

Compound 7a was tested against Gram-positive *Bacillus subtilis* at the following six dilutions (0, 2.5 μg, 5 μg, 12.5 μg, 25 μg, and 50 μg). For *E. coli,* compound 7a was prepared in three sets of dilutions (0, 25 μg, and 50 μg) for *E. coli*, prepared in DMSO of compound 7a, and added to the well of the Petri plates. After 50 μL of each was added to the well, the plates were incubated at 37 °C for 24 h. The diameter of clear zones (mm) around each well was measured. Ampicillin (5 mg ml^−1^) and DMSO were used as controls.^46^

#### 4.1.2 Antifungal activity

The antifungal activity was studied for the best binding compound 7a from molecular docking with antifungal protein *candida albicans* (MTCC 183). The fungal strain was obtained from the Microbial Type Culture Collections, Chandigarh, India. Various concentrations of test samples (25 μg and 50 μg) were prepared in DMSO. Fluconazole (100 mg ml^−1^) and DMSO were used as controls. Further, the plates were incubated at 30 °C for 20 hours. After incubation, the diameter of inhibition zones was measured in millimetres.^46^

### 4.2 Antibacterial study by RMDA method

The antibacterial activity of the synthesized compounds was evaluated using the Resazurin Microtiter Assay (RMDA). The assay was performed under aseptic conditions using a 96-well microtiter plate. In the first row of the plate, 100 μL of each test compound (7a–j) was added at an initial concentration of 1024 μg mL^−1^ in DMSO. Subsequently, 100 μL of nutrient broth was added to each well. A twofold serial dilution was carried out along each column by transferring 100 μL from one well to the next, resulting in a final minimum concentration of 0.5 μg mL^−1^.^47^

Following dilution, 10 μL of bacterial suspension adjusted to 0.5 McFarland standard (approximately 5 × 10^6^ CFU mL^−1^) was introduced into each well. The bacterial strains tested were *Lacto bacillus rhamnosus* (MTCC1408), *Bacillus subtilis* (MTCC 441), and Gram-negative *Escherichia coli* (MTCC 443), each prepared separately. To prevent dehydration of the cultures, the plates were sealed and gently wrapped with cling film. 30 μL of 5 mf per ml stock solution of ampicillin was injected as a positive control on each plate, and a negative control containing all reagents except the test compound was used.^48^

The plates were incubated at 37 °C for 18–24 hours to allow bacterial growth. After incubation, 100 μL of 0.01% resazurin solution was added to each well as a viability indicator, and the plates were incubated for an additional 30 minutes. Antibacterial activity was assessed visually based on a color change in the wells. Ampicillin was used as the reference antibacterial agent.

### 4.3 ct-DNA study

Ct-DNA activity is a biochemical and biophysical phenomenon that encompasses the interaction of ct-DNA with ligands to study the binding affinity. UV-visible absorption spectroscopy helps to identify the binding behavior of ct-DNA with compounds, and their interaction influences the absorbance property of the solution. A stock solution of ct-DNA (300 μM) was prepared in a Tris–HCl buffer solution containing 0.6 M HCl and 50 mM NaCl at pH 7.33. The UV absorbance ratio at 260 and 280 nm (*A*_260_/*A*_280_) was 1.9, confirming that the ct-DNA was free of protein contamination.^49^ The concentration of ct-DNA was determined from its absorbance at 260 nm, using a molar extinction coefficient of 6600 cm^−1^. A fixed volume (1 mL) of compounds 7a, 7b, and 7e of concentration 10 μM was titrated with increasing concentrations (20–100 μM) of ct-DNA. The binding constant *K*_b_ was calculated by the Benesi–Hilderbrand equation.^50^
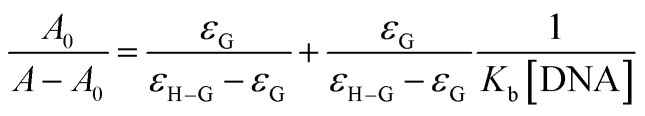
where *K*_b_ is the binding constant, *A* and *A*_0_ are the absorbance taken of ligands with ct-DNA at different concentrations and ligands without ct-DNA, respectively. *ε*_G_ and *ε*_H–G_ are the absorption coefficients taken of ligands with ct-DNA at different concentrations and ligands without ct-DNA, respectively. The binding energy was calculated by the ratio of intercept to the slope from the straight-line graph between *A*_0_/(*A* − *A*_0_) *vs.* 1/DNA.

## 5. Results and discussion

**Scheme 2:**
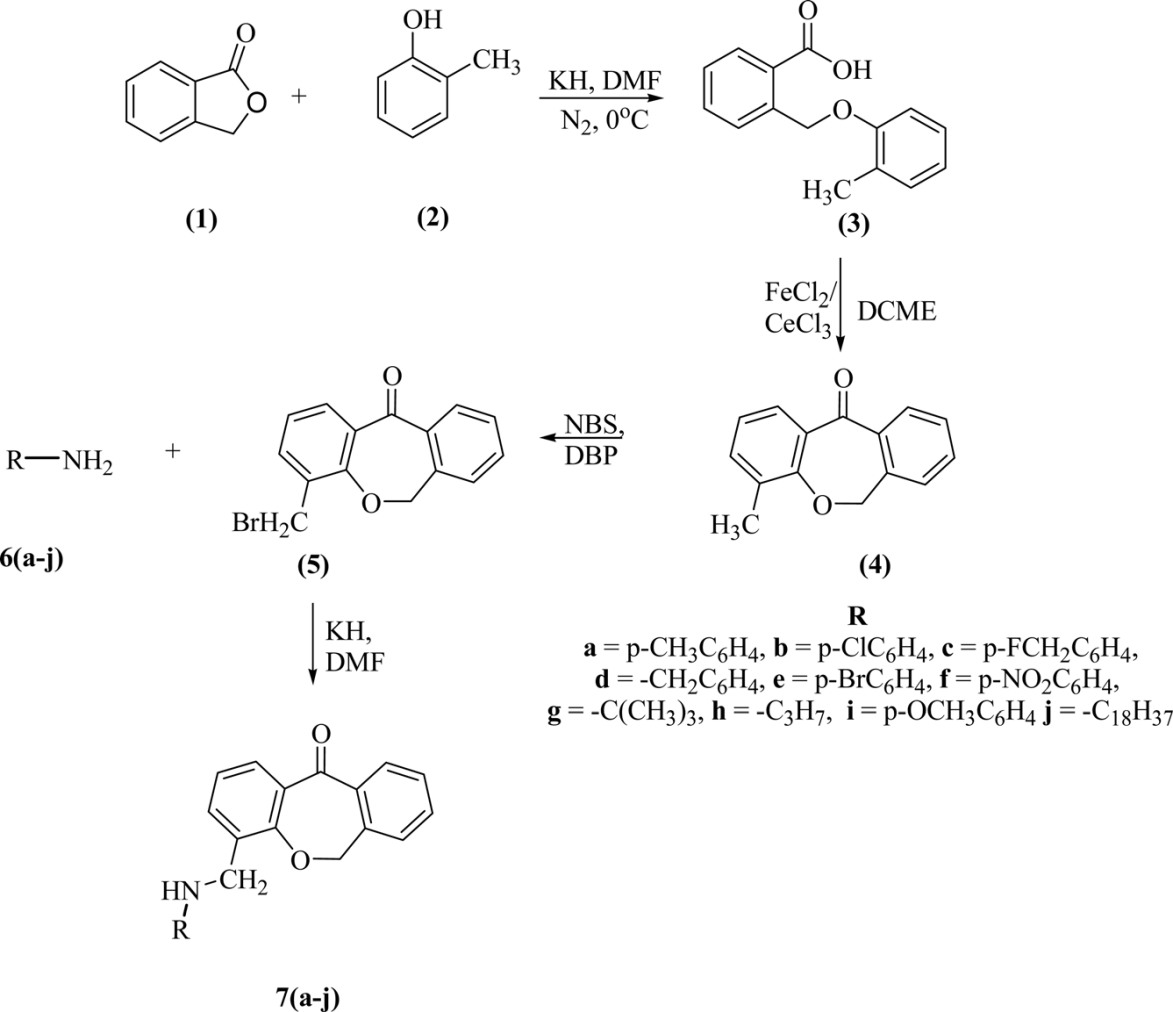
Synthesis of dibenzoxepinone derivatives.

We successfully synthesized ten different derivatives of dibenzoxepin-11-one, the synthetic protocol includes four steps (Scheme 2). In the first step, the intermolecular cyclization of the C–O bond or Williamson ether synthesis reaction was performed between phthalide (1) and *o*-cresol (2) to form 2-((*o*-tolyloxy)methyl)benzoic acid (3). The synthesized compound was characterized by ^1^H NMR and compared the peaks already given in the literature.^28^ The peaks at 5.52 ppm and 2.34 ppm, which correspond to carbon–oxygen bond formation as O–C**H**_2_ and –CH_3_ group of tolyl moiety respectively, confirmed the formation of 3. In the next step, the intramolecular cyclization of compound (3) in the presence of Lewis acid (FeCl_2_ or CeCl_3_) and DCME was done and the product obtained as dibenzoxepin-11-one (4), its spectral proton NMR data was compared with the given literature data, the peaks of O–CH_2_ at 5.52 ppm get shifted to the upfield range and observed at 5.22 ppm which confirmed that intramolecular cyclization has occurred. In the third step, the Wohl–Zeigler allylic bromination reaction of the methyl group present in tolyl ring was done. The peak for methyl group peak in 4, which was initially at 2.34 ppm get shifted to the downfield at 3.22 ppm due to the substitution of a bromine atom in place of hydrogen in compound 5, validating the progress of the reaction. The formation of 5 was further confirmed by ^13^CNMR and HRMS analysis. In the last step, the bromo group of 5 was treated with various substituted amines 6a–j. During this the CH_2_–Br peak was replaced by CH_2_–N– and the ^1^HNMR showed a downfield shift of methylene protons at 4.45 ppm in compound 7a. In all other compounds from (7b–j), this peak was observed between 4.0 ppm to 5.0 ppm. In FTIR spectrum of 7a, showed a signal at 3390 cm^−1^ frequency for the N–H bond, 1750 cm^−1^ represents the carbonyl group, 1290 cm^−1^ represents the N–CH_2_, 990 cm^−1^ represents C–O bond/ether linkage, and the other two peaks at 3010 cm^−1^ and 1590 cm^−1^ also verify the formation of compound 7a. Further, ^13^CNMR and HRMS confirmed the formation of the desired product.

### 5.1 DFT study

#### 5.1.1 Geometrical parameters

**Fig. 1:**
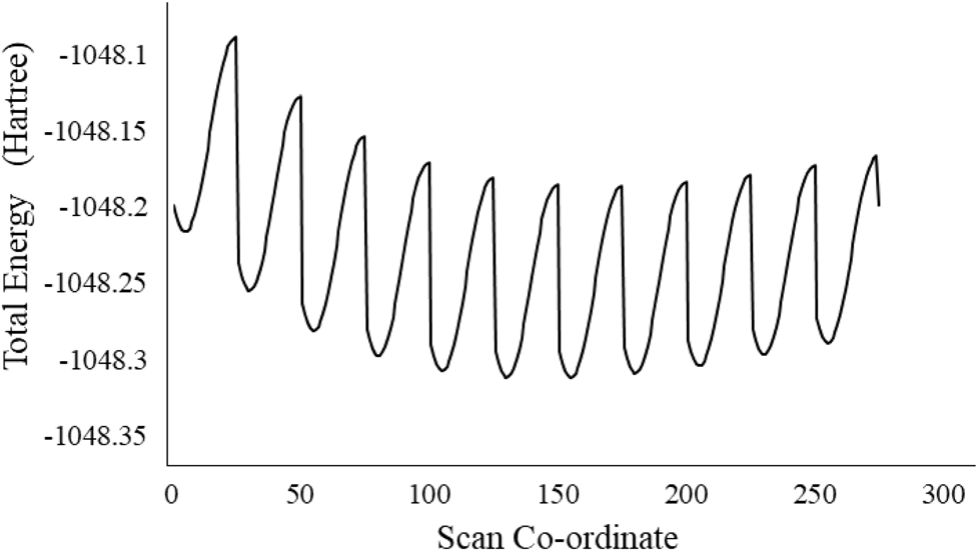
Potential energy scan of 7a.

**Fig. 2:**
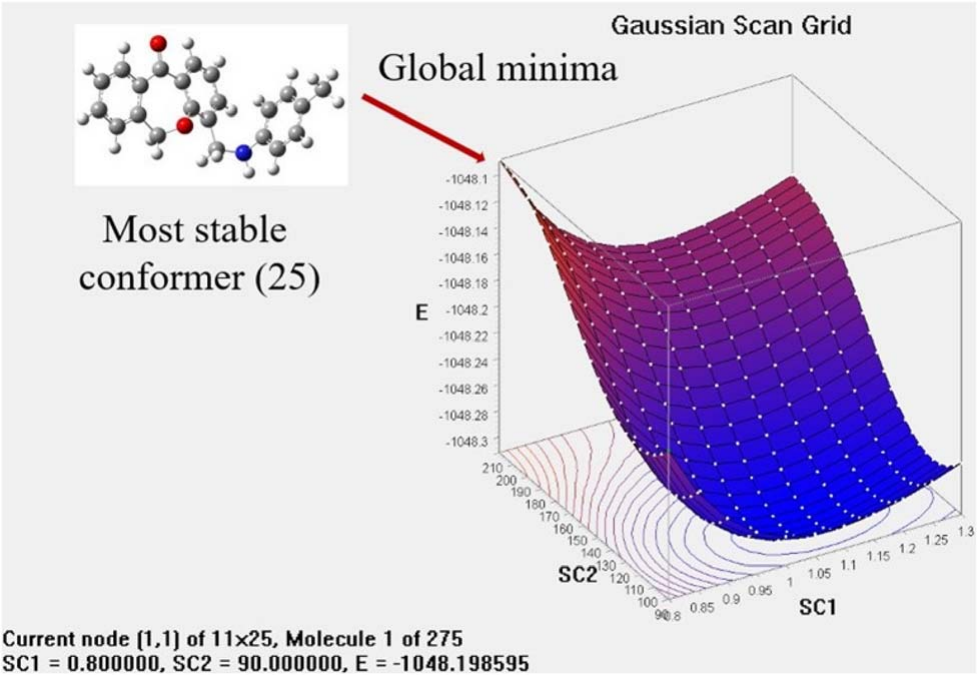
Potential energy scan grid of 7a.

The potential Energy Scan (PES) method is highly recommended for defining the molecular stability through different conformational analyses. It is done for molecule 7a by the DFT method and using a B3LYP/6-31+G(d,p) basis set. This analysis has shown 275 conformers by rotating the most flexible NH group by changing the bond angle by 0.5° from 90 to its absolute angle and bond length by 0.05 from 0.8 to its standard bond length. Each conformation has different energy and stability as shown in Fig. 1. The lowest energy of the compound was found to be −1048.311765 hartree. The lowest energy conformer (Fig. 2) was used for further calculations such as FTIR and UV-visible.

#### 5.1.2 Vibrational spectra analysis

**Fig. 3:**
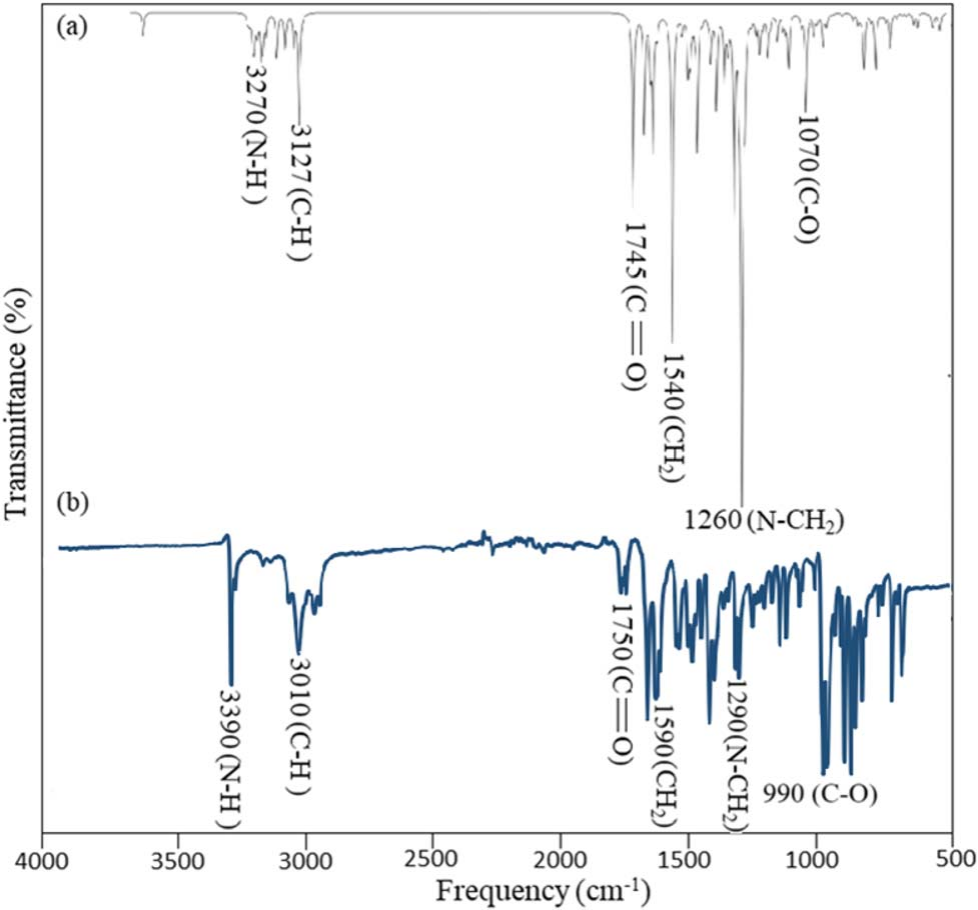
FTIR Data (a) theoretical data (b) experimental data.

FT-IR vibrational spectroscopy is a highly effective tool for the structural conformation of organic compounds. We have done theoretical and experimental FT-IR study for compound 7a. Theoretical FTIR was calculated at B3LYP/6-31+G(d,p) basis set. Compound 7a consists of 44 atoms and 126 vibrational modes, including three benzene rings, one oxepine ring with ketone group, one methyl group, one methylene group, and one N–H group. Oxygen and nitrogen atoms play an important role in structure elucidation. 7a shows C–H, N–H, C–O, carbonyl, C–N, and C–C stretching vibrations within the range of different frequencies mentioned in Fig. 3. The current study found theoretical C–H stretching vibration at 3127 cm^−1^, while the experimental FTIR band of C–H stretching at 3010 cm^−1^. N–H stretching vibration was observed at 3270 cm^−1^ at the B3LYP/6-31+G(d,p) basis set, and its experimental broadband was observed at 3390 cm^−1.^ Further carbonyl theoretical and experimental peak was found at 1745 cm^−1^ and 1750 cm^−1^. In addition, C–N, C–O, and C–C stretching vibrations were observed at 1260, 1070, and 1540 cm^−1^ theoretically with slight variation in the experimental peaks at 1290, 990, and 1590 cm^−1^ respectively. There was a slight variation in theoretical and experimental FTIR peaks due to the model used having the best conformation in theoretical FTIR while experimentally we can't control the change in conformations.^33,34^

#### 5.1.3 Electronic properties

**Fig. 4:**
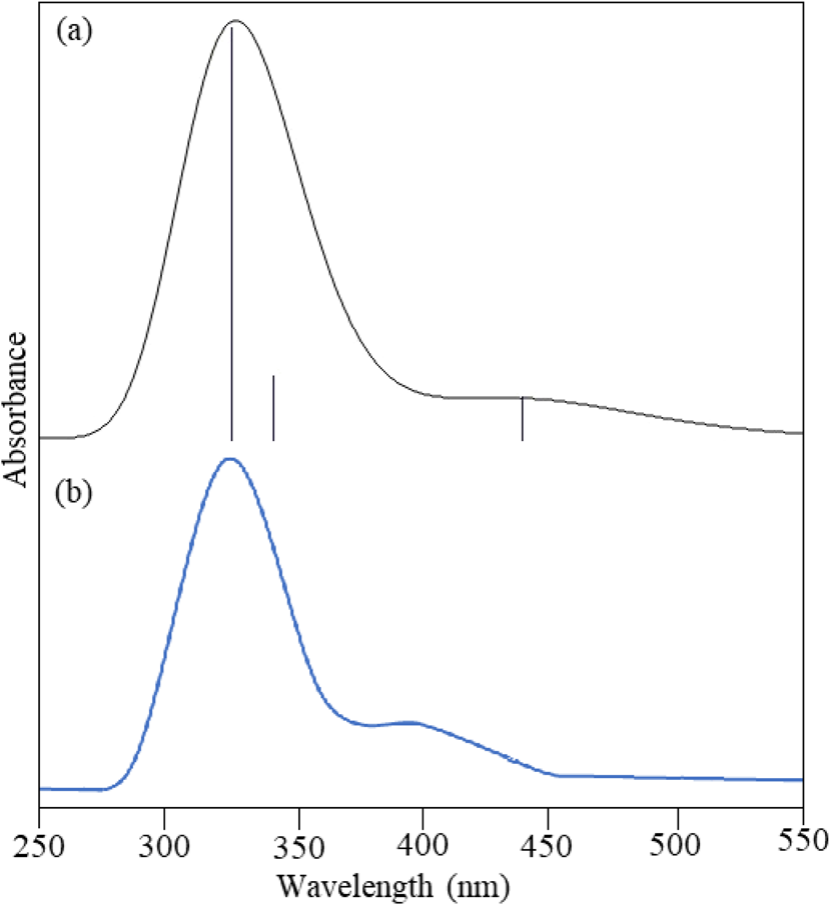
UV-visible spectrum (a) theoretical (b) experimental.

**Fig. 5:**
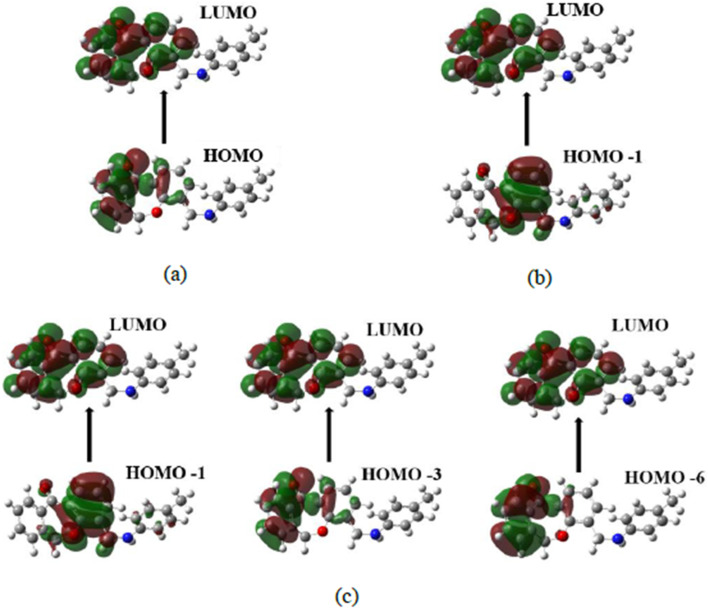
Electronic transition orbitals in UV-visible (a) first excitation (b) second excitation (c) third excitation.

The theoretical UV-vis spectrum for compound 7a was performed using TD-DFT/6-31+G(d,p) basis set by employing the solvent DMSO. An experimental UV-vis spectrum was also done in DMSO. The electronic transition orbitals are shown in Fig. 5. Experimental and theoretical calculations both measure absorption wavelength (*λ*) and excitation energy (*E*) during the electronic transition of an electron from the ground state to the excited state depicted in Table 1 and Fig. 4. The theoretical adsorption values were observed at 457.29, 350.30, and 332.44 nm associated with 0.0039, 0.0057, and 0.0367 oscillator frequencies. The first electronic transition was observed from HOMO > LUMO having a percentage coefficient of 99%. The second electronic transitions were found from HOMO-6 > LUMO, HOMO-3 > LUMO, and HOMO-1 > LUMO with a percentage coefficient of 10.33%, 83.26%, and 2.4%. The last electronic transition was observed from HOMO-1 > LUMO with 94.01%. The experimental UV showed a prominent absorbance at 330.10 nm and a smaller absorption near 373.56 nm.^35^

**Table 1:** UV-visible absorption spectra details

Experimental		Theoretical/TD-DFT/6-31+G(d,p)		
*λ*_cal_ (nm)	Band gap (eV)	*λ*_cal_ (nm)	Band gap (eV)	*F* (frequency)
330.10	3.2146	332.44	3.7295	0.0367
373.56	2.9752	350.30	3.5393	0.0057
		457.29	2.7113	0.0039

### 5.2 Molecular docking

#### 5.2.1 Active site of protein and validation of docking

**Fig. 6:**
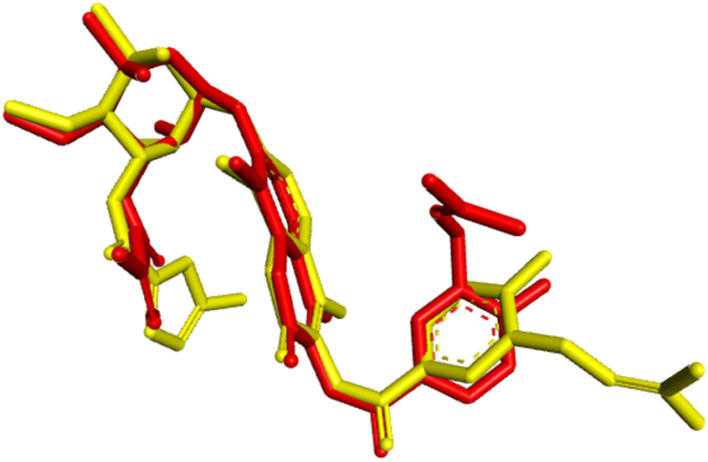
Validation of the docking methodology by superimposing re-docked chlorobiocin (yellow color) with co-crystallized protein complex (red) with a RMSD: 1.54 Å.

**Fig. 7:**
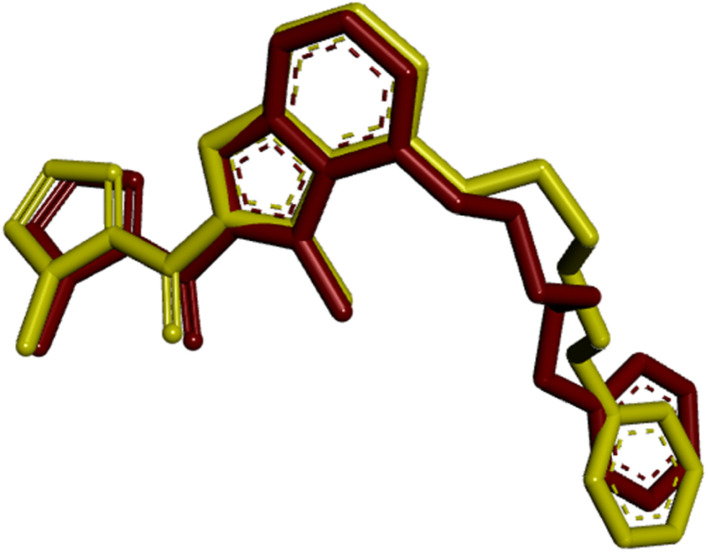
Validation of the docking methodology by superimposing re-docked *N*-myristoyl transferase (yellow color) with co-crystallized protein complex (dark red) with a RMSD: 0.98 Å.

The functionality of a protein is largely determined by the active binding site. To investigate and identify the active site residues involved in docking, the substrate chlorobiocin and *N*-myristoyl-transferase were utilized. This substrate was obtained from PDB:1D 1KZN and 1IYL, accessed *via* the RCSB PDB database. The active binding site, comprising the amino acid residues listed in Table 2, was analyzed. The same docking methodology was applied for validation. The re-docked complex and the co-crystallized complex of the antibacterial protein and antifungal protein was superimposed using Biovia Discovery Studio 2021 (version 21.2). Root-mean-square (RMSD) values of 1.54 Å and 0.98 Å were obtained, as illustrated in Fig. 6 and 7 demonstrating the high precision in the docking procedure.

**Table 2:** Amino acids present at the active binding site for antimicrobial activities

PDB:ID	Amino acids at the active site
1KZN	TYR119, TYR225, TYR107, TYR119, PHE339, PHE117, PHE240, PHE176, ILE111, ILE352, LEU451, LEU394, HIS227, CYS393, ASP110, ASN175, ASN392
1IYL	ARG76, ARG136, ARF186, ARG22, TYR26, TYR165, ILE90, ILE78, VAL71, ASP73, ASN46, PRO79, HIS95, GLU50, GLY77

#### 5.2.2 Antibacterial molecular docking

All the synthesized compounds (7a–j) were docked with *E. coli* antibacterial PDB Id:1KZN to examine the interaction between the compounds and DNA gyrase protein (PDB Id:1KZN). Antibacterial protein DNA gyrase is critical for maintaining DNA supercoiling, which is vital for replication, transcription, and other cellular functions. Clorobiocin works by binding to the ATP-binding site of the GyrB subunit, blocking ATP hydrolysis a key step in the enzyme's activity of essential type II topoisomerase.^51^

**Fig. 8:**
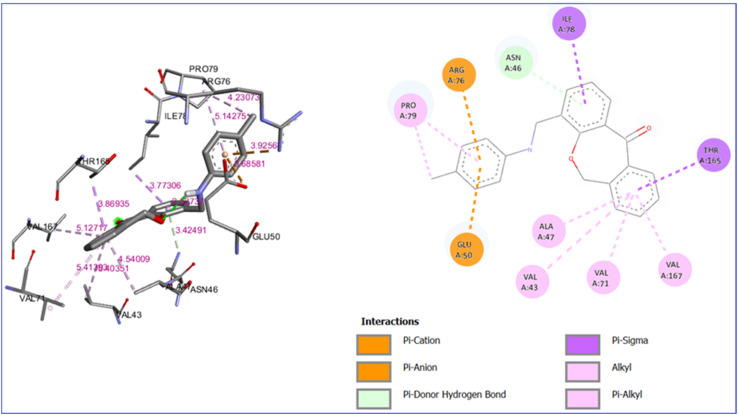
3D and 2D interaction diagram of compound 7a with *E. coli* bacterial protein (PDB ID:1KZN).

This study revealed that all the compounds showed good binding interaction with different amino acids in the protein with different bonds. The binding energy range of the compounds with protein lies between −6.0 to −9.2 kcal mol^−1^. The maximum binding energy −9.2 kcal mol^−1^ was observed for compound 7a, as mentioned in Table S1,† which has a Pi-donor hydrogen bond with ASN 46, a Pi-sigma bond with ILE 78, and THR 165. Some of the interactions with bond length greater than 5 Å due to hydrophobic interactions but they still contribute to ligand binding in long range binding interactions particularly between the charged residues and ligands. All other binding interactions are shown in Fig. 8. The interaction diagram of the remaining compounds was shown in Fig. S1† to S9.

**Fig. 9:**
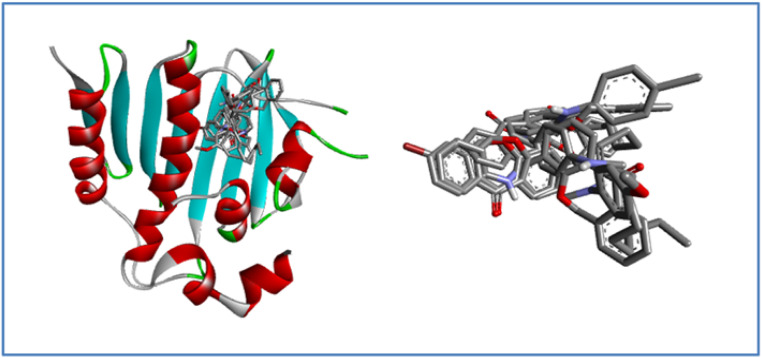
Superimposition of docked ligand **7a–j** conformations within the active site of the *E. coli* bacterial protein.

Fig. 9 represents that all the ligands superimposing in the active site of the target after docking, it indicates a consistent binding mode and a well-defined binding pocket. This suggests that all derivatives interact with the same key residues, implying a common mechanism of action. Such consistency strengthens the reliability of the docking predictions and suggests that the ligands may exhibit similar biological activity.

#### 5.2.3 Antifungal molecular docking

The molecular docking for all the compounds (7a–j) was done with *Candida albicans* protein *N*-myristoyl transferase (PDB ID:1IYL). *N*-Myristoly transferase (NMT) is an essential enzyme that catalyzes the covalent attachment of a 14-carbon fatty acid, myristate, to the N-terminal glycine residue of specific proteins in a co-translational and post-translational manner.

**Fig. 10:**
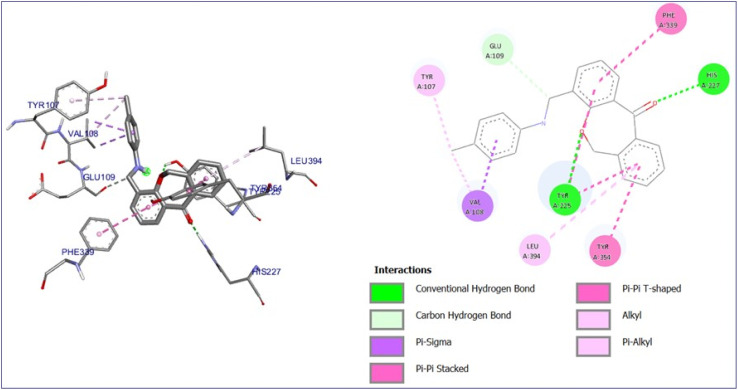
3D and 2D interaction diagram of compound 7a with antifungal protein (PDB ID:1IYL).

Its single-subunit enzyme facilitates the attachment of the fatty acid myristate from myristoyl-CoA to the N-terminal glycine residue of various eukaryotic and viral proteins. PDB 1D:1IYL represents the crystal structure *N*-myristoyl transferase, which is an essential enzyme in ergosterol biosynthesis, and is a crucial component of fungal cell membranes. Inhibiting this enzyme can hinder ergosterol, weakening cell membrane integrity and ultimately causing cell death in fungi. The binding energy of the compounds with the protein falls in the range of −8.0 to −11.0 kcal mol^−1^ noted in Table S1.† Affluence the capacity of cell death in fungi. 7a compound has the maximum binding energy (−11.0 kcal mol^−1^) with some imperative bonds such as conventional hydrogen bonds with TYR 225 and HIS 227, carbon–hydrogen bond with GLU 109, Pi-sigma bond with VAL 108, alkyl and Pi-alkyl bond with LEU 394 and TYR 107 as shown in Fig. 10. Interactions of compounds 7b–j with protein are depicted in Fig. S10 to S18.†

**Fig. 11:**
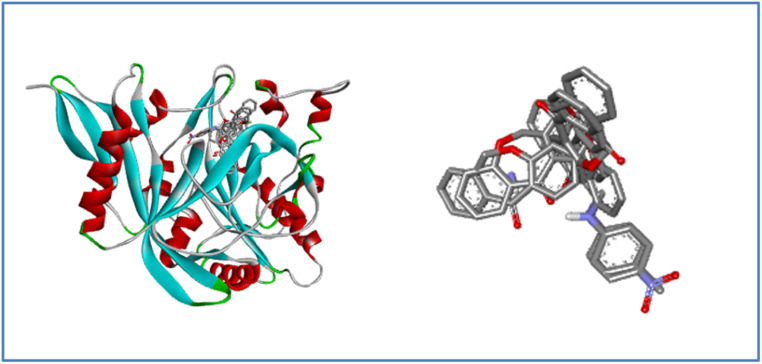
Superimposition of docked ligand 7a–j conformations within the active site of the antifungal protein *N*-myristoyltransferase.

Fig. 11 illustrates the superimposition of all ligands within the active site of the target after docking, demonstrating a consistent binding mode and a well-defined binding pocket. This indicates that all derivatives interact with the various amino acid residues, suggesting a similar mechanism of action. The high degree of overlap reinforces the reliability of the docking predictions and implies that the ligands may possess similar biological activity.

#### 5.2.4 Molecular docking with ct-DNA

**Fig. 12:**
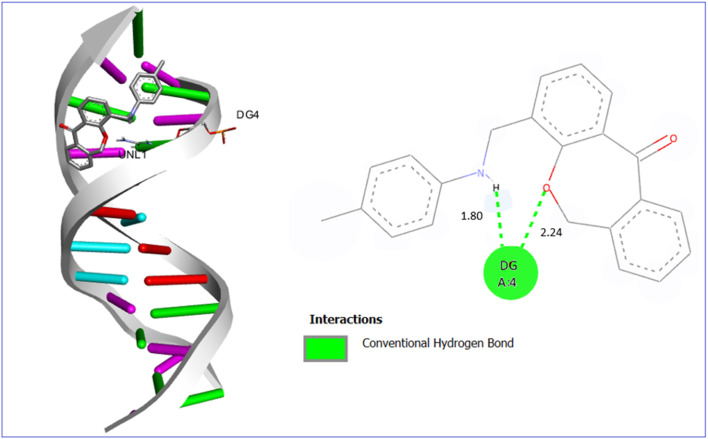
3D and 2D interaction diagram of compound 7a with ct-DNA (PDB ID:1BNA).

The molecular docking outcomes demonstrated that the synthesized derivative 7a preferably binds with an AT-rich groove of the DNA sequence rather than to the terminal G-C. A DNA dodecamer is a short oligonucleotide of 12 base pairs, often employed as a model system in structural and biochemical studies of DNA. Ligand 7a was complemented by the natural curvature of the DNA dodecamer, forming a groove combination mode as shown in Fig. 12.

Conventional hydrogen bonding played a significant role in the interaction of ligand 7a with DNA through a maximum binding energy of −8.1 kcal mol^−1^. The conventional hydrogen bonds were formed between carboxylic acid and guanosine of A strand with a bond length of 2.24 Å and the NH group of the ligand and guanosine of A strand with a bond length of 1.80 Å as noted in the 2D diagram. All other ligands also showed groove binding with different bonding interactions and binding energies as mentioned in Table S1 and Fig. S19 to S27.†

**Fig. 13:**
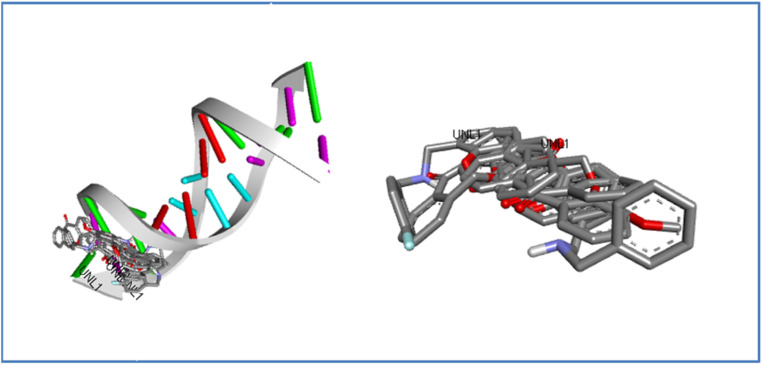
Superimposition of docked ligand 7a–j conformations within the active site of DNA protein dodecamer.

Fig. 13 depicts the superimposition of all ligands within the target's active site after docking, highlighting a consistent binding mode and a well-defined binding pocket. This suggests that all derivatives interact with the same key residues, indicating a shared mechanism of action. The significant overlap enhances the credibility of the docking predictions and suggests that the ligands may exhibit similar biological activity. Additionally, the superimposition reinforces the strong affinity of the binding site for these ligands, making it a promising candidate for further drug optimization.

The docking results show that all the synthesized derivatives were showing good binding interaction with all the PDB IDs:1KZN, 1IYL, AND 1BNA having range lie between −6.0 to 9.2 kcal mol^−1^ for antibacterial target protein, −8.5 to −11.0 kcal mol^−1^ for antifungal target protein, and −5.3 to −8.3 kcal mol^−1^ for DNA gyrase, but compound 7a has maximum binding energy with all target proteins that include different interactions like conventional hydrogen bond, carbon hydrogen bond, pi-sigma, and pi-alkyl, *etc.* Maximum bonding interactions of compound 7a were observed with the target protein 1IYL, and 1BNA shows the best binding with it and good biological activity.

### 5.3 ADME and physicochemical parameters

For drug development, synthesized derivatives should have a good ADME profile. ‘Lipinski's rule of five’ parameters such as (i) MW under 500, (ii) no. of hydrogen bond less than 5, (iii) no of hydrogen bond acceptors less than 10, (iv) log *P* value less than 5, (v) total polar surface area (TPSA) should not be greater than 140 Å^2^, indicates that the molecule is likely to be an orally active drug or not. Additionally, one violation is acceptable. All the molecules obey Lipinski's rule except 7b and 7c, they both have one violation. Other ADME parameters like blood–brain barrier (BBB), CaCo_2_, HIA, and protein plasma binding lie in the range of active drug noted in Table S3,† which means that compounds 7a–j behave as orally active drug.

A bioactivity score of less than −5.0 suggests the compound is inactive, a score between −5.0 and 0.0 indicates slight activity, and a score greater than 0.0 signifies high activity. All synthesized derivatives fall in the range and exhibit good bioactivity scores in Table S4.†

### 5.4 MD simulation

The dynamic stability of the optimal dibenzoxepine complex with the antibacterial protein and antifungal protein (PDB ID:1KZN and 1IYL) was assessed through MD simulation using Gromacs. Ligand topology was generated *via* the free online ATB server, and the GROMOS96 force field was applied to the simulated complex. The dibenzoxepine–protein complex was initially positioned within a cubic simulation box, followed by energy minimization in a solvated system. After energy minimization, MD simulations were conducted for 50 ns under constant conditions of 300 K temperature and 1 atm pressure.

#### 5.4.1 Molecular dynamics (MD) simulation study of 7a in complex with antibacterial protein

##### 5.4.1.1 Root mean square deviation (RMSD)

**Fig. 14:**
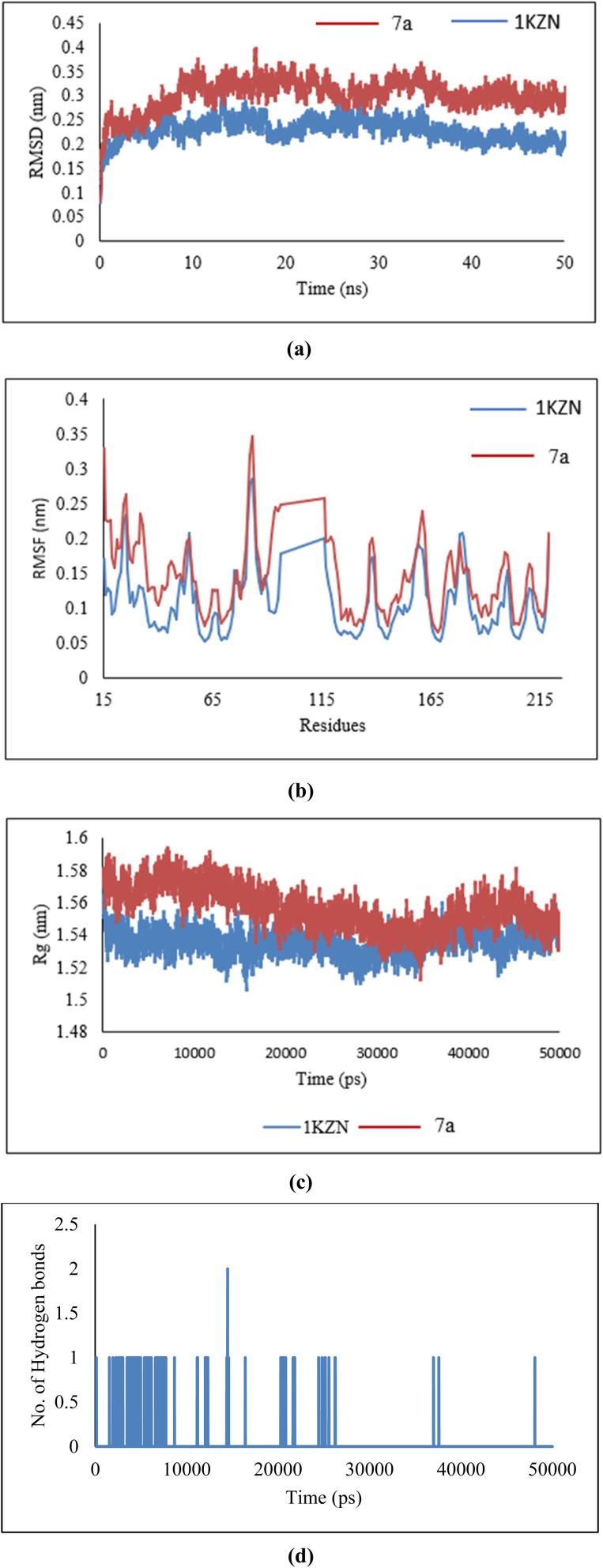
(a) RMSD, (b) RMSF, (c) radius of gyration, and (d) hydrogen bonds of 7a ligand in complex with 1KZN protein.

The interaction between the backbone atoms of the native 1KZN protein and its complex with 7a was analyzed using the Root Mean Square Deviation (RMSD) method. A stable RMSD pattern was observed for 50 ns. However, variations in RMSD values were noted, ranging from 0.1 nm to 0.39 nm for the native 1KZN protein (Fig. 14a). These minor variations reflect the stability of the protein–ligand complex.

##### 5.4.1.2 Root mean square fluctuation (RMSF)

The residue-wise variations were analyzed using Root Mean Square Fluctuation (RMSF). As shown in Fig. 14b, the 7a–protein complex exhibited fluctuations comparable to those of the native 1KZN structure, suggesting that the complex maintained significant stability due to ligand interactions.

##### 5.4.1.3 Radius of gyration (*R*_g_)

The structural compactness of the protein was assessed using the Radius of Gyration (*R*_g_). A consistent pattern was observed, with *R*_g_ values ranging from 1.51 nm to 1.61 nm (Fig. 14c) when comparing the native 1KZN protein with its ligand-bound complex. These results indicate the formation of a stable complex characterized by high structural compactness.

##### 5.4.1.4 Hydrogen bonds

The analysis of antibacterial activity demonstrated a consistent and significant number of hydrogen bonds within the 7a–1KZN complex throughout the 50 ns MD simulation (Fig. 14d). The presence of these hydrogen bonds confirmed the structural integrity of the protein, strong protein–ligand interactions, and overall stability of the complex.

#### 5.4.2 Molecular dynamics (MD) simulation study of 7a in complex with antifungal protein

##### 5.4.2.1 Root mean square deviation (RMSD)

**Fig. 15:**
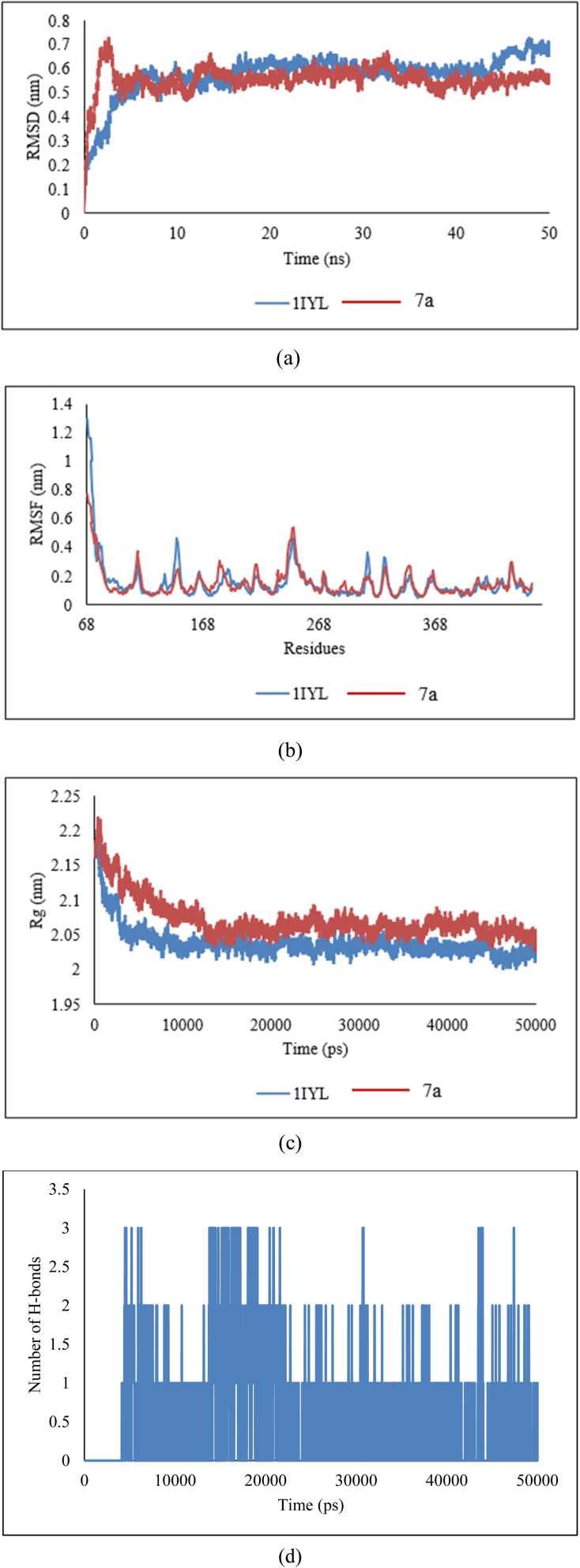
(a) RMSD, (b) RMSF, (c) radius of gyration, and (d) hydrogen bonds of 7a ligand in complex with 1IYL protein.

The interaction between the backbone atoms of the native 1IYL protein and its complex with 7a was evaluated using the Root Mean Square Deviation (RMSD) method. A stable RMSD pattern was observed over the 50 ns simulation period, with variations ranging from 0.1 nm to 0.34 nm for the native IYL protein (Fig. 15a). These minor deviations highlight the stability of the protein–ligand complex.

##### 5.4.2.2 Root mean square fluctuation (RMSF)

Residue-wise variations were analyzed using the Root Mean Square Fluctuation (RMSF) method. As depicted in Fig. 15b, the 7a–protein complex displayed fluctuations similar to those of the native 1IYL structure, indicating significant stability of the complex due to ligand interactions.

##### 5.4.2.3 Radius of gyration (*R*_g_)

The structural compactness of the protein was analyzed using the Radius of Gyration (*R*_g_). A consistent trend was observed, with *R*_g_ values ranging from 2.01 nm to 2.75 nm (Fig. 15c) for both the native 1IYL protein and its ligand-bound complex. These findings confirm the formation of a stable complex with high structural compactness.

##### 5.4.2.4 Hydrogen bonds

The evaluation of antibacterial activity revealed a stable and substantial number of hydrogen bonds within the 7a–1IYL complex during the 50 ns MD simulation (Fig. 15d). These hydrogen bonds underscore the structural integrity of the protein, robust protein–ligand interactions, and overall stability of the complex.

### 5.5 Antimicrobial activity

#### 5.5.1 Zone of inhibition method

The findings from the antibacterial and antifungal activity assay revealed that compound 7a demonstrated differing levels of inhibition against Gram-positive *B. subtilis*, Gram-negative *E. coli*, and *C. albicans* strains, as shown in Fig. S28.† The assessment included measuring the sizes of the inhibitory zones, encompassing the diameter of the disk, to gauge the compound's efficacy in hindering bacterial and fungus growth.

Moreover, the zones of inhibition results revealed the compound's potential to inhibit the growth of bacteria and fungus strains. Thus, emphasizing that the synthesized compound 7a is a bioactive compound with significant antimicrobial potential leading to growth inhibition. The diameter of the zone of inhibition increases as the concentration of compound 7a increases (Table 3).

**Table 3:** Diameter of the zone of inhibition for antimicrobial activity of compound 7a

Concentration (μg)	Diameter of zone of inhibition (mm)		
	*E. coli*	*Bacillus subtilis*	*Candida albicans*
5	—	—	—
12.5	—	12	—
25	16	15	15
50	17	17	18
Ampicillin^a^	20	24	—
Fluconazole^a^	—	—	15

a Positive control.

#### 5.5.2 RMDA method

As the compound 7a showed the good zone of inhibition for different bacterial strains, further all the synthesized derivatives were tested against three bacterial strains (*Lacto bacillus rhamnosus* MTCC1408, *Bacillus subtilis* MTCC 441, and *E.coli* MTCC 443) for MIC evaluation. The outcomes of the antibacterial evaluations demonstrated that compounds 7a, 7b, 7c, and 7i exhibited lowest MIC value against both the Gram-positive bacterial strains *Lacto bacillus rhamnosus* MTCC1408 and *Bacillus subtilis* MTCC 441. However, 7a and 7c have lowest MIC value for Gram negative strain *E. coli* MTCC 443 (Table 4 and Fig. S29†). Resazurin (7-hydroxy-3*H*-phenoxazin-3-one-10-oxide), a blue dye with slight fluorescence, was utilized as an oxidation-reduction indicator in the assay. This dye is commonly used in cell viability assessments for both microbial and mammalian cells. A positive antibacterial response was indicated by a color change from blue or purple to pink, resulting from bacterial metabolic activity.

**Table 4:** MIC values of synthesized derivatives against different bacterial strains

MIC values (μg ml^−1^)	7a	7b	7c	7d	7e	7f	7g	7h	7i	7j
*L. rhamnosus*	8	8	8	16	64	8	32	32	8	128
*B. subtilis*	16	16	16	32	16	128	128	16	16	256
*E. coli*	8	16	8	16	16	32	32	64	16	128

### 5.6 ct-DNA binding activity

#### 5.6.1 UV-visible absorption

**Fig. 16:**
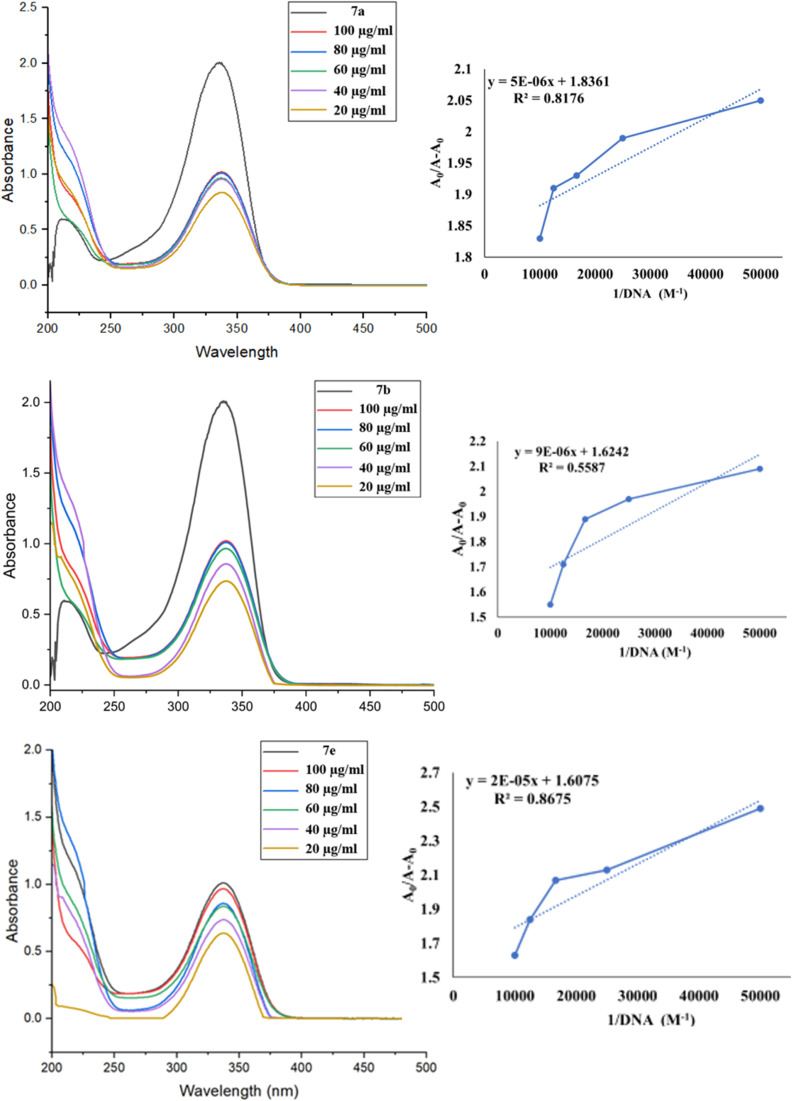
UV-Vis spectral changes were observed in 7a, 7b, and 7e at a concentration of 10 μM in 50 mM Tris–HCl/NaCl buffer (pH = 7.35) at 25 °C, as the ct-DNA concentration increased from (20–100 μM). The arrow indicates a decrease in absorbance as the ct-DNA concentration increases.

The UV-visible absorption method is valuable for the qualitative and quantitative analysis of ct-DNA binding with synthesized dibenzoxepinones. This method often reveals the change in absorption, which can manifest as hyperchromic and hypochromic, along with shifts in the maximum absorption wavelength, indicating blue and red shifts. In the present study, the synthesized compounds 7a, 7b, and 7e were chosen for study to bind with ct-DNA. The UV spectrum of compounds was recorded, showing a peak at 330 nm absorbance and was attributed to the intra-ligand π–π* electron transition within the C–N system. The gradual addition of ct-DNA, leading to a hypochromic shift, and the absence of the red or blue shift in the binding of ct-DNA with ligands was groove binding (Fig. 16).

The intrinsic binding constant (*K*_b_) was evaluated from intercept by slope ratio from the equivalent double reciprocal graph of *A*_0_/*A* − *A*_0_ against 1/[DNA] mentioned in Table 5. The hypochromic effects of compounds with ct-DNA and the *K*_b_ values confirmed their stable interaction.

**Table 5:** Binding constants (*K*_b_, M^−1^), and Gibbs free energy (Δ*G*, kJ mol^−1^) of compounds

Compounds	7a	7b	7e
Binding constant (*K*_b_)	3.61 × 10^5^	1.80 × 10^5^	8.03 × 10^4^
Gibbs free energy (Δ*G*)	−31.70	−29.98	−27.98

The Gibbs free energy (Δ*G*) of the compounds (7a, 7b, 7e)/ctDNA complex can account for the complex's stability was assessed when ct-DNA was introduced into the solution. The equation below was used to calculate the free energy of the system utilizing the relevant parameters:Δ*G* = −*RT* ln(*K*_b_)

The analysis of the Gibbs free energy cited in Table 5 for the complexes revealed that the binding of compound 7a with ct-DNA was thermodynamically more stable.

#### 5.6.2 Fluorescence quenching studies of compound 7a/ct-DNA

**Fig. 17:**
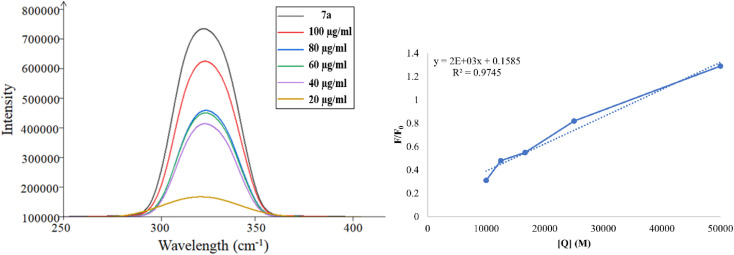
Fluorescence emission spectra of 7a (10 μM) with an increasing concentration of ct-DNA (0–100 μM). The inset shows the Stern–Volmer constant (*k*_sv_). The arrow shows a decrease in intensity with an increase in ct-DNA concentration.

The UV study indicated that 7a was the best binder of ct-DNA, therefore further study has been performed for UV Fluorescence quenching. The fluorescence quenching study of 7a was analyzed to investigate its binding interaction with DNA, given that DNA itself exhibits weak natural fluorescence. Fig. 17 presents the fluorescence emission spectrum resulting from the titration of 7a with ct-DNA using the excitation wavelength of 330 nm. The observed decrease in peak intensities upon the addition of ct-DNA intensities suggests the formation of a stable complex and enhanced interaction between ct-DNA and 7a.

Fluorescence quenching is a process where the fluorescence intensity of a substance diminishes, typically occurring due to various molecular interactions such as ground state complex formation, excited state reactions, and molecular rearrangement. The effectiveness of fluorescence quenching can be assessed using the Stern–Volmer equation to determine the Stern–Volmer quenching constant (*K*_sv_):
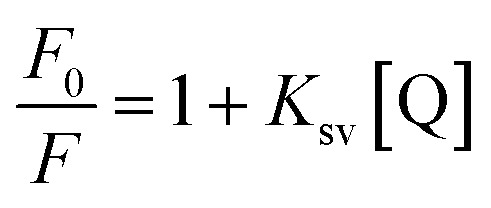


In this context, *F*_0_ and *F* represent the fluorescence intensities in the absence and presence of the quencher, respectively while [Q] denotes the quencher concentration. The stern–Volmer quenching constant (*K*_sv_) is obtained from the slope of the *F*_0_/*F vs.* [ct-DNA] plot. For compound 7a, the *K*_sv_ value was determined to be 2.0 × 10^3^ M^−1^, suggesting a non-intercalative binding mode, as this value is lower than those typically associated with intercalators. The distinction between static and dynamic quenching can be determined by examining the linearity of the Stern–Volmer plot and applying the relevant equation.
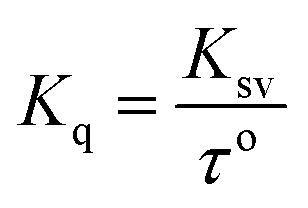


In this equation, *K*_q_ represents the biomolecular quenching rate constant and *τ*^o^ is the biomolecule's fluorescence lifetime of the biomolecule in the quencher's absence. With *τ*^o^ at 10^−8^ s, *K*_q_ was calculated to be 2.0 × 10^11^ M^−1^ s^−1^. A high *K*_q_ value suggests that the quenching occurs *via* static quenching through complex formation rather than dynamic quenching. Hence, compound 7a can bind with ct-DNA at the groove binding site.

## Conclusion

Ten novel derivatives of dibenzoxepine-11-one were synthesized and demonstrated the ability to bind effectively with key biological targets, including bacterial DNA gyrase (PDB ID:1KZN, *E. coli*), *N*-myristoyltransferase (PDB ID:1IYL, *C. albicans*), and the DNA dodecamer (PDB ID:1BNA, ct-DNA). Molecular docking studies revealed favorable binding energies across all targets, with compound 7a exhibiting the highest binding affinities of −9.2 kcal mol^−1^ with *E. coli*, −11.0 kcal mol^−1^ with *C. albicans*, and −8.1 kcal mol^−1^ with ct-DNA. The *in vitro* antimicrobial activity was done compound 7a against *B. subtilis*, *C. albicans*, and *E. coli*. At a concentration of 50 μg, compound 7a showed the highest zone of inhibition, with diameters of 17 mm against the bacterial strains and 18 mm against the fungal strain comparable to the standard antimicrobial agents Ampicillin and Fluconazole. These results were further validated for all the compounds by RMDA method to find MIC values against Gram-positive strains (*B. subtilis*, *L. rhamnosus*) and a Gram-negative strain (*E. coli*). Compounds 7a, 7b, 7c, and 7i demonstrated notable antibacterial potency, exhibiting lowest minimum inhibitory concentration (MIC) values for the Gram-positive strains, however, 7a & 7c have lowest MIC values for both Gram-positive and negative strains. Additionally, DNA-binding studies with calf thymus DNA on compounds 7a, 7b, 7e, again confirmed that compound 7a was the most potent groove binder, with a binding constant (*K*_b_) of 3.61 × 10^5^ M^−1^ and a Gibbs free energy of −31.70 kJ mol^−1^. Overall, compound 7a emerged as the most promising candidate, showing superior performance across all *in vitro* evaluations, which was further validated by *in silico* approaches, including DFT calculations and molecular dynamics simulations.

## Abbreviations

DFT: Density functional theory
ct-DNA: Calf thymus seoxyribose nucleic acid
PSA: Polar surface area
UV: Ultra-violet
BBB: Blood–brain barrier

## Data availability

The authors confirm that the supporting findings of this study are available within the article and are ESI.†

## Author contributions

Dr Leena Khanna and Prof Pankaj Khanna: conceptualization of idea, supervision. Shilpa Yadav, Asmita Singh: literature search and primary draft and drawing of figures and tables. Shilpa Yadav, Priyanshu, Pratibha Chanana: performed *in vitro* experiments. Shilpa Yadav & Mansi: DFT study, execution of molecular docking and simulation of ligands. All authors reviewed the manuscript.

## Conflicts of interest

The authors state no conflict of interest.

## Supplementary Material

RA-015-D5RA01068C-s001
