# Correction: Synthesis and multifaceted exploration of dibenzoxepinones: *in vitro* antimicrobial and ct-DNA binding, DFT/TD-DFT, molecular docking and simulation studies

**DOI:** 10.1039/d6ra90029a

**Published:** 2026-03-26

**Authors:** Shilpa Yadav, Mansi, Priyanshu, Pratibha Chanana, Pankaj Khanna, Asmita Singh, Leena Khanna

**Affiliations:** a University School of Basic & Applied Sciences, Guru Gobind Singh Indraprastha University Dwarka New Delhi-110078 India leenakhanna@ipu.ac.in; b University School of Chemical Technology, Guru Gobind Singh Indraprastha University Dwarka New Delhi-110078 India; c Synthesis & In-Silico Drug Design Laboratory, Department of Chemistry, Acharya Narendra Dev College, University of Delhi Kalkaji New Delhi-110019 India

## Abstract

Correction for “Synthesis and multifaceted exploration of dibenzoxepinones: *in vitro* antimicrobial and ct-DNA binding, DFT/TD-DFT, molecular docking and simulation studies” by Shilpa Yadav *et al.*, *RSC Adv.*, 2025, **15**, 18089–18107, https://doi.org/10.1039/D5RA01068C.

The authors regret that details related to funding were omitted within the acknowledgements. The corrected acknowledgements are as shown here.

The Royal Society of Chemistry apologises for these errors and any consequent inconvenience to authors and readers.

