# Supplementary material for: Synthesis and multifaceted exploration of dibenzoxepinones: in vitro antimicrobial and ct-DNA binding, DFT/TD-DFT, molecular docking and simulation studies
Source: RSC Adv. 2025 May 30;15(23):18089–107. doi: 10.1039/d5ra01068c (PMC12123684; doi:10.1039/d5ra01068c)
Supplement: RA-015-D5RA01068C-s001 [file RA-015-D5RA01068C-s001.pdf]

Supplementary Information (SI) for RSC Advances.
This journal is © The Royal Society of Chemistry 2025

Supplementary Information

Synthesis and multifaceted exploration of dibenzoxepinones: in-vitro antimicrobial and ct-DNA binding, DFT/TD-DFT, molecular docking and simulation studies

Shilpa Yadav[1], Mansi[1], Priyanshu[2], Pratibha Chanana[2], Pankaj Khanna[3], Asmita Singh[1] & Leena Khanna[1]*

[1]*University School of Basic & Applied Sciences, Guru Gobind Singh Indraprastha University, Dwarka, New Delhi-110078, India.*

[2]*University School of Chemical Technology, Guru Gobind Singh Indraprastha University, Dwarka, New Delhi-110078, India*

[3]*Department of Chemistry, Acharya Narendra Dev College, University of Delhi, Kalkaji, New Delhi-110019, India.*

Contents

1. Proton NMR Spectra
2. Carbon NMR Spectra
3. High-Resolution Mass Spectra
4. Binding energy of all compounds with antibacterial, antifungal, and ct-DNA activity.
5. Molecular Docking Images for Antibacterial Activity
6. Molecular Docking Images for Antifungal Activity
7. Molecular Docking Images for ct-DNA Activity
8. Physicochemical Properties Values
9. DFT coordinates for UV-vis
10. DFT coordinates for FTIR
11. DFT coordinates for ESP
12. In-vitro antibacterial photos

1. **Proton NMR Spectra**

3

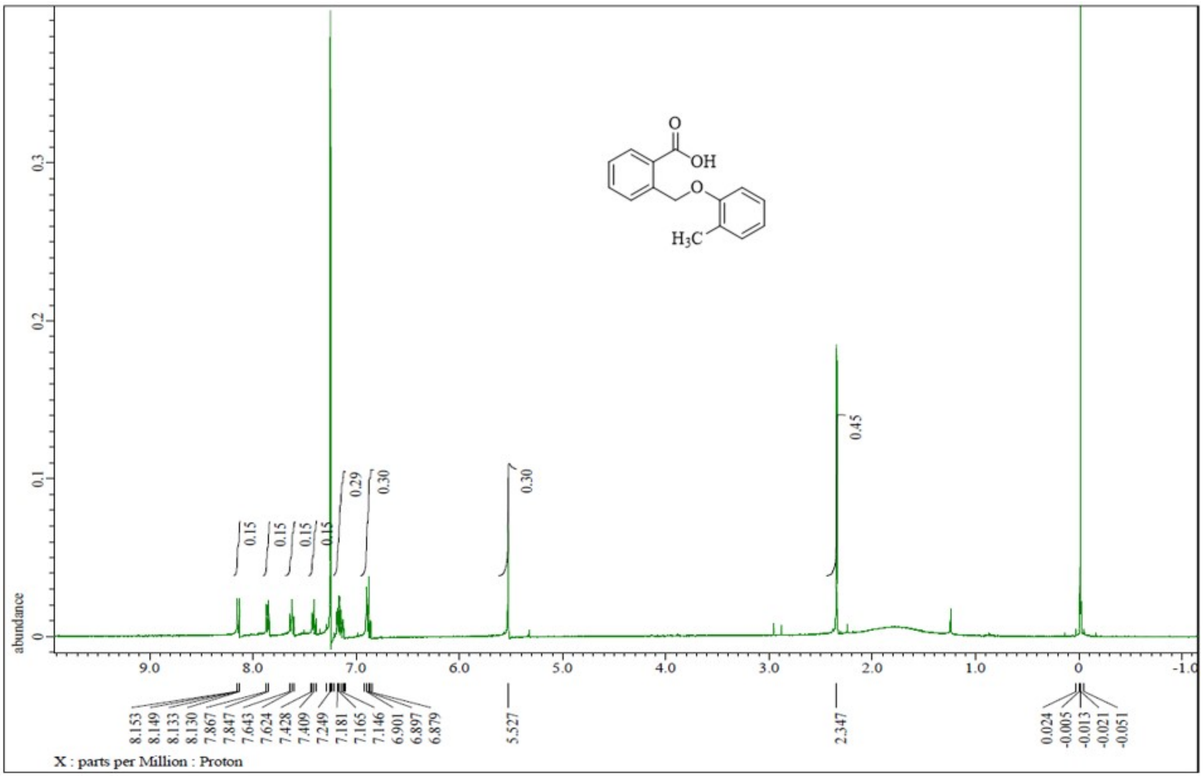

4

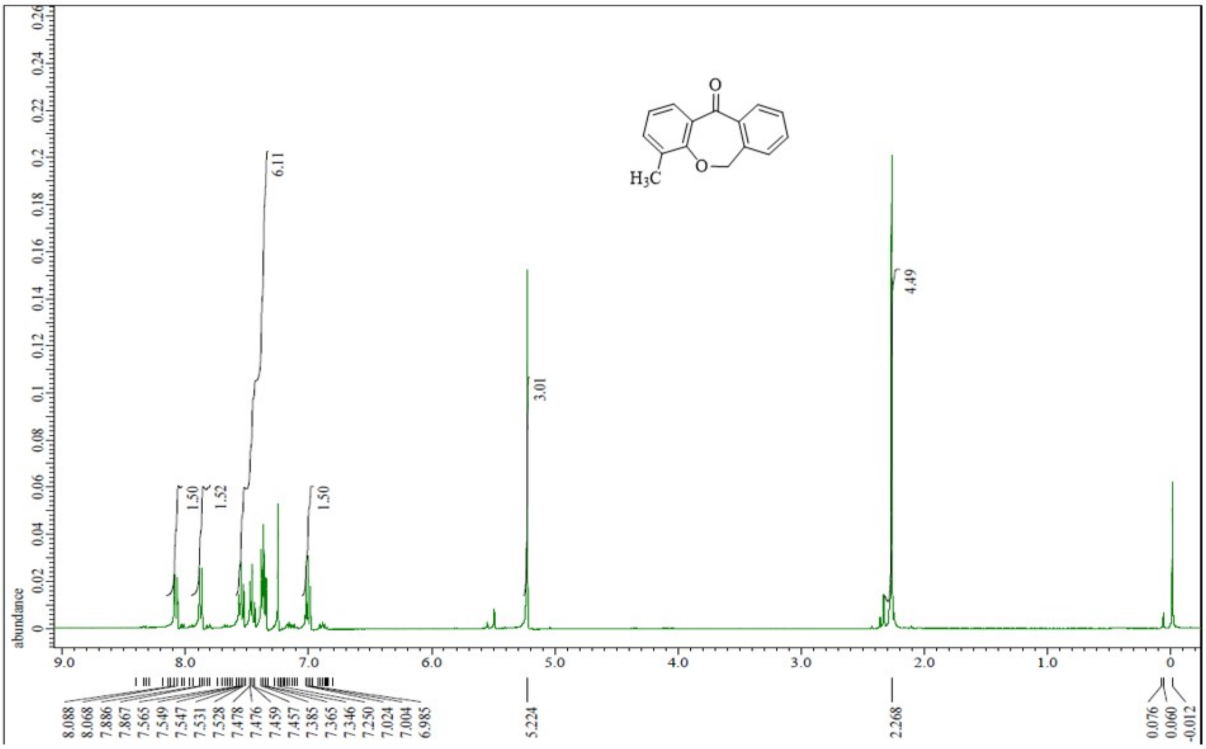

5

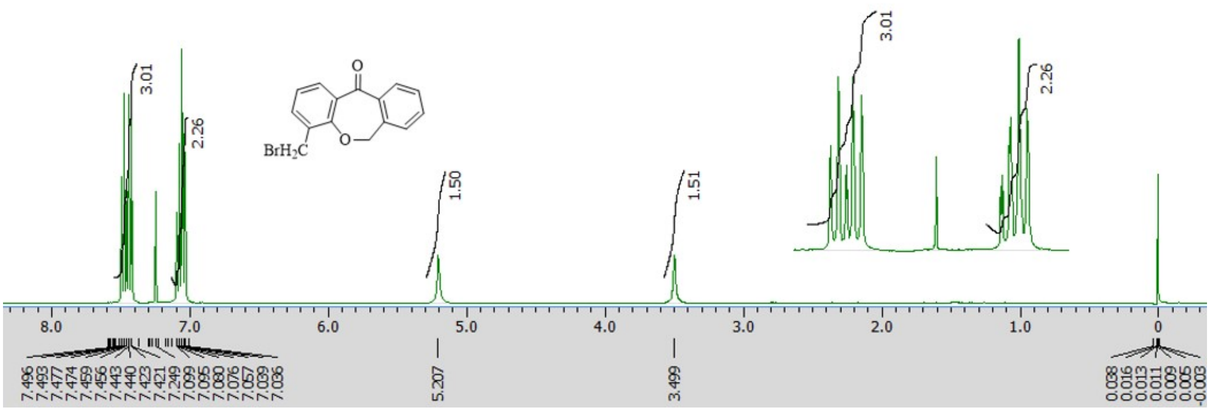
BrH2C
3.01
2.26
1.50
1.51
8.0
7.0
6.0
5.0
4.0
3.0
2.0
1.0
0
7.496
7.493
7.477
7.474
7.459
7.456
7.443
7.440
7.423
7.421
7.249
7.099
7.095
7.080
7.076
7.057
7.039
7.036
5.207
3.499
0.038
0.016
0.013
0.011
0.009
0.005
-0.003

7a

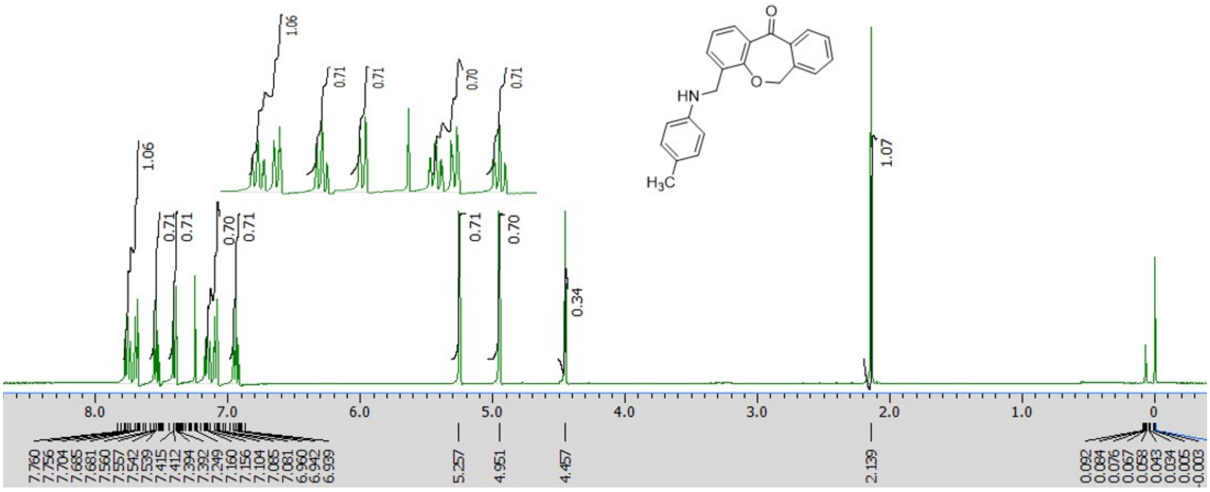
HN
H3C
1.06
0.71
0.71
0.70
0.71
0.71
0.70
0.34
1.07
8.0
7.0
6.0
5.0
4.0
3.0
2.0
1.0
0
7.760
7.756
7.704
7.685
7.681
7.560
7.557
7.542
7.539
7.415
7.412
7.394
7.392
7.249
7.160
7.156
7.104
7.085
7.081
6.960
6.942
6.939
5.257
4.951
4.457
2.139
0.092
0.084
0.076
0.067
0.058
0.043
0.034
0.005
-0.003

7b

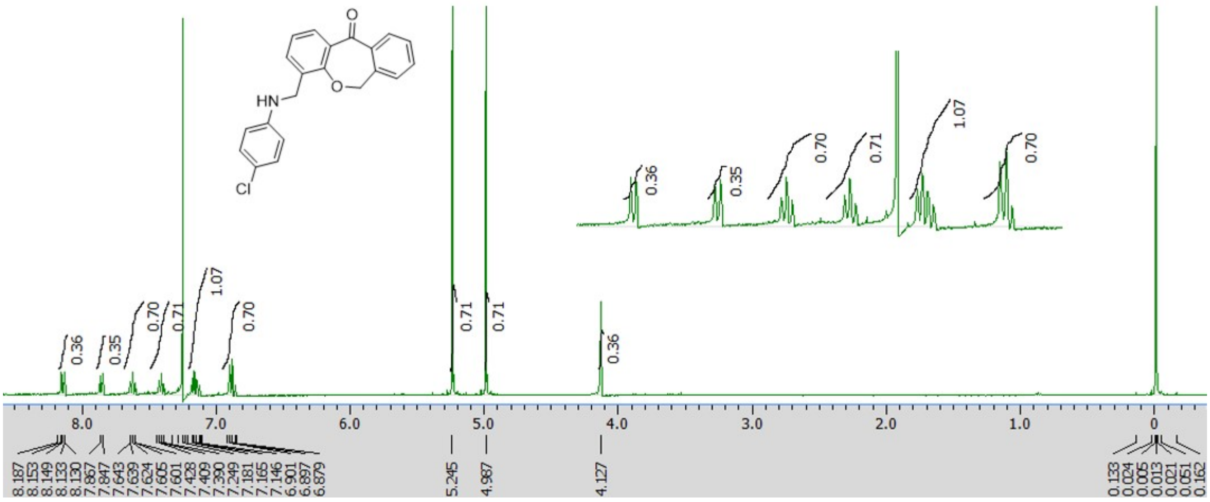
HN
Cl
0.36
0.35
0.70
0.71
1.07
0.70
0.71
0.71
0.36
8.0
7.0
6.0
5.0
4.0
3.0
2.0
1.0
0
8.187
8.153
8.149
8.133
8.130
7.867
7.847
7.643
7.639
7.624
7.605
7.601
7.428
7.409
7.390
7.249
7.181
7.165
7.146
6.901
6.897
6.879
5.245
4.987
4.127
0.133
0.024
0.005
0.013
0.021
0.051
0.162

7c

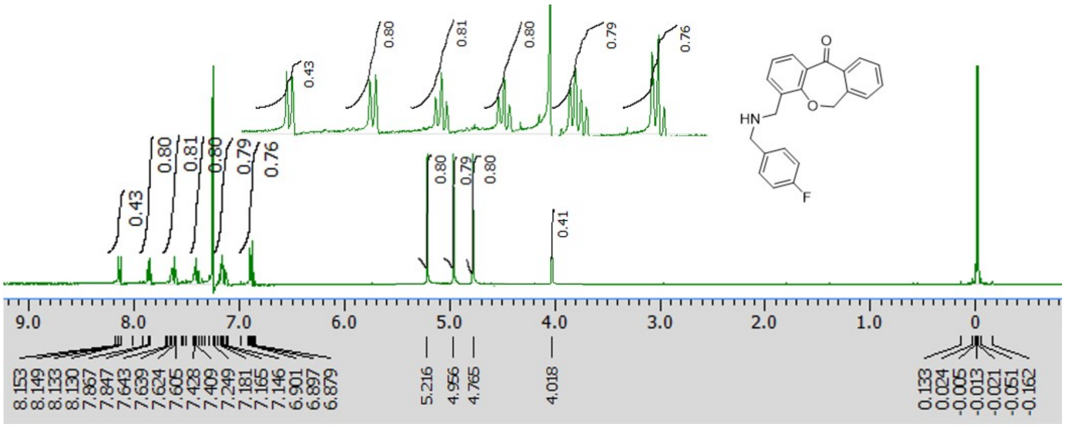
0.43
0.80
0.81
0.80
0.79
0.76
0.80
0.79
0.80
0.41
HN
O
F
9.0
8.0
7.0
6.0
5.0
4.0
3.0
2.0
1.0
0
8.153 8.149 8.133 8.130 7.867 7.847 7.643 7.639 7.624 7.605 7.428 7.409 7.249 7.181 7.165 7.146 6.901 6.897 6.879
5.216
4.956
4.765
4.018
0.133 0.024 -0.005 -0.013 -0.021 -0.051 -0.162

7d

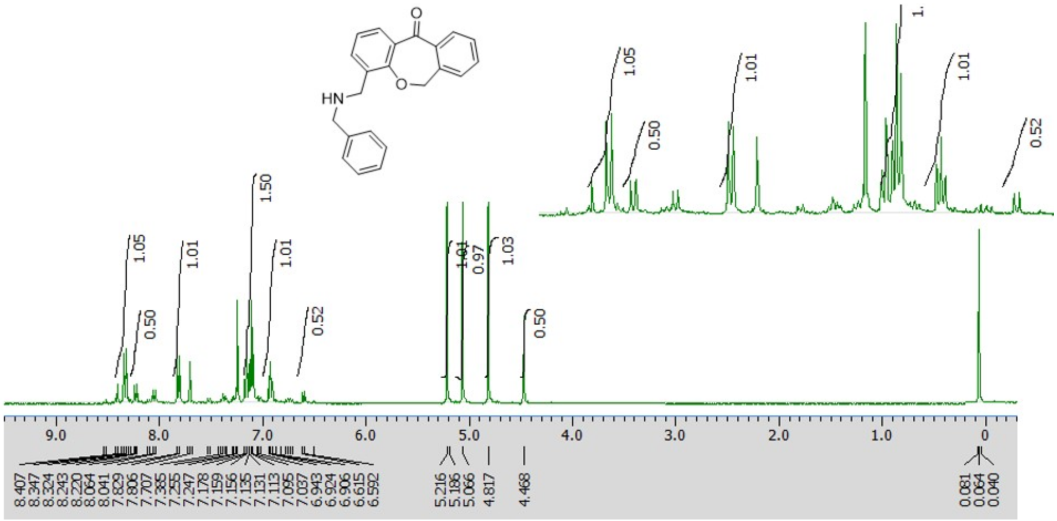
HN
O
1.05
0.50
1.01
1.50
1.01
0.52
1.01
0.97
1.03
0.50
9.0
8.0
7.0
6.0
5.0
4.0
3.0
2.0
1.0
0
8.407 8.347 8.324 8.243 8.220 8.064 8.041 7.829 7.806 7.707 7.385 7.255 7.247 7.178 7.159 7.156 7.135 7.131 7.113 7.095 7.037 6.943 6.924 6.906 6.615 6.592
5.216 5.186 5.066 4.817
4.468
0.081 0.064 0.040

7e

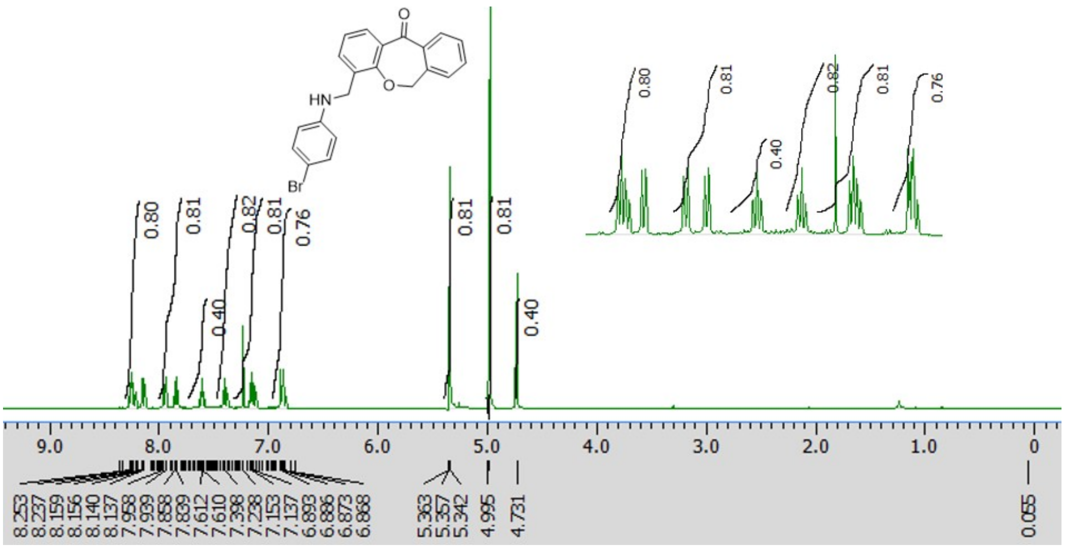
O
HN
O
Br
0.80
0.81
0.40
0.82
0.81
0.76
0.81
0.81
0.40
9.0
8.0
7.0
6.0
5.0
4.0
3.0
2.0
1.0
0
8.253 8.237 8.159 8.156 8.140 8.137 7.958 7.939 7.858 7.839 7.612 7.610 7.398 7.238 7.153 7.137 6.893 6.886 6.873 6.868
5.363 5.357 5.342
4.995
4.731
0.055

7f

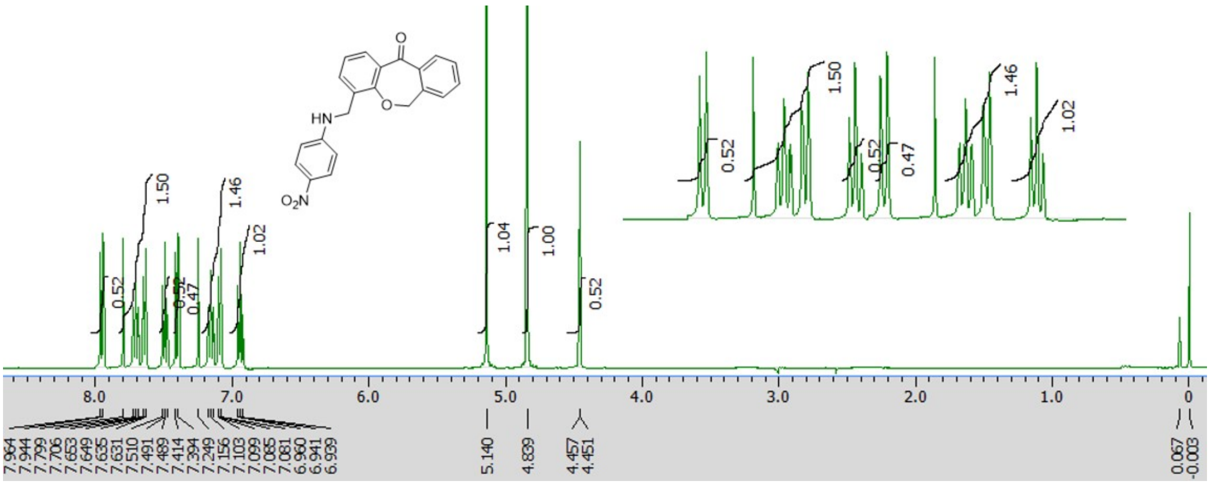
O
HN
O
O_2N
1.50
0.52
1.46
1.02
0.47
1.04
1.00
0.52
8.0
7.0
6.0
5.0
4.0
3.0
2.0
1.0
0
7.964 7.944 7.799 7.706 7.653 7.649 7.635 7.631 7.510 7.491 7.489 7.414 7.394 7.249 7.156 7.103 7.099 7.085 7.081 6.960 6.941 6.939
5.140
4.839
4.457 4.451
0.067 -0.003

7g

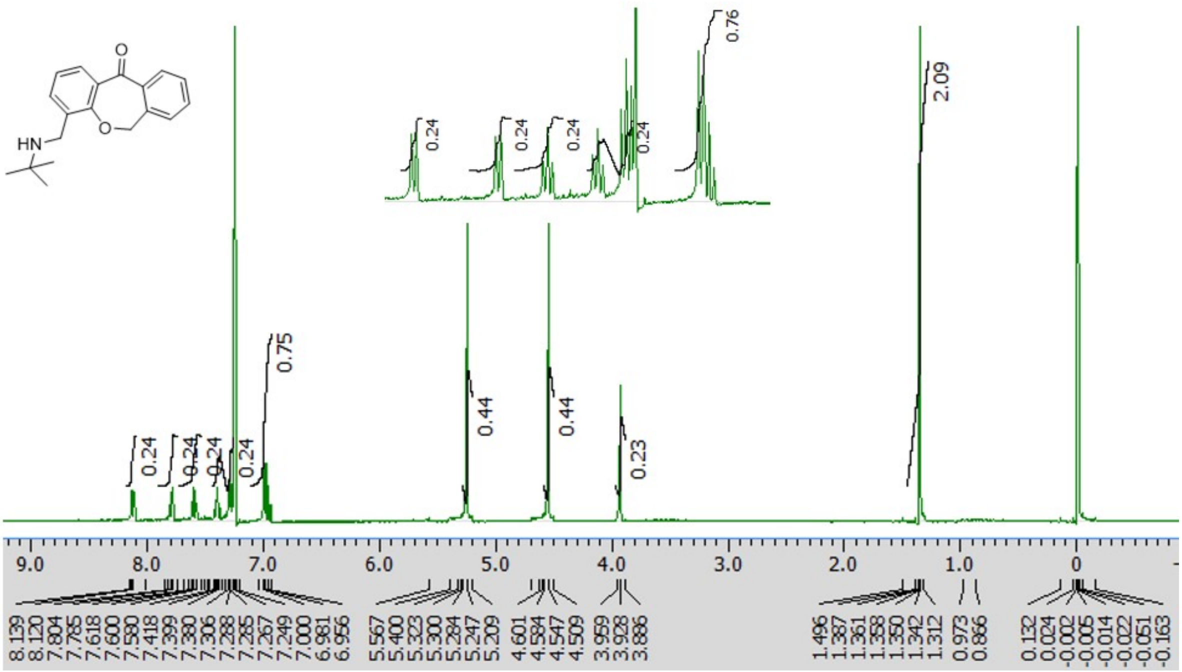
HN
O
O
0.76
2.09
0.24
0.24
0.24
0.24
0.75
0.44
0.44
0.23
9.0 8.0 7.0 6.0 5.0 4.0 3.0 2.0 1.0 0
8.139 8.120 7.804 7.785 7.618 7.600 7.580 7.418 7.399 7.380 7.306 7.288 7.285 7.267 7.249 7.000 6.981 6.956
5.567 5.400 5.323 5.300 5.284 5.247 5.209
4.601 4.584 4.547 4.509
3.959 3.928 3.886
1.496 1.387 1.361 1.358 1.350 1.342 1.312
0.973 0.866
0.132 0.024 -0.002 -0.005 -0.014 -0.022 -0.051 -0.163

7h

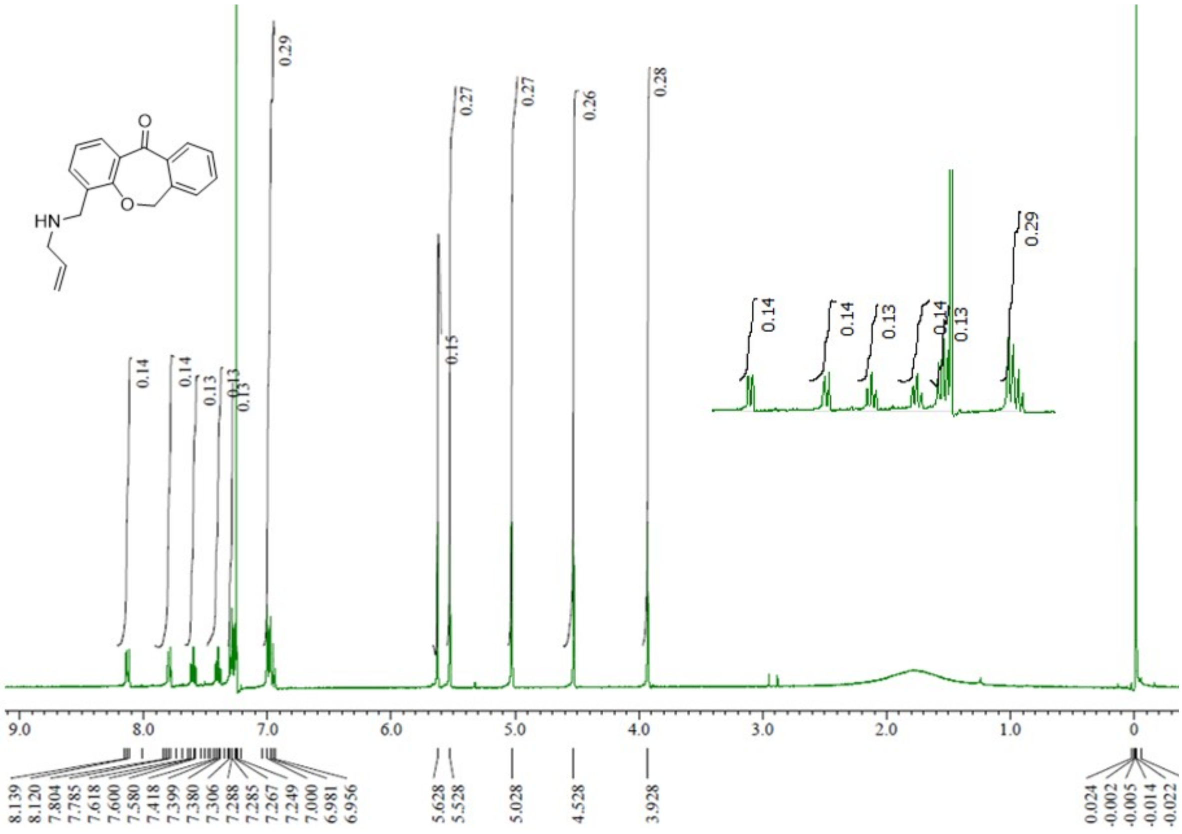
HN
O
O
0.29
0.27
0.27
0.26
0.28
0.14
0.14
0.13
0.13
0.13
0.15
0.29
9.0 8.0 7.0 6.0 5.0 4.0 3.0 2.0 1.0 0
8.139 8.120 7.804 7.785 7.618 7.600 7.580 7.418 7.399 7.380 7.306 7.288 7.285 7.267 7.249 7.000 6.981 6.956
5.628 5.528
5.028
4.528
3.928
0.024 -0.002 -0.005 -0.014 -0.022

7i

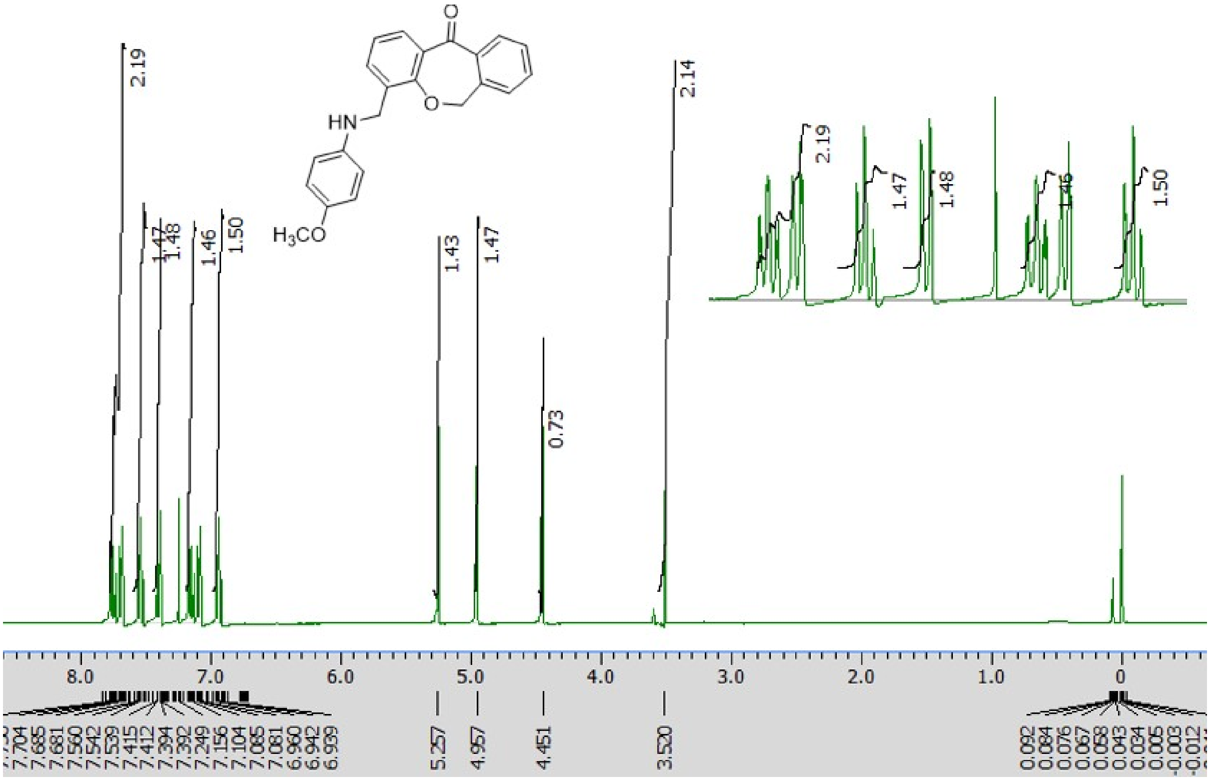

7j

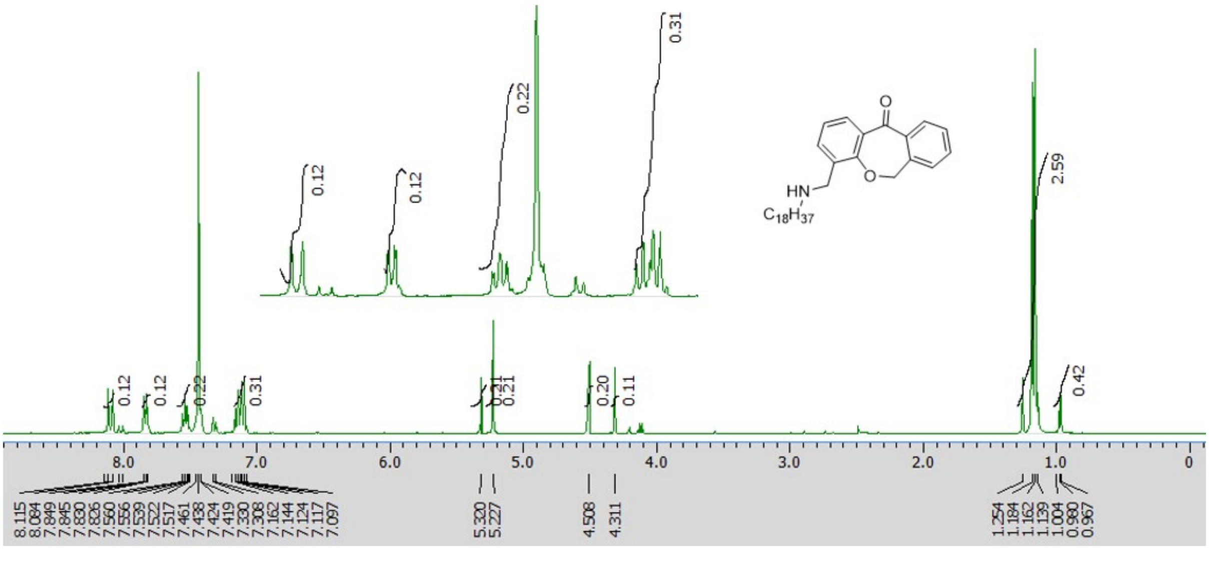

2. Carbon NMR Spectra

4

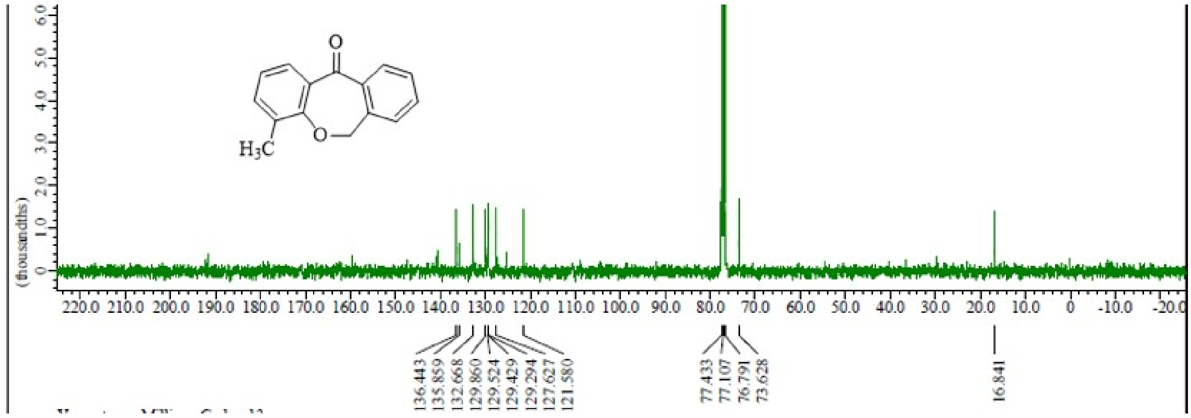

O
H_3C
O
(thousandths)
220.0 210.0 200.0 190.0 180.0 170.0 160.0 150.0 140.0 130.0 120.0 110.0 100.0 90.0 80.0 70.0 60.0 50.0 40.0 30.0 20.0 10.0 0 -10.0 -20.0
136.443
135.859
132.668
129.860
129.524
129.429
129.294
127.627
121.580
77.433
77.107
76.791
73.628
16.841

5

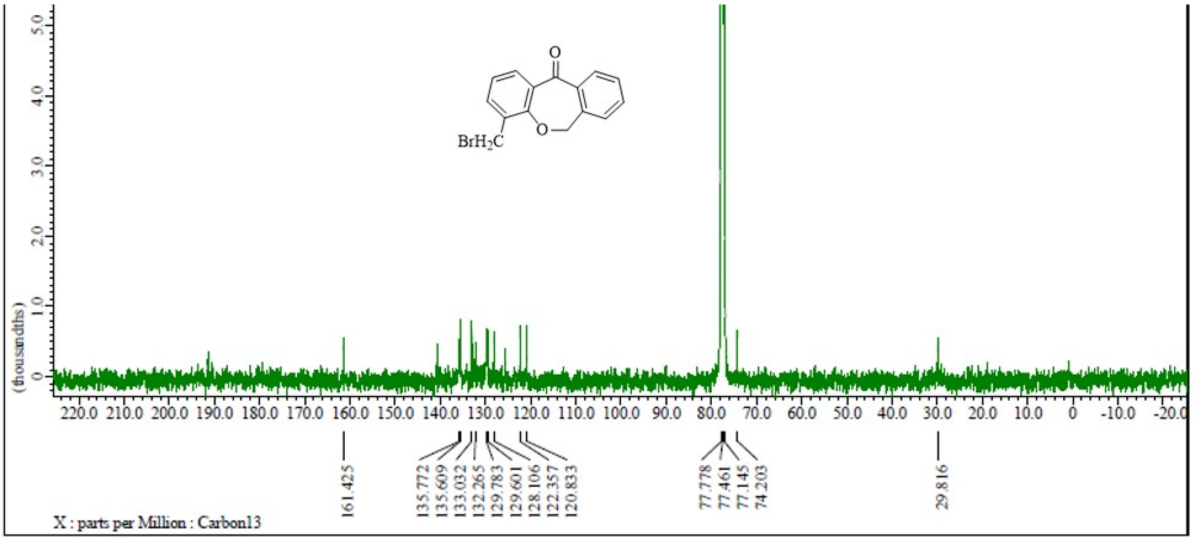

O
BrH_2C
O
(thousandths)
220.0 210.0 200.0 190.0 180.0 170.0 160.0 150.0 140.0 130.0 120.0 110.0 100.0 90.0 80.0 70.0 60.0 50.0 40.0 30.0 20.0 10.0 0 -10.0 -20.0
161.425
135.772
135.609
133.032
132.265
129.783
129.601
128.106
122.357
120.833
77.778
77.461
77.145
74.203
29.816
X : parts per Million : Carbon13

7a

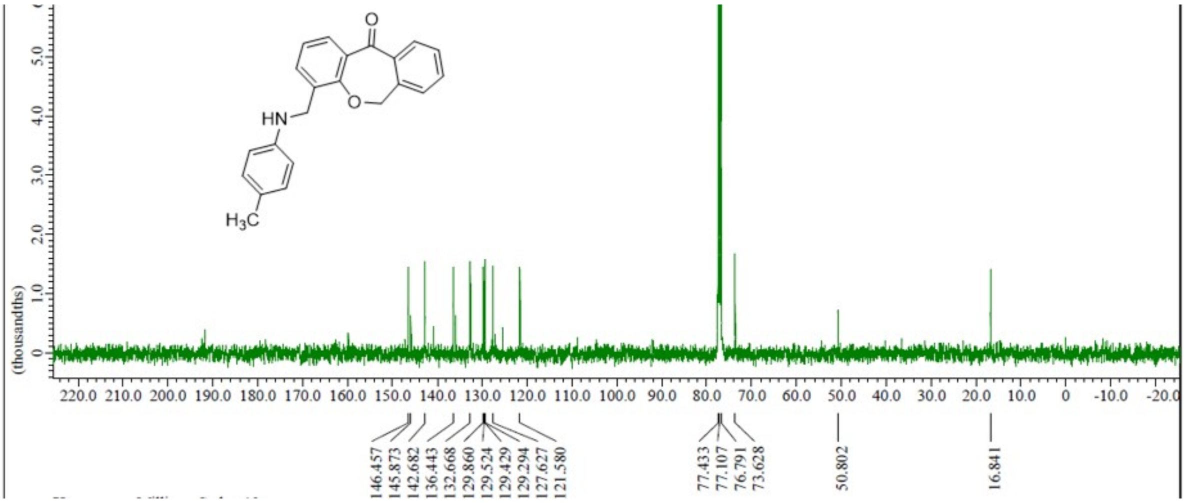

O
HN
O
H_3C
(thousandths)
220.0 210.0 200.0 190.0 180.0 170.0 160.0 150.0 140.0 130.0 120.0 110.0 100.0 90.0 80.0 70.0 60.0 50.0 40.0 30.0 20.0 10.0 0 -10.0 -20.0
146.457
145.873
142.682
136.443
132.668
129.860
129.524
129.429
129.294
127.627
121.580
77.433
77.107
76.791
73.628
50.802
16.841

7b

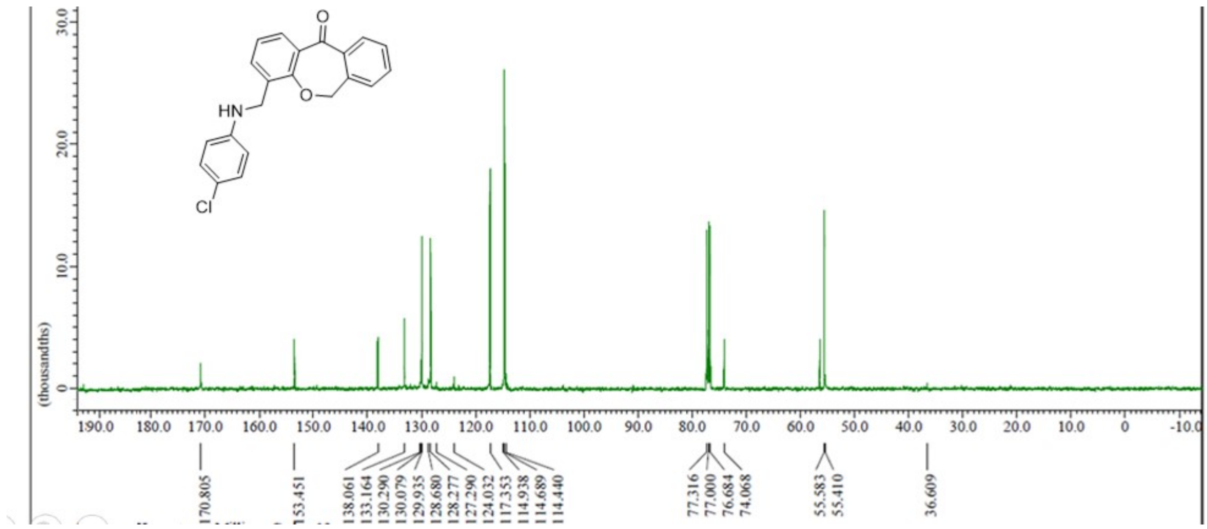
HN
O
Cl
(thousandths)
30.0
20.0
10.0
0
190.0 180.0 170.0 160.0 150.0 140.0 130.0 120.0 110.0 100.0 90.0 80.0 70.0 60.0 50.0 40.0 30.0 20.0 10.0 0 -10.0
170.805
153.451
138.061
133.164
130.290
130.079
129.935
128.680
128.277
127.290
124.032
117.353
114.938
114.689
114.440
77.316
77.000
76.684
74.068
55.583
55.410
36.609

7c

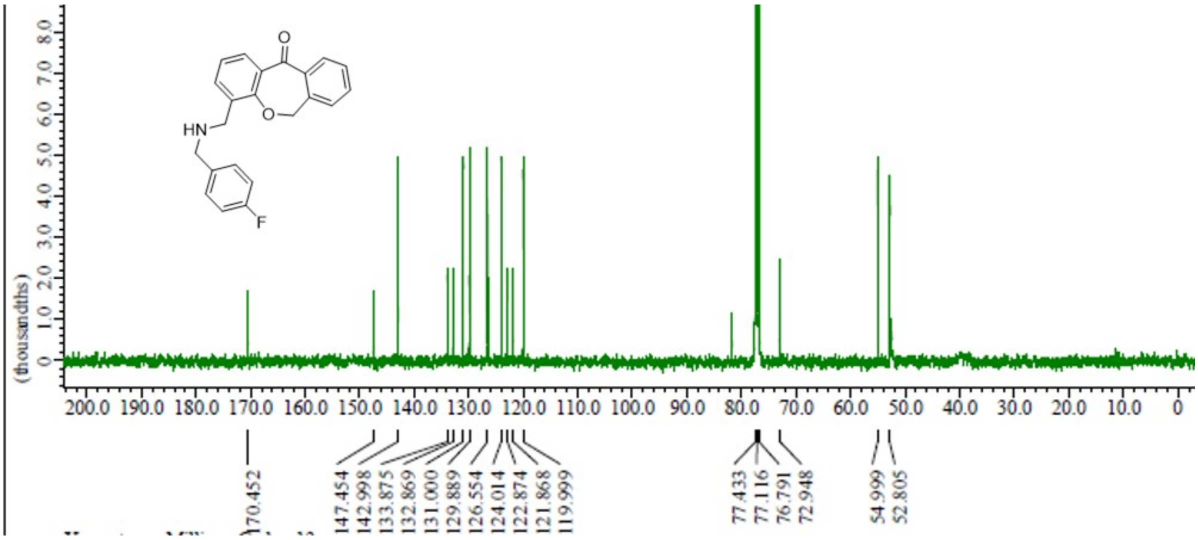
HN
O
F
(thousandths)
8.0
7.0
6.0
5.0
4.0
3.0
2.0
1.0
0
200.0 190.0 180.0 170.0 160.0 150.0 140.0 130.0 120.0 110.0 100.0 90.0 80.0 70.0 60.0 50.0 40.0 30.0 20.0 10.0 0
170.452
147.454
142.998
133.875
132.869
131.000
129.889
126.554
124.014
122.874
121.868
119.999
77.433
77.116
76.791
72.948
54.999
52.805

7d

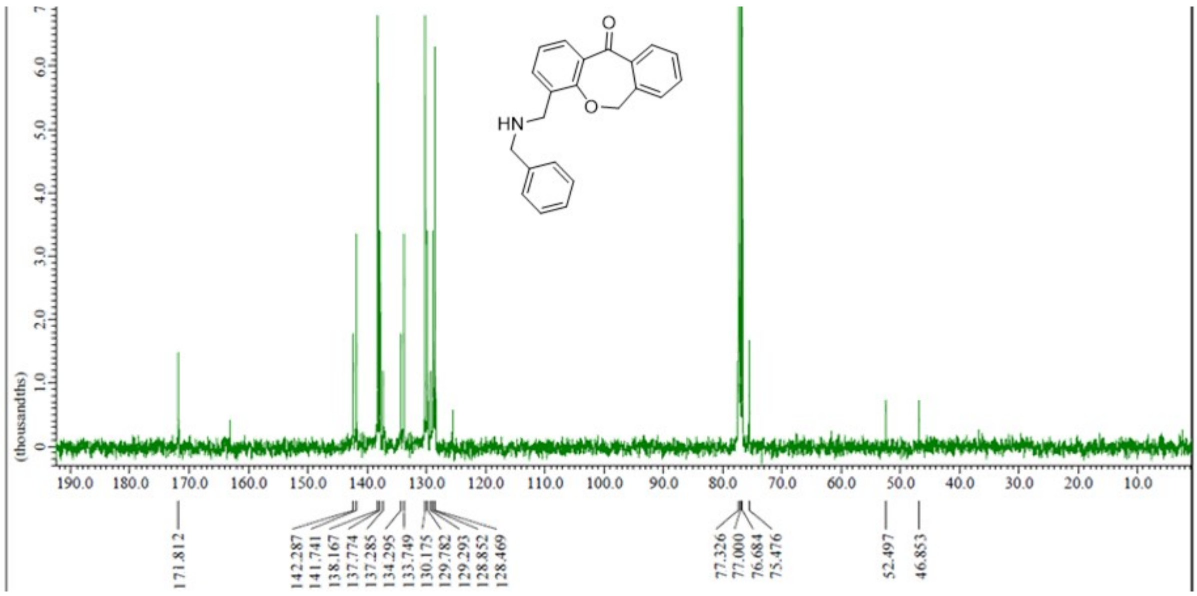

7e

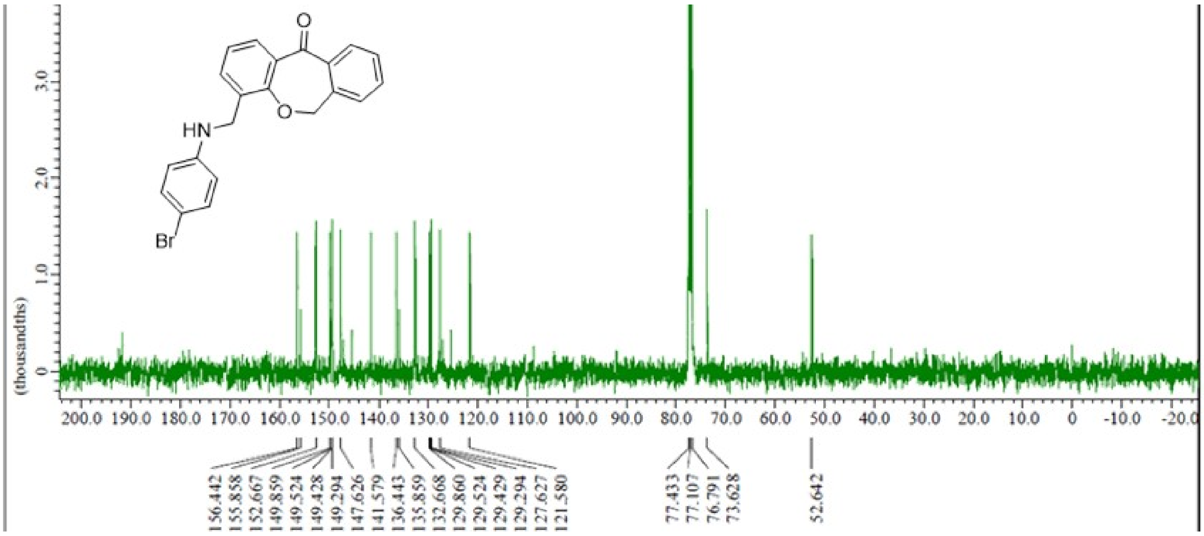

7f

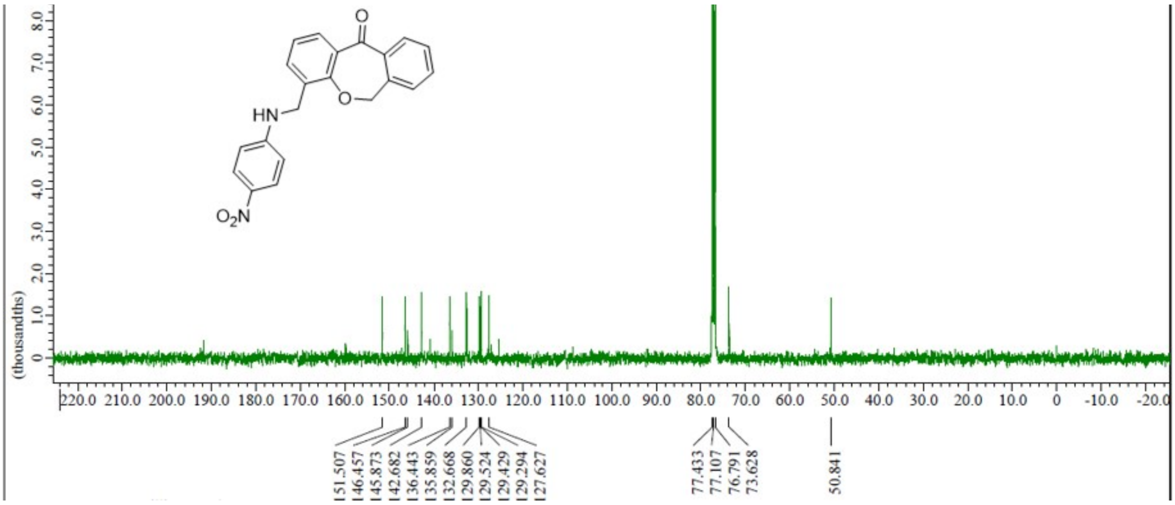

7g

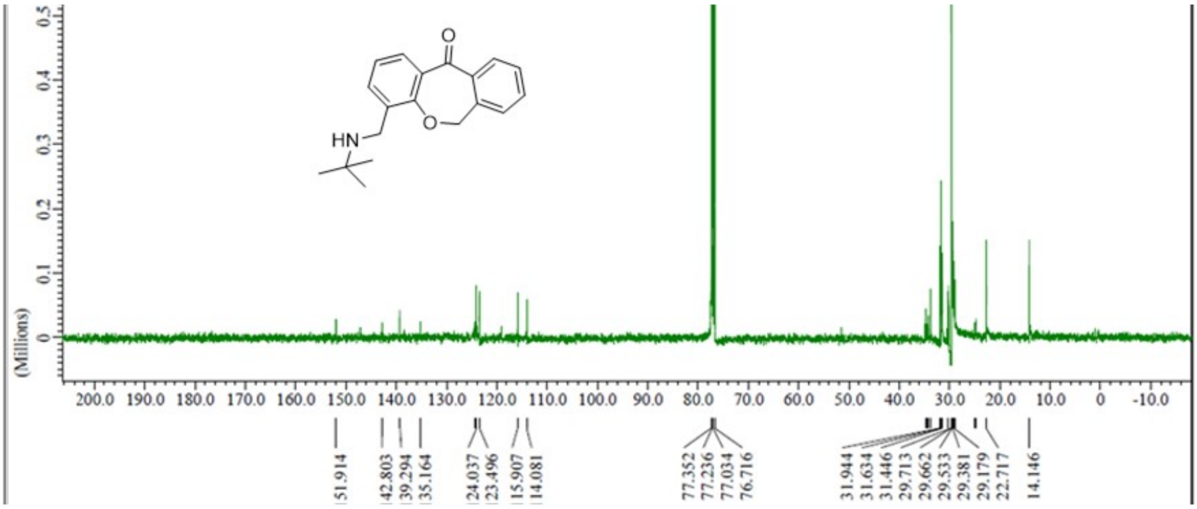
O
HN
O
(Millions)
0 0.1 0.2 0.3 0.4 0.5
200.0 190.0 180.0 170.0 160.0 150.0 140.0 130.0 120.0 110.0 100.0 90.0 80.0 70.0 60.0 50.0 40.0 30.0 20.0 10.0 0 -10.0
151.914
142.803
139.294
135.164
124.037
123.496
115.907
114.081
77.352
77.236
77.034
76.716
31.944
31.634
31.446
29.713
29.662
29.533
29.381
29.179
22.717
14.146

7h

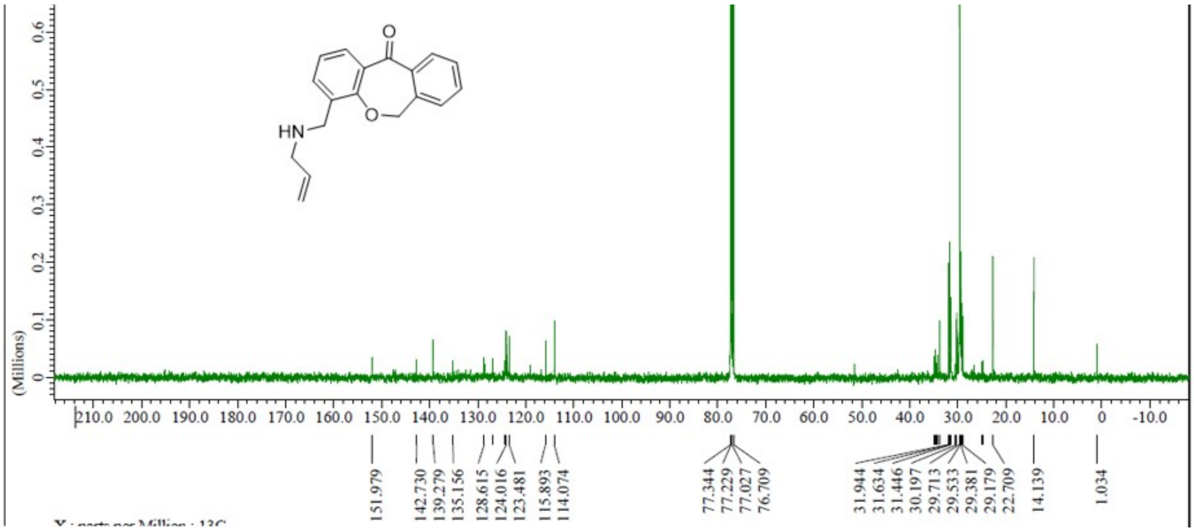
O
HN
O
(Millions)
0 0.1 0.2 0.3 0.4 0.5 0.6
210.0 200.0 190.0 180.0 170.0 160.0 150.0 140.0 130.0 120.0 110.0 100.0 90.0 80.0 70.0 60.0 50.0 40.0 30.0 20.0 10.0 0 -10.0
151.979
142.730
139.279
135.156
128.615
124.016
123.481
115.893
114.074
77.344
77.229
77.027
76.709
31.944
31.634
31.446
30.197
29.713
29.533
29.381
29.179
22.709
14.139
1.034

7i

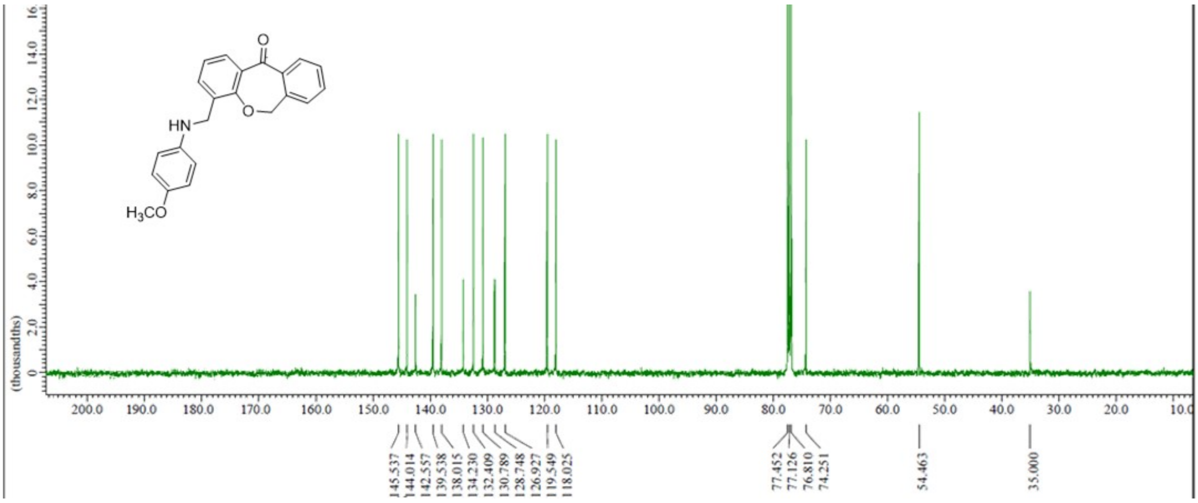

7j

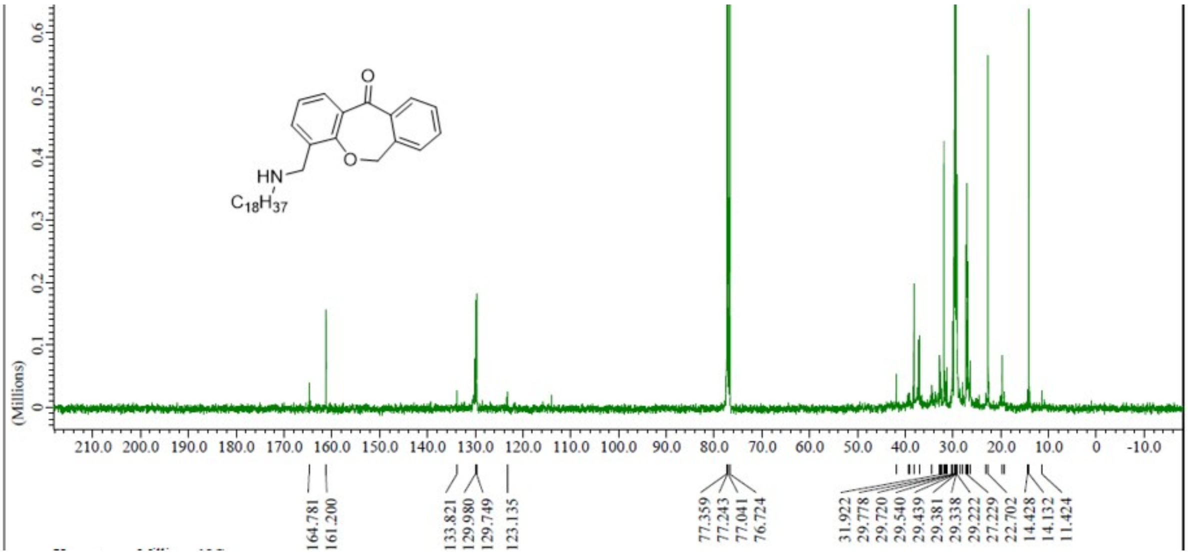

3. High-Resolution Mass Spectra

5

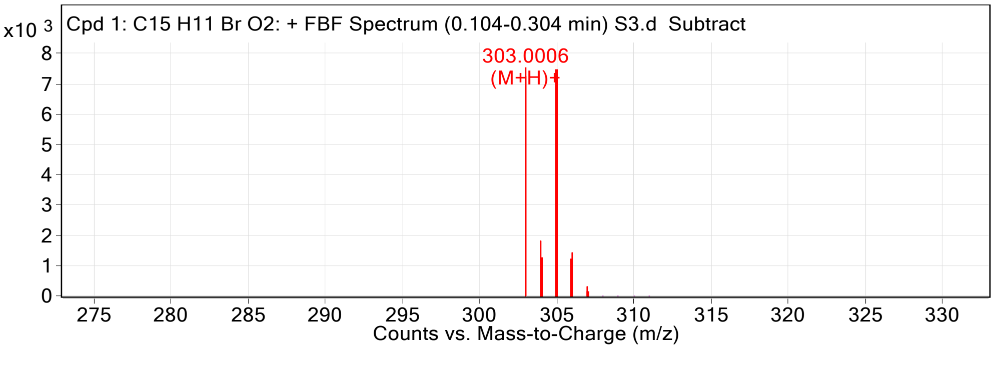

7a

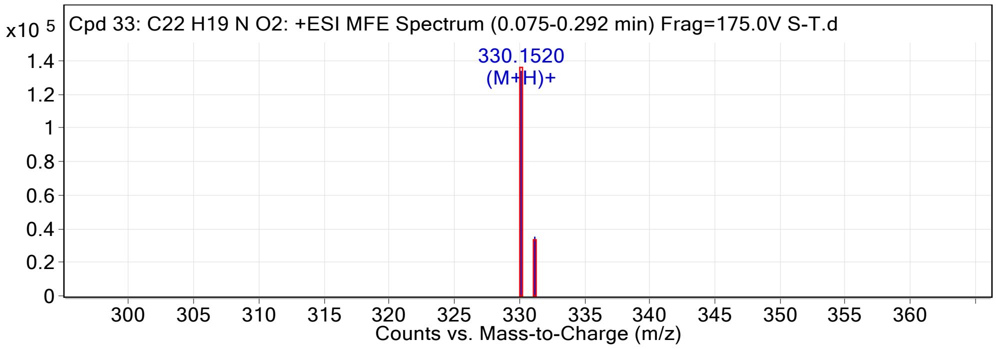

x10 5
Cpd 33: C22 H19 N O2: +ESI MFE Spectrum (0.075-0.292 min) Frag=175.0V S-T.d
330.1520
(M+H)+
1.4
1.2
1
0.8
0.6
0.4
0.2
0
300 305 310 315 320 325 330 335 340 345 350 355 360
Counts vs. Mass-to-Charge (m/z)

7b

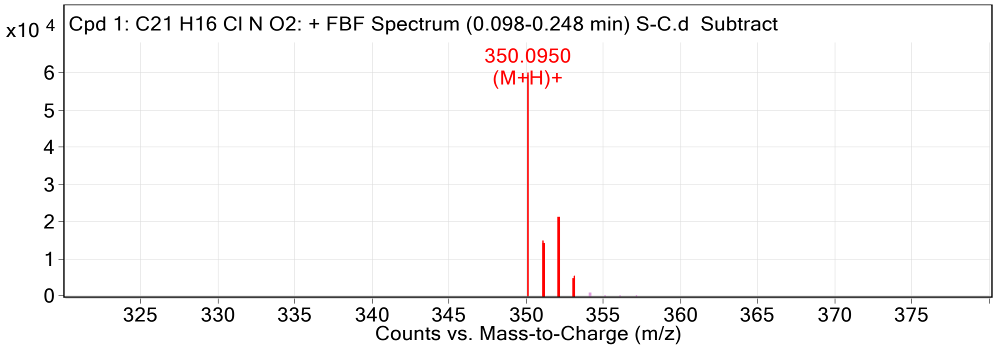

x10 4
Cpd 1: C21 H16 Cl N O2: + FBF Spectrum (0.098-0.248 min) S-C.d Subtract
350.0950
(M+H)+
6
5
4
3
2
1
0
325 330 335 340 345 350 355 360 365 370 375
Counts vs. Mass-to-Charge (m/z)

7c

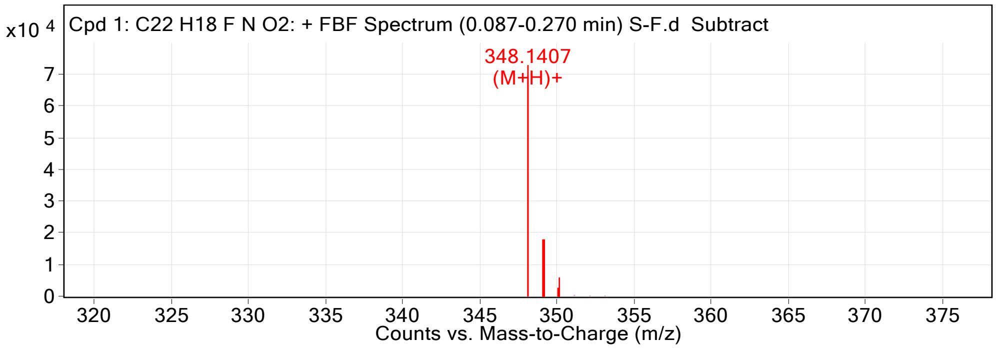

x10 4
Cpd 1: C22 H18 F N O2: + FBF Spectrum (0.087-0.270 min) S-F.d Subtract
348.1407
(M+H)+
7
6
5
4
3
2
1
0
320 325 330 335 340 345 350 355 360 365 370 375
Counts vs. Mass-to-Charge (m/z)

7d

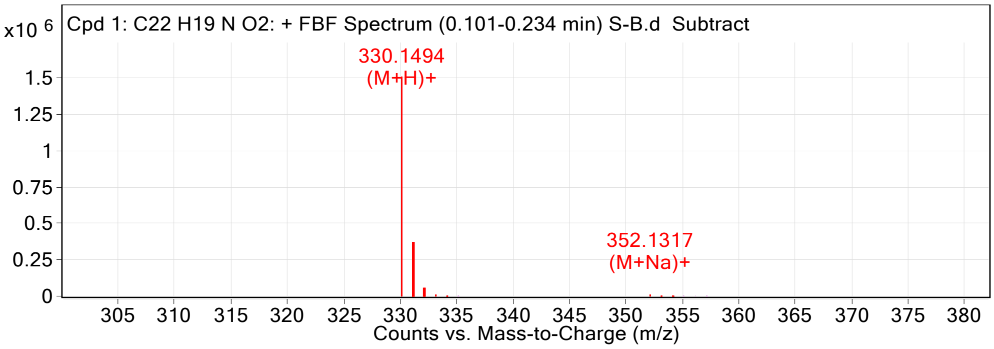
Cpd 1: C22 H19 N O2: + FBF Spectrum (0.101-0.234 min) S-B.d Subtract
x10 6
330.1494
(M+H)+
352.1317
(M+Na)+
Counts vs. Mass-to-Charge (m/z)

7e

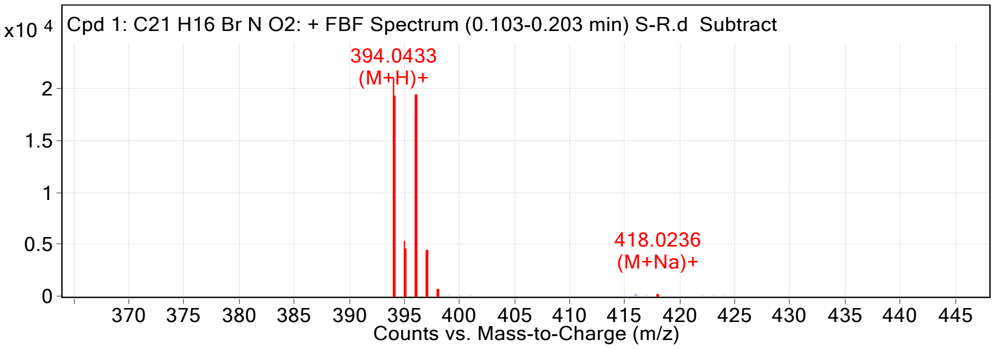
Cpd 1: C21 H16 Br N O2: + FBF Spectrum (0.103-0.203 min) S-R.d Subtract
x10 4
394.0433
(M+H)+
418.0236
(M+Na)+
Counts vs. Mass-to-Charge (m/z)

7f

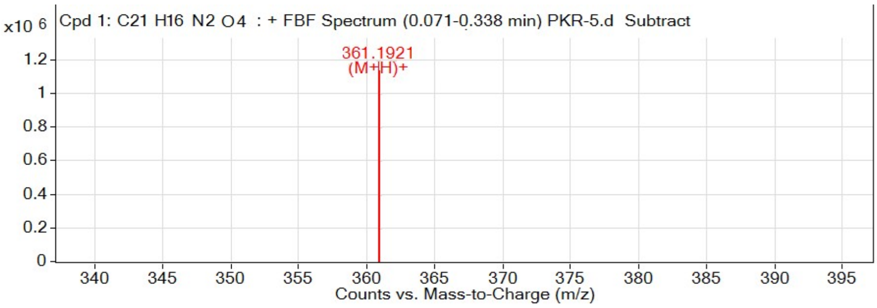
Cpd 1: C21 H16 N2 O4 : + FBF Spectrum (0.071-0.338 min) PKR-5.d Subtract
x10 6
361.1921
(M+H)+
Counts vs. Mass-to-Charge (m/z)

7g

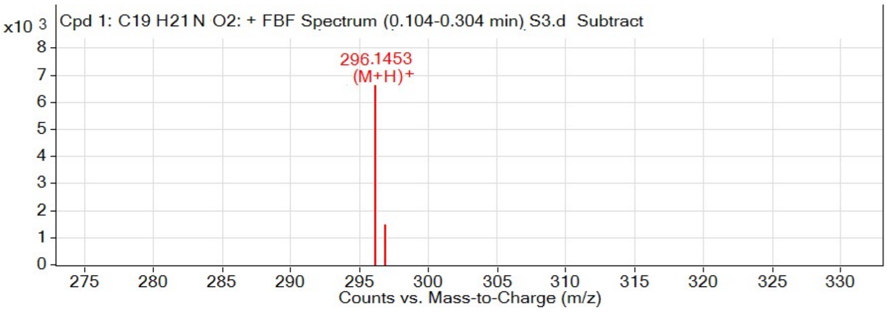
Cpd 1: C19 H21 N O2: + FBF Spectrum (0.104-0.304 min) S3.d Subtract
x10 3
296.1453
(M+H)+
Counts vs. Mass-to-Charge (m/z)

7h

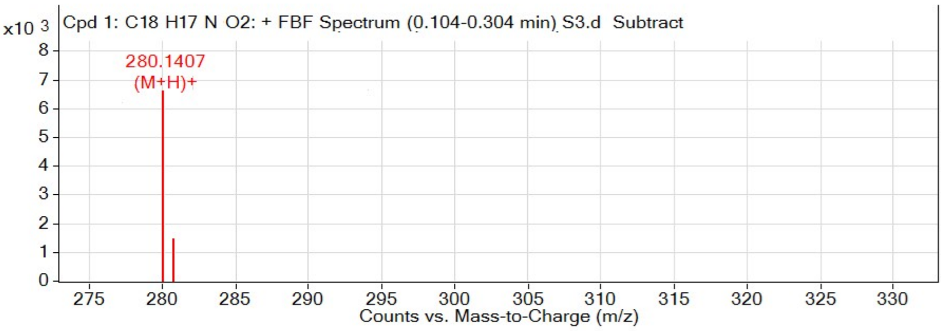

7i

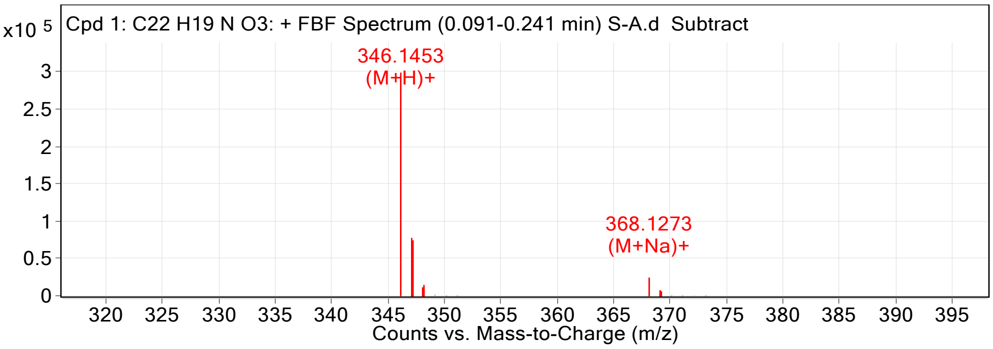

7j

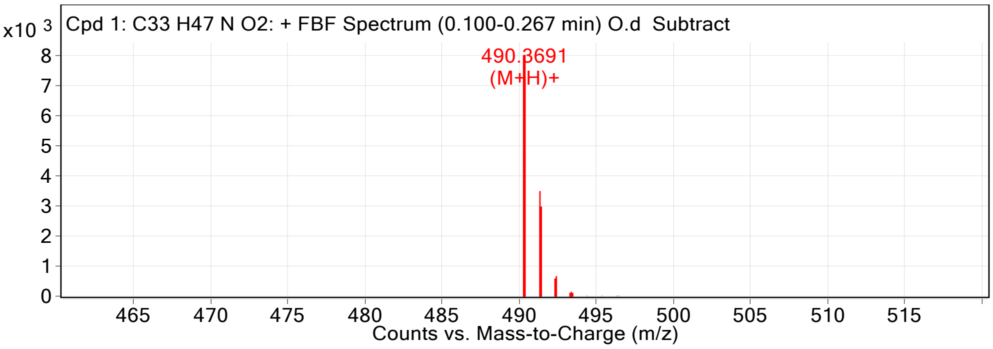

3. **Binding Energy of all compounds with antibacterial, antifungal and ct-DNA activity proteins.**

Table S1. Details of Binding Energy (kcal/mol) and types of interactions between ligands and proteins.

Ligands	Ligand Structure	1KZN	1IYL	1BNA

**7a**	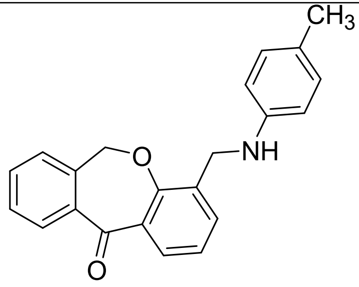	-9.2	-11.0	-8.1
**7b**	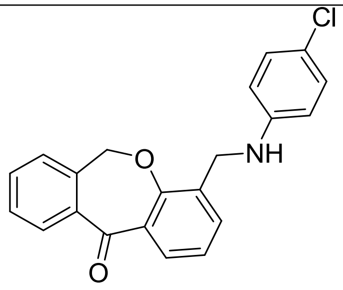	-9.1	-11.0	-7.8
**7c**	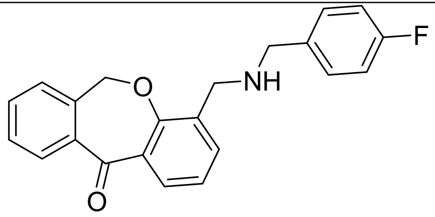	-8.2	-10.9	-6.8
**7d**	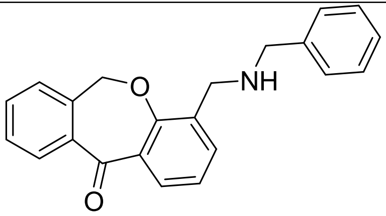	-8.0	-10.9	-7.0
**7e**	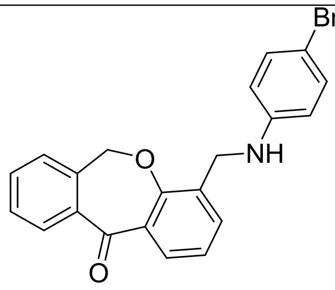	-7.9	-10.6	-8.3
**7f**	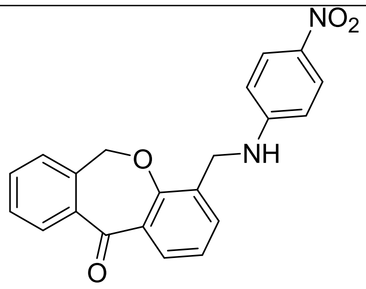	-7.8	-10.2	-7.7

**7g**	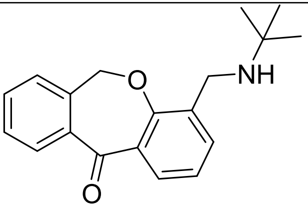	-7.7	-9.3	-6.8
**7h**	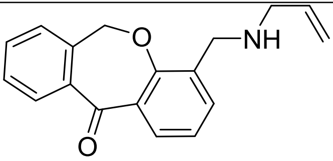	-7.6	-9.2	-7.2
**7i**	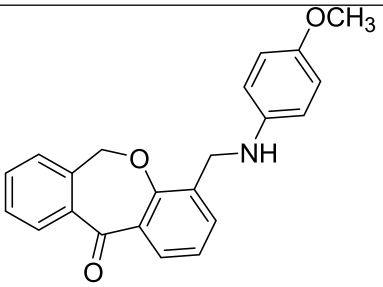	-7.2	-10.2	-7.4
**7j**	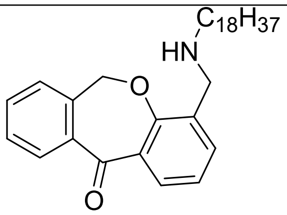	-6.0	-8.5	-5.3

4. Molecular Docking Images for Antibacterial Activity (PDB ID : 1KZN)

7b

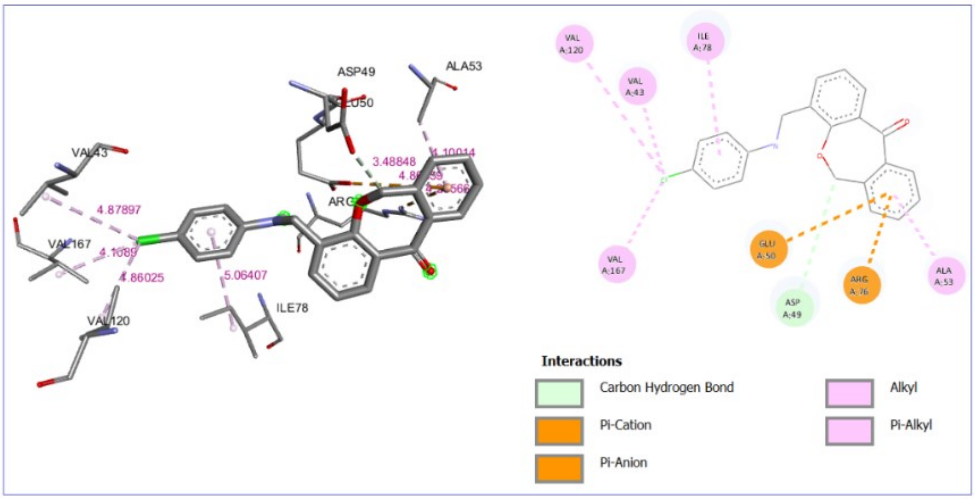

Figure S1. 3D and 2D interaction diagram of compound **7b** with E. Coli bacterial protein.

7c

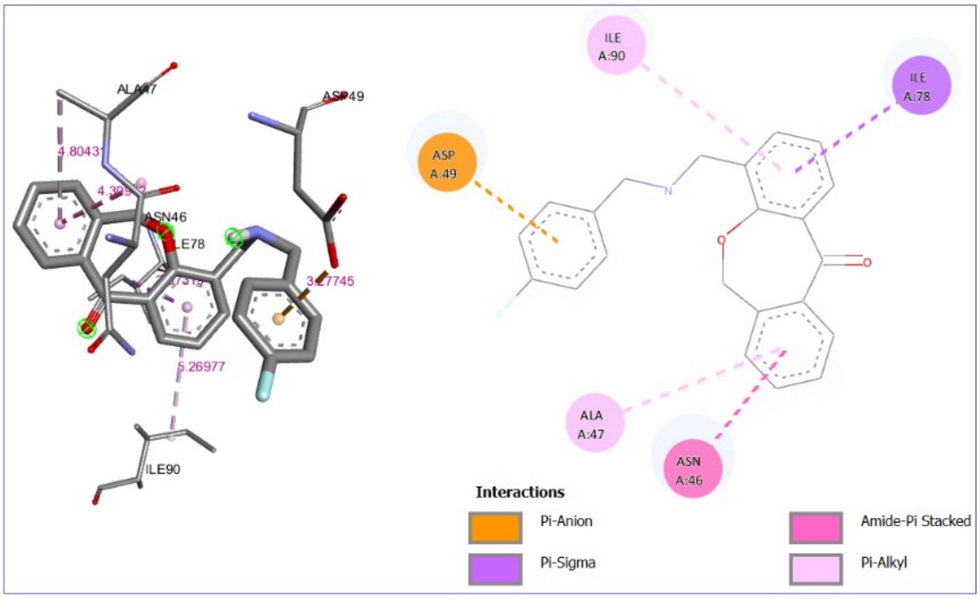

Figure S2. 3D and 2D interaction diagram of compound **7c** with E. Coli bacterial protein.

7d

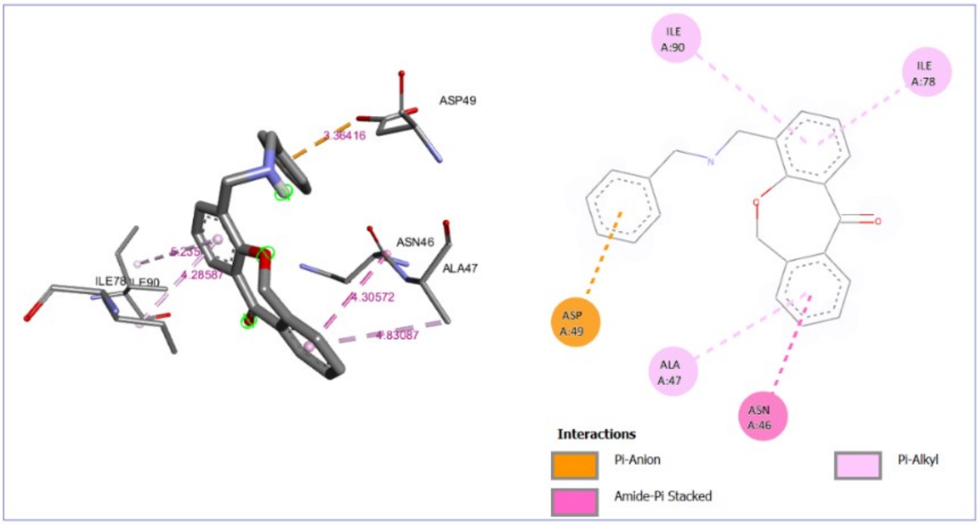

Figure S3. 3D and 2D interaction diagram of compound **7d** with E. Coli bacterial protein.

7e

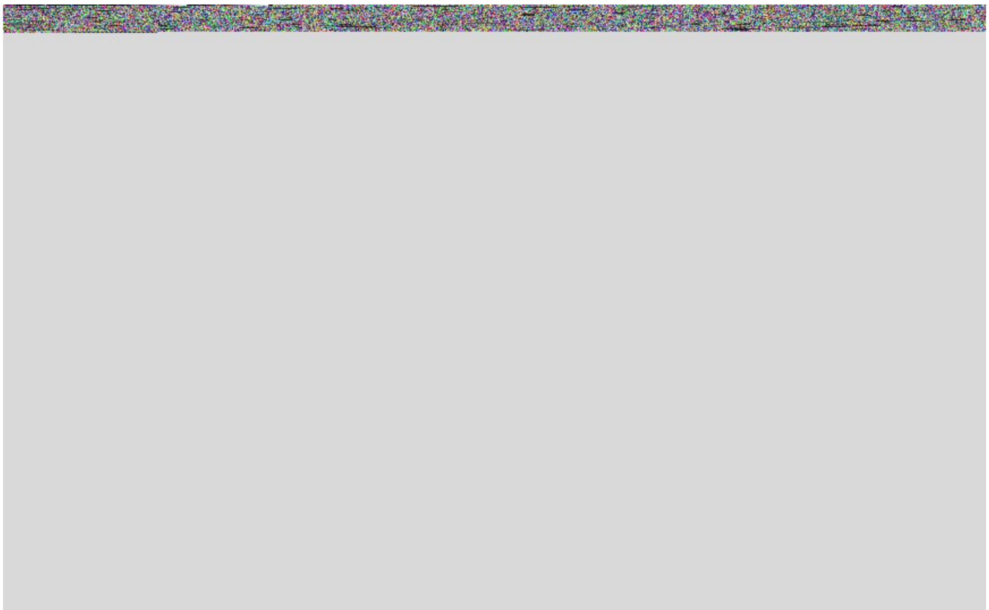

Figure S4. 3D and 2D interaction diagram of compound **7d** with E. Coli bacterial protein.

7f

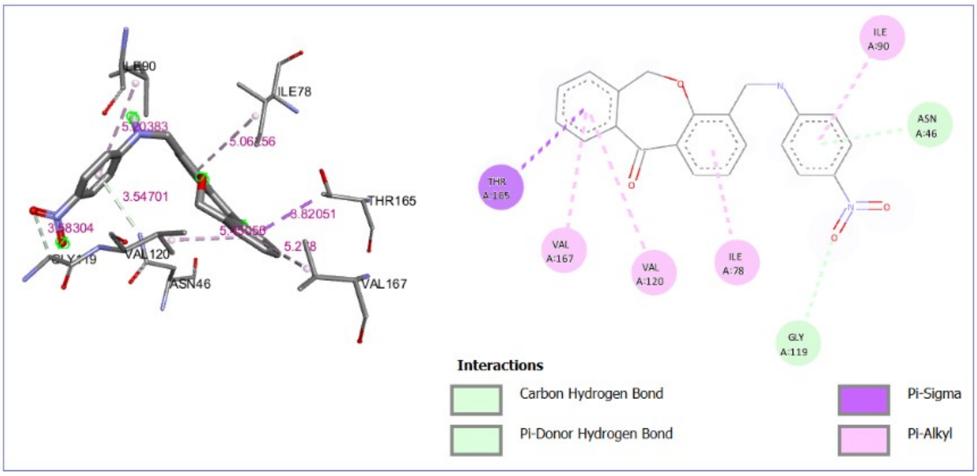

Figure S5. 3D and 2D interaction diagram of compound **7f** with E. Coli bacterial protein.

7g

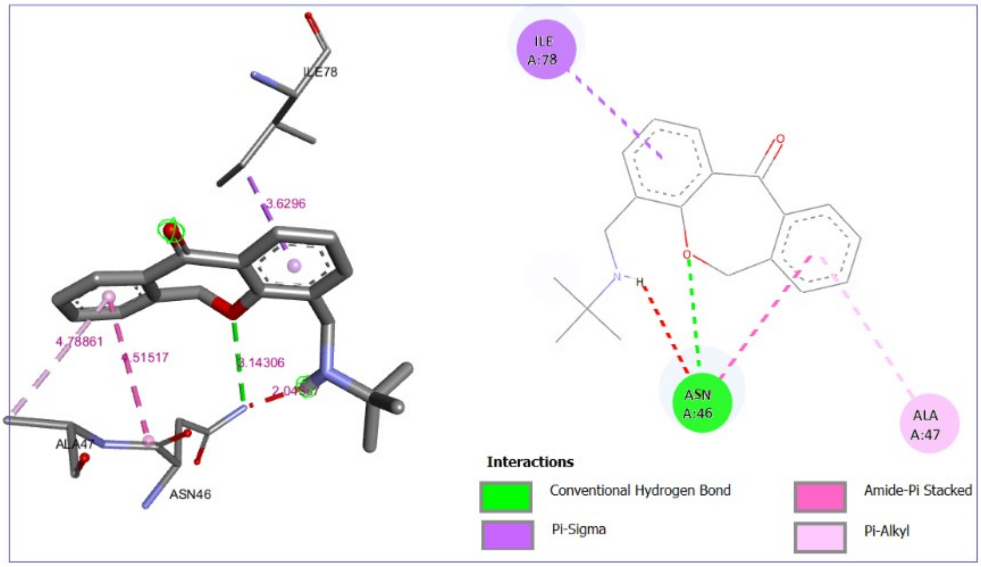

Figure S6. 3D and 2D interaction diagram of compound **7g** with E. Coli bacterial protein.

7h

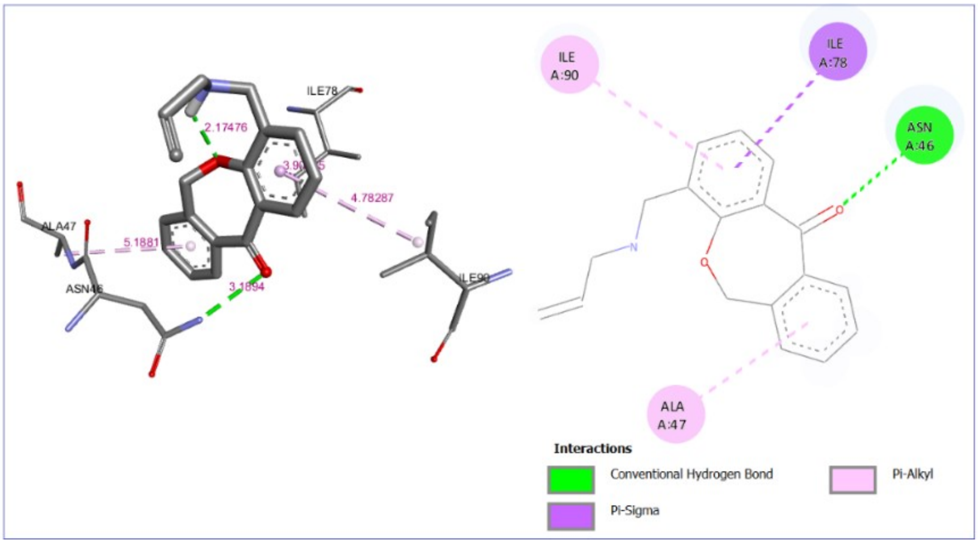

Figure S7. 3D and 2D interaction diagram of compound **7h** with E. Coli bacterial protein.

7i

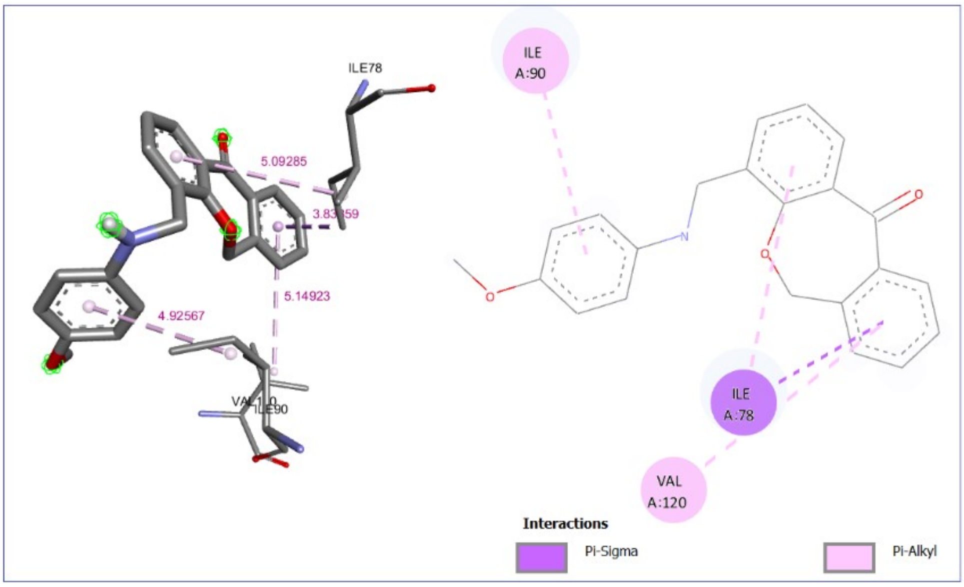

Figure S8. 3D and 2D interaction diagram of compound **7i** with E. Coli bacterial protein.

7j

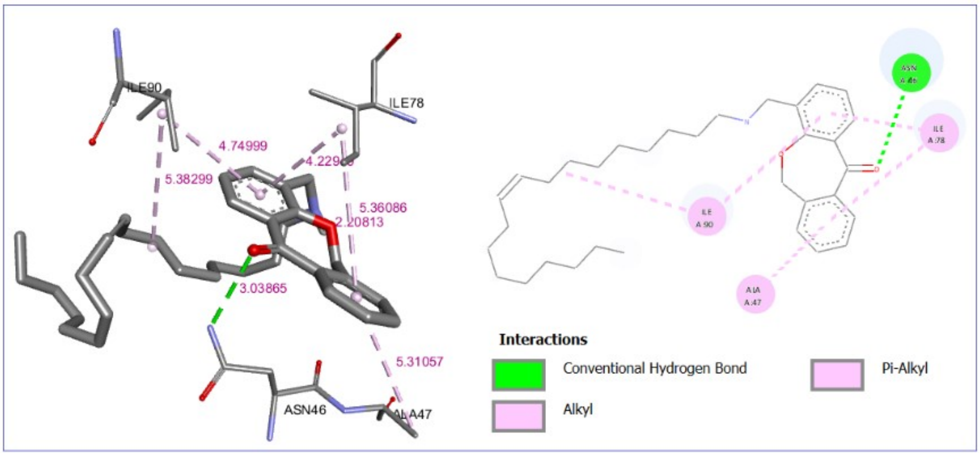

Figure S9. 3D and 2D interaction diagram of compound **7j** with E. Coli bacterial protein.

5. Molecular Docking Images for Antifungal Activity.

7b

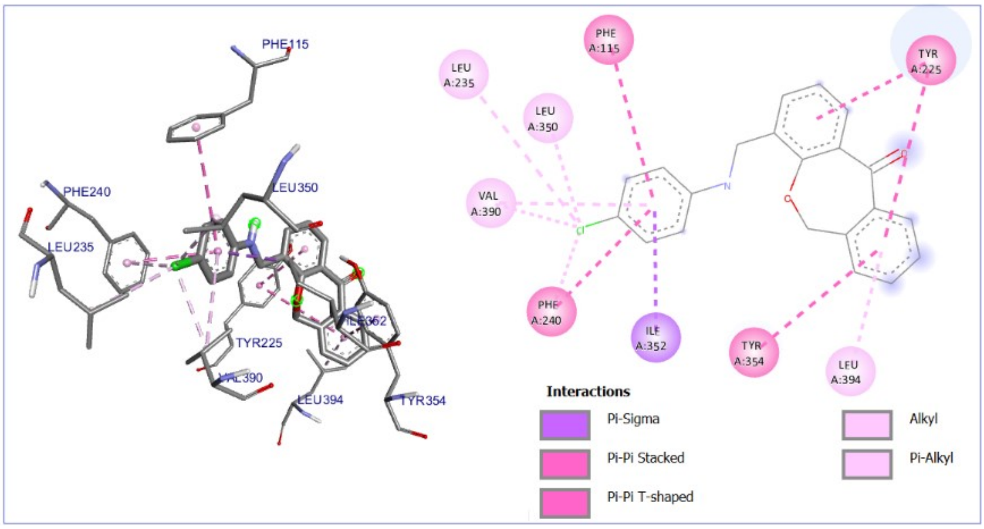

Figure S10. 3D and 2D interaction diagram of compound **7b** with antifungal protein.

7c

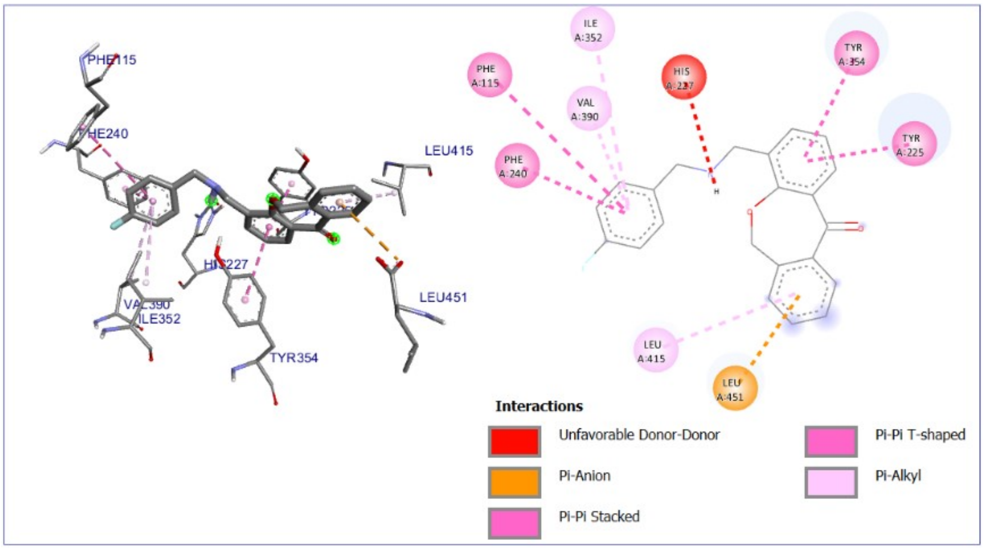

Figure S11. 3D and 2D interaction diagram of compound **7c** with antifungal protein.

7d

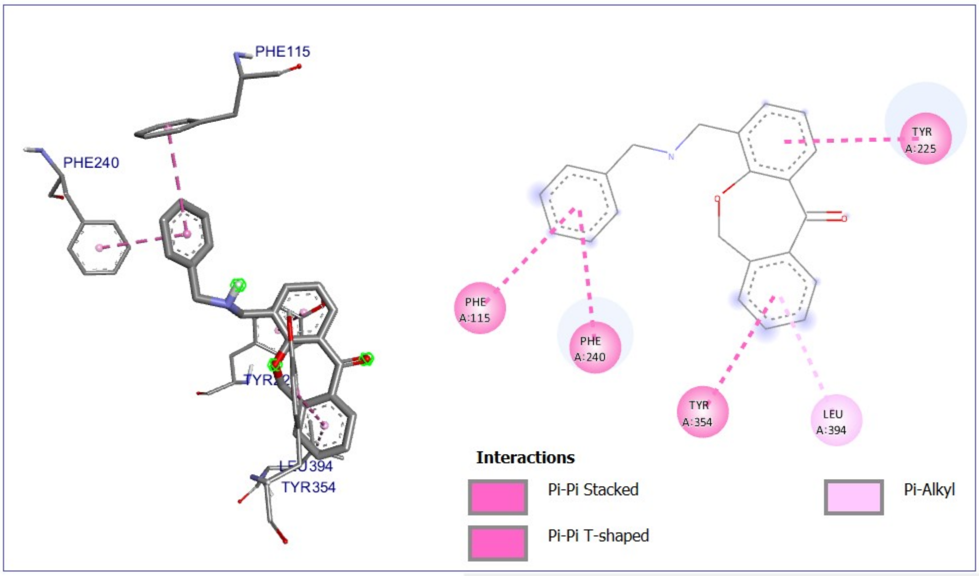

Figure S12. 3D and 2D interaction diagram of compound **7d** with antifungal protein.

7e

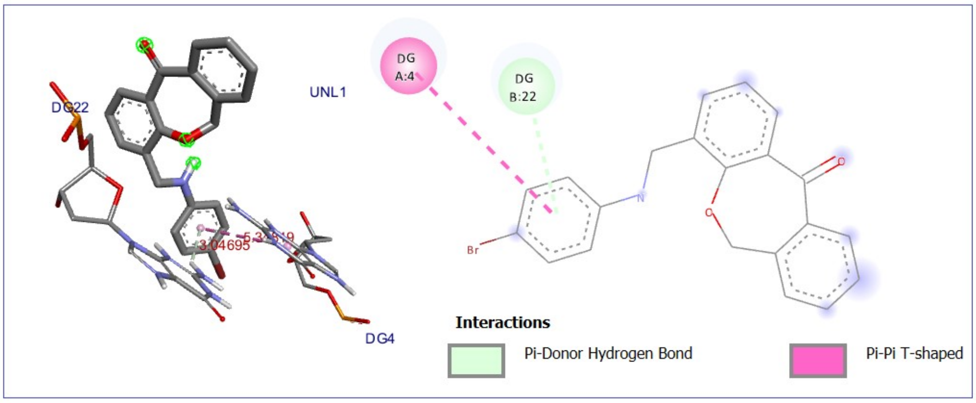

Figure S13. 3D and 2D interaction diagram of compound **7e** with antifungal protein.

7f

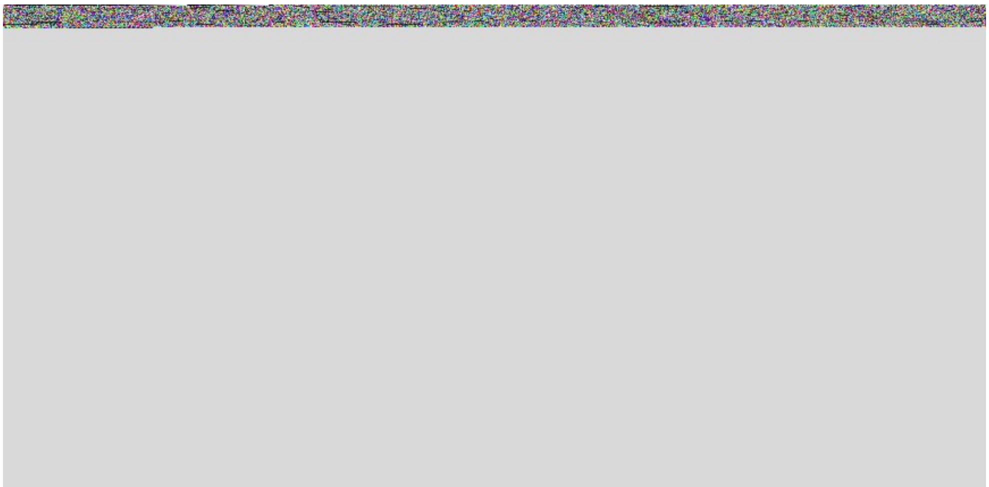

Figure S14. 3D and 2D interaction diagram of compound **7f** with antifungal protein.

7g

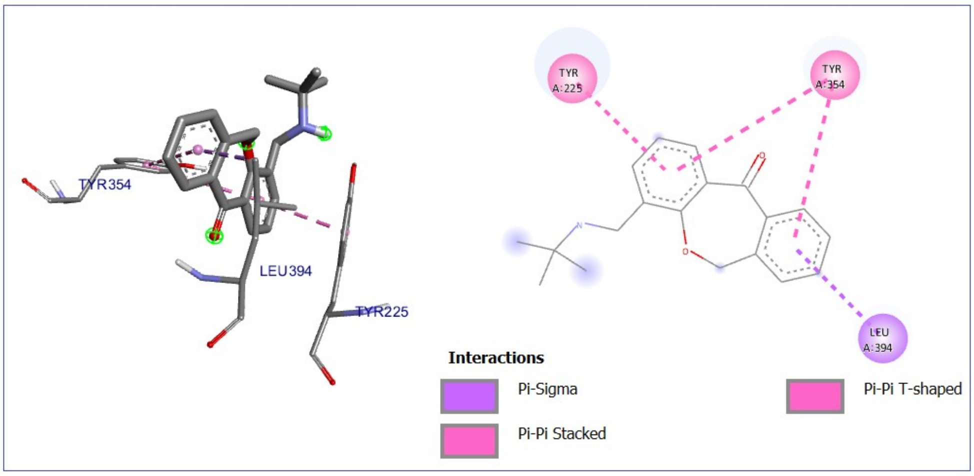

Figure S15. 3D and 2D interaction diagram of compound **7g** with antifungal protein.

7h

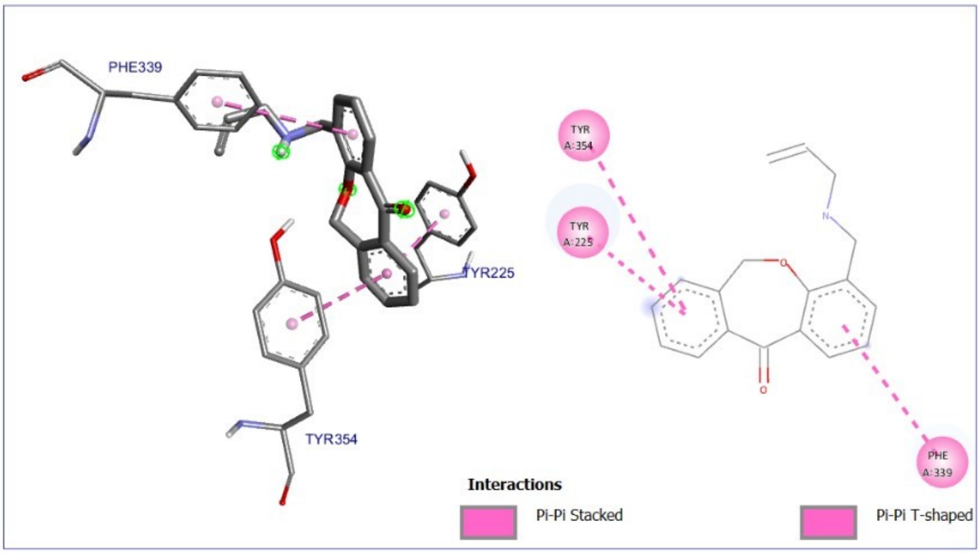

Figure S16. 3D and 2D interaction diagram of compound **7h** with antifungal protein

7i

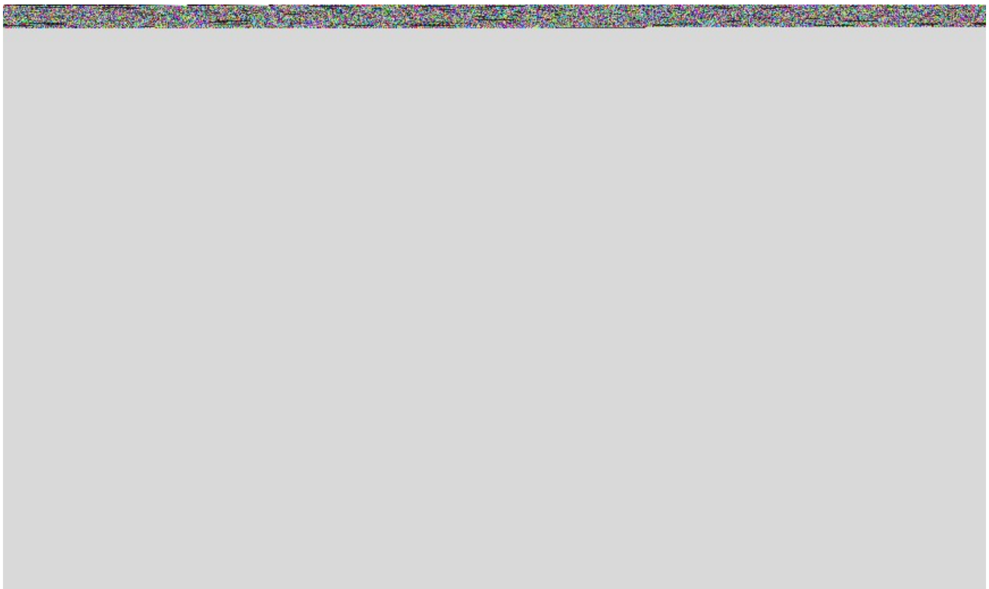

Figure S17. 3D and 2D interaction diagram of compound **7i** with antifungal protein.

7j

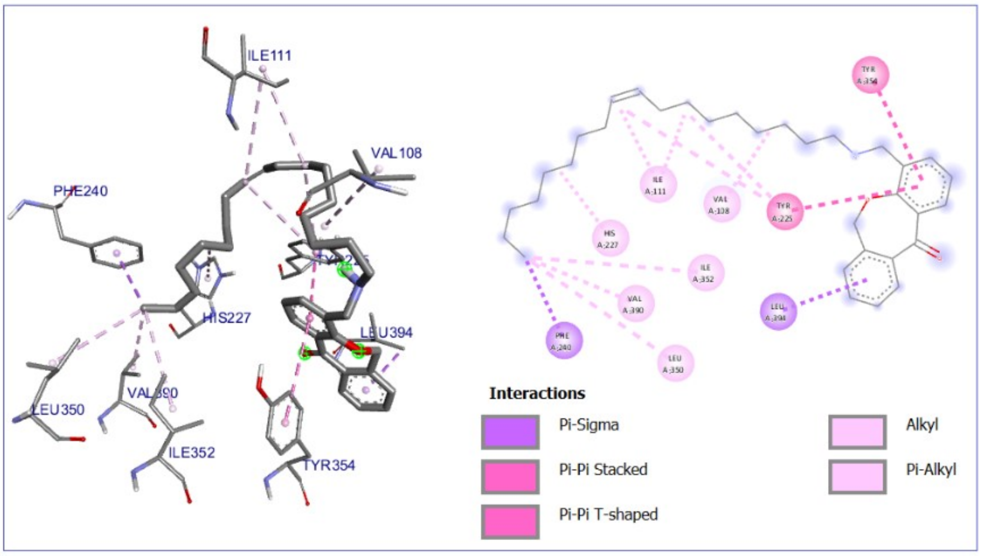

Figure S18. 3D and 2D interaction diagram of compound **7j** with antifungal protein.

6. Molecular Docking Images for ct-DNA activity (PDB ID : 1BNA).

7b

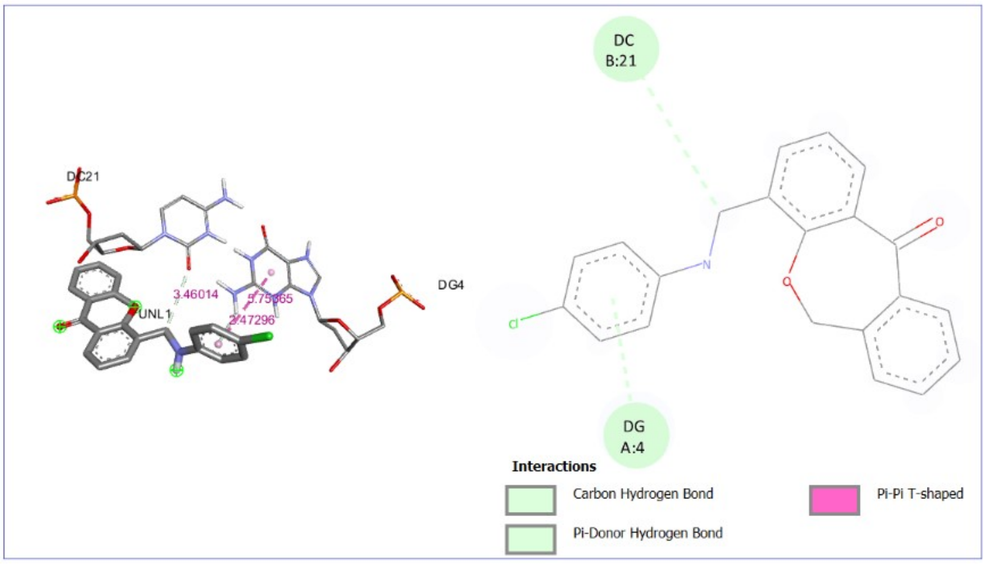

Figure S19. 3D and 2D interaction diagram of compound **7b** with ct-DNA .

7c

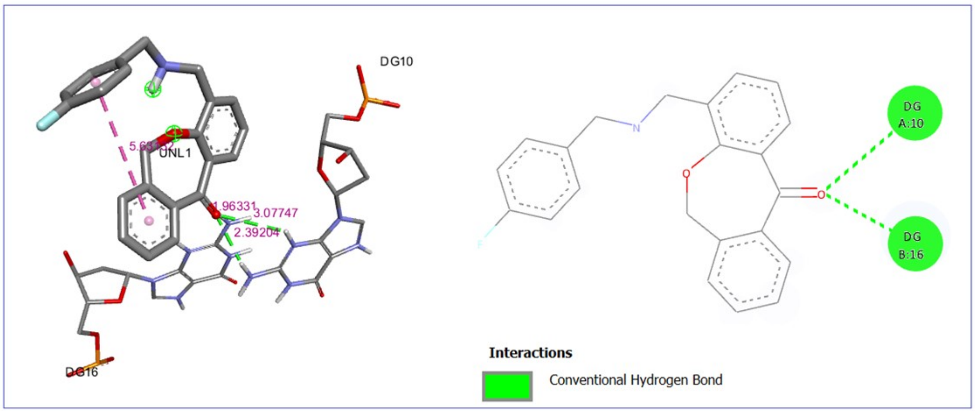

Figure S20. 3D and 2D interaction diagram of compound **7c** with ct-DNA.

7d

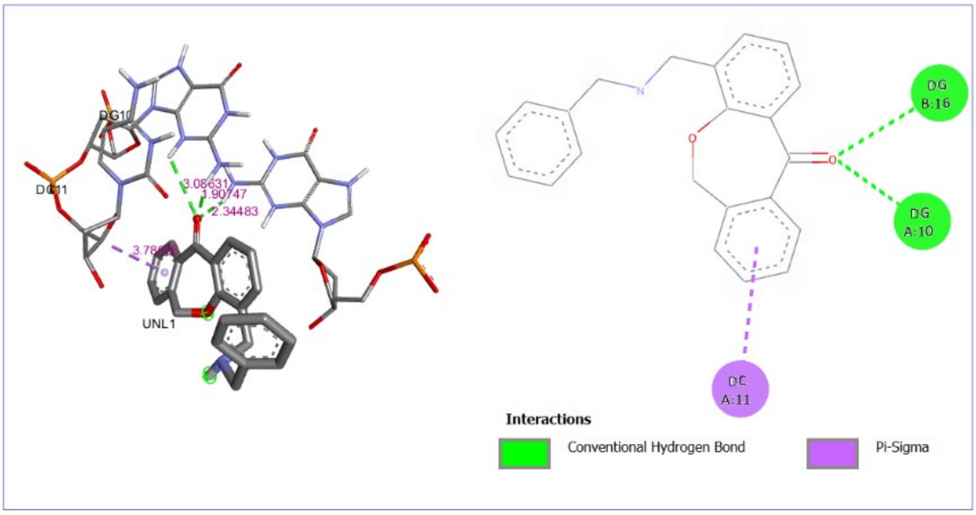

Figure S21. 3D and 2D interaction diagram of compound **7d** with ct-DNA.

7e

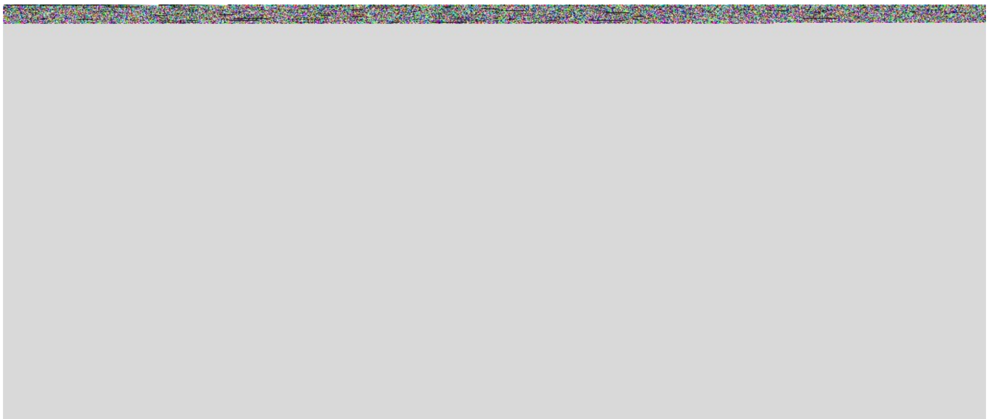

Figure S22. 3D and 2D interaction diagram of compound **7e** with ct-DNA.

7f

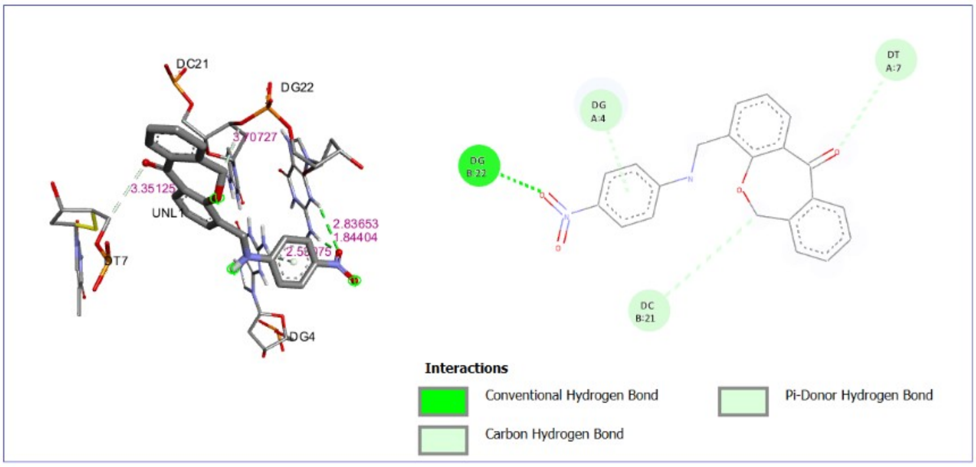

Figure S23. 3D and 2D interaction diagram of compound **7f** with ct-DNA.

7g

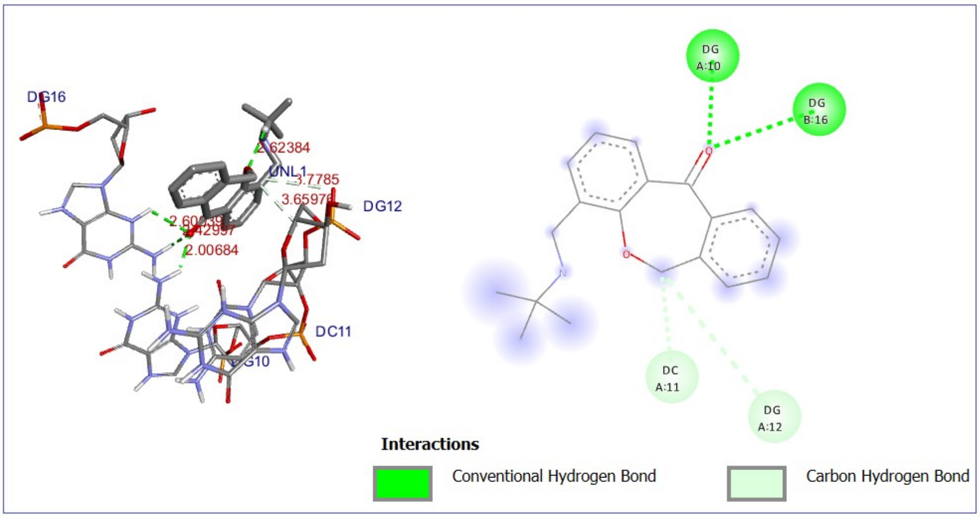

Figure S24. 3D and 2D interaction diagram of compound **7g** with ct-DNA.

7h

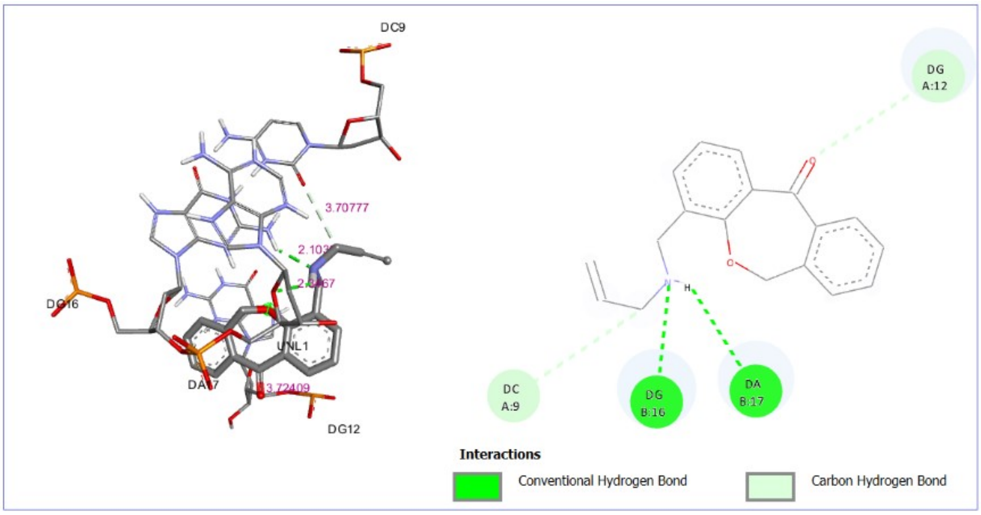

Figure S25. 3D and 2D interaction diagram of compound **7h** with ct-DNA.

7i

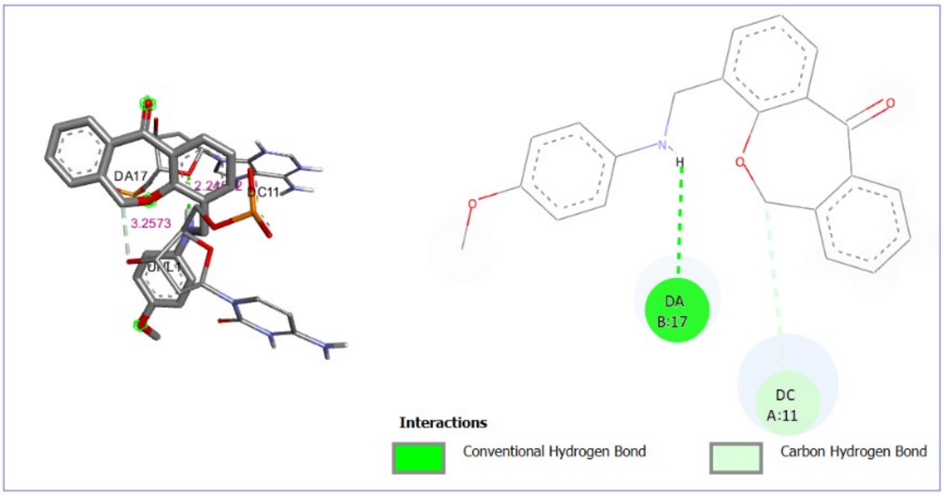

Figure S26. 3D and 2D interaction diagram of compound **7i** with ct-DNA.

7j

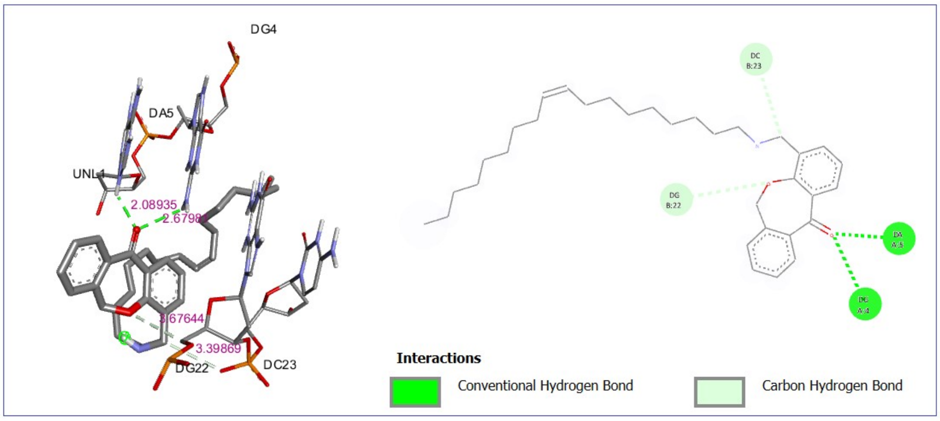

Figure S27. 3D and 2D interaction diagram of compound **7j** with ct-DNA.

8. Physicochemical Properties Values

Table S2. ADME and Physicochemical parameters

Ligands	LogP	TPSA	nOH	nOHNH	Nvio.	Nrot	BBB	Caco2	HIA	MDCK	SP	PPB
**7a**	4.84	38.33	3	1	0	3	2.06	52.04	96.73	1.34	-2.54	97.34
**7b**	5.07	38.33	3	1	1	3	2.34	42.67	96.92	0.96	-2.66	100
**7c**	4.26	38.33	3	1	0	4	0.504	51.66	96.56	0.096	-2.94	96.24
**7d**	4.09	38.33	3	1	0	4	0.37	53.35	96.56	0.77	-2.64	87.79
**7e**	5.20	38.33	3	1	1	3	2.59	42.67	97.03	0.03	-2.54	100

**7f**	4.35	38.33	6	1	0	4	0.02	8.98	97.03	0.18	-2.68	98.99
**7g**	3.88	38.33	3	1	0	3	0.53	48.31	95.94	3.66	-2.37	84.67
**7h**	3.34	38.33	3	1	0	4	0.86	47.51	95.99	26.63	-2.89	84.47
**7i**	4.45	38.33	4	1	0	4	0.39	49.63	96.34	0.86	-2.78	97.39
**7j**	9.31	38.33	3	1	1	18	16.48	52.18	97.05	65.00	-1.03	97.92

Table S3. Bioactivity Score Parameters

**Ligand**	**GPCR Ligand**	**Ion channel modulator**	**Kinase Inhibitor**	**Nuclear Receptor Ligand**	**Protease Inhibitor**	**Enzyme Inhibitor**
**7a**	0.08	-0.31	0.08	0.11	-0.09	0.06
**7b**	0.12	-0.25	0.10	0.12	-0.07	0.08
**7c**	0.14	-0.24	0.06	0.15	0.00	0.10
**7d**	0.14	-0.24	0.04	0.14	0.03	0.12
**7e**	0.02	-0.32	0.07	0.03	-0.14	0.04
**7f**	-0.02	-0.27	-0.04	0.04	-0.15	0.01
**7g**	0.19	-0.33	0.01	0.07	0.09	0.06
**7h**	0.11	-0.26	-0.12	0.00	-0.09	0.09
**7i**	0.08	-0.28	0.09	0.10	-0.06	0.07
**7j**	0.21	-0.14	0.00	0.13	0.10	0.16

9. DFT coordinates of UV-vis

1	6	0	-3.868148	0.044386	0.462946
2	6	0	-3.721170	-0.954560	-0.513745

3	6	0	-2.746574	-0.757355	-1.638711
4	6	0	-1.683788	1.446192	-0.027148
5	6	0	-0.911435	0.458312	-0.678388
6	1	0	-2.958009	0.166744	-2.193748
7	1	0	-2.787774	-1.596340	-2.337291
8	8	0	-1.391547	-0.720546	-1.176838
9	6	0	0.476268	0.629547	-0.881557
10	6	0	1.074854	1.815273	-0.476404
11	6	0	0.321445	2.833585	0.120708
12	6	0	-1.029568	2.643699	0.341158
13	6	0	-4.505831	-2.108320	-0.444249
14	6	0	-5.430299	-2.280422	0.585604
15	6	0	-5.581430	-1.286826	1.554594
16	6	0	-4.812261	-0.128875	1.485389
17	1	0	-4.394078	-2.874489	-1.206893
18	1	0	-6.030155	-3.184572	0.629951
19	1	0	-6.299389	-1.413049	2.359288
20	1	0	-4.926854	0.664501	2.215490
21	1	0	-1.631062	3.397881	0.834634
22	1	0	0.800573	3.758638	0.425180
23	1	0	2.139596	1.946250	-0.638216
24	6	0	1.251340	-0.483467	-1.585892
25	1	0	1.111637	-1.424407	-1.041841
26	1	0	0.797253	-0.659530	-2.567547
27	7	0	2.664804	-0.262857	-1.783665
28	1	0	2.922028	0.307948	-2.574188
29	6	0	3.632634	-0.387517	-0.790982
30	6	0	3.344418	-0.866236	0.498244
31	6	0	4.968999	-0.050415	-1.084455
32	6	0	4.363761	-1.013642	1.439470

33	1	0	2.325352	-1.107582	0.779042
34	6	0	5.967766	-0.197114	-0.130336
35	1	0	5.218314	0.323503	-2.075304
36	6	0	5.692870	-0.688216	1.154620
37	1	0	4.107277	-1.384255	2.429293
38	1	0	6.987822	0.075321	-0.392208
39	6	0	-3.103879	1.338150	0.458031
40	8	0	-3.637821	2.318535	0.970565
41	6	0	6.790934	-0.869738	2.175927
42	1	0	6.381506	-0.983637	3.184108
43	1	0	7.396576	-1.761190	1.967767
44	1	0	7.475266	-0.014307	2.189398

10. DFT coordinates of FTIR

1	6	0	-3.868148	0.044386	0.462946
2	6	0	-3.721170	-0.954560	-0.513745
3	6	0	-2.746574	-0.757355	-1.638711
4	6	0	-1.683788	1.446192	-0.027148
5	6	0	-0.911435	0.458312	-0.678388
6	1	0	-2.958009	0.166744	-2.193748
7	1	0	-2.787774	-1.596340	-2.337291
8	8	0	-1.391547	-0.720546	-1.176838
9	6	0	0.476268	0.629547	-0.881557
10	6	0	1.074854	1.815273	-0.476404
11	6	0	0.321445	2.833585	0.120708
12	6	0	-1.029568	2.643699	0.341158
13	6	0	-4.505831	-2.108320	-0.444249
14	6	0	-5.430299	-2.280422	0.585604
15	6	0	-5.581430	-1.286826	1.554594
16	6	0	-4.812261	-0.128875	1.485389
17	1	0	-4.394078	-2.874489	-1.206893
18	1	0	-6.030155	-3.184572	0.629951
19	1	0	-6.299389	-1.413049	2.359288

20	1	0	-4.926854	0.664501	2.215490
21	1	0	-1.631062	3.397881	0.834634
22	1	0	0.800573	3.758638	0.425180
23	1	0	2.139596	1.946250	-0.638216
24	6	0	1.251340	-0.483467	-1.585892
25	1	0	1.111637	-1.424407	-1.041841
26	1	0	0.797253	-0.659530	-2.567547
27	7	0	2.664804	-0.262857	-1.783665
28	1	0	2.922028	0.307948	-2.574188
29	6	0	3.632634	-0.387517	-0.790982
30	6	0	3.344418	-0.866236	0.498244
31	6	0	4.968999	-0.050415	-1.084455
32	6	0	4.363761	-1.013642	1.439470
33	1	0	2.325352	-1.107582	0.779042
34	6	0	5.967766	-0.197114	-0.130336
35	1	0	5.218314	0.323503	-2.075304
36	6	0	5.692870	-0.688216	1.154620
37	1	0	4.107277	-1.384255	2.429293
38	1	0	6.987822	0.075321	-0.392208
39	6	0	-3.103879	1.338150	0.458031
40	8	0	-3.637821	2.318535	0.970565
41	6	0	6.790934	-0.869738	2.175927
42	1	0	6.381506	-0.983637	3.184108
43	1	0	7.396576	-1.761190	1.967767
44	1	0	7.475266	-0.014307	2.189398

11. DFT coordinates of ESP

1	6	0	-3.451329	0.434352	0.392740
2	6	0	-3.363411	-0.976164	0.200582
3	6	0	-2.125794	-1.753149	-0.307919
4	6	0	-1.010988	1.173078	-0.627811
5	6	0	-0.426268	-0.094398	-0.614999
6	1	0	-2.112775	-1.781649	-1.377460

7	1	0	-2.190740	-2.748873	0.078366
8	8	0	-0.926811	-1.168970	0.172374
9	6	0	0.723181	-0.336128	-1.383522
10	6	0	1.355411	0.711065	-2.061201
11	6	0	0.841161	2.005757	-1.969023
12	6	0	-0.338745	2.235182	-1.253489
13	6	0	-4.482600	-1.772853	0.499289
14	6	0	-5.644325	-1.215328	1.037835
15	6	0	-5.706322	0.152835	1.286329
16	6	0	-4.619338	0.968724	0.964587
17	1	0	-4.441810	-2.826148	0.315424
18	1	0	-6.484407	-1.839212	1.261290
19	1	0	-6.586355	0.579242	1.720633
20	1	0	-4.678531	2.020503	1.152091
21	1	0	-0.737160	3.226246	-1.190582
22	1	0	1.345293	2.819007	-2.447955
23	1	0	2.232584	0.521688	-2.643956
24	6	0	1.299303	-1.761644	-1.470644
25	1	0	1.135459	-2.157244	-0.794880
26	1	0	0.858543	-2.274405	-2.299924
27	7	0	2.755828	-1.691000	-1.656227
28	1	0	2.709607	-1.181239	-2.515301
29	6	0	3.361357	-0.986548	-0.516936
30	6	0	3.775818	-1.706226	0.611872
31	6	0	3.524166	0.404708	-0.559621
32	6	0	4.353088	-1.034648	1.697995
33	1	0	3.651509	-2.768481	0.644463
34	6	0	4.101432	1.076286	0.526505
35	1	0	3.207716	0.954198	-1.421491
36	6	0	4.515888	0.356609	1.655315

37	1	0	4.669538	-1.584138	2.559866
38	1	0	4.225737	2.138542	0.493916
39	6	0	-2.377956	1.482156	0.002371
40	8	0	-2.661984	2.693063	0.193694
41	6	0	5.150246	1.094607	2.848859
42	1	0	5.844377	0.445996	3.341186
43	1	0	5.663227	1.965563	2.497879
44	1	0	4.383890	1.385029	3.53679

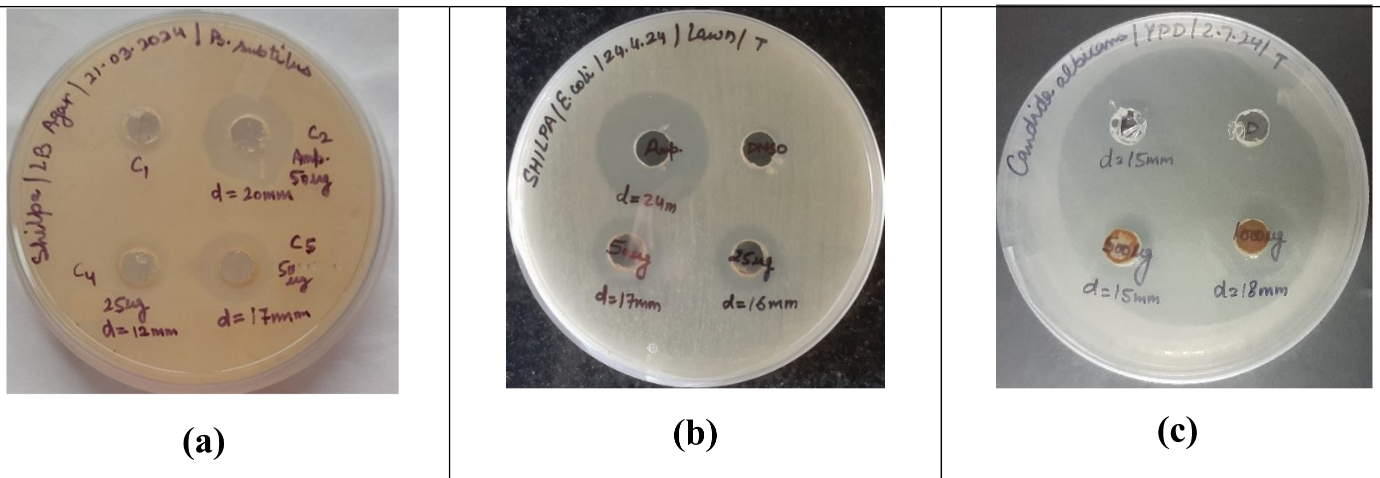

(a) (b) (c)

Figure S28. Antimicrobial activity of compound 7a against (a) Gram-positive *B. subtilis*: Pure DMSO (C1), Positive Control (C2), Conc. of 7a (C4) 25 µg, (C5) 50 µg, loaded onto 7-mm wells on agar plates, incubated at 37 °C for 24 h. (b) Gram-negative *E. coli*: concentrations of 7a as 25 µg and 50 µg, Positive control (Amp) loaded onto 7-mm wells on agar plates, incubated overnight at 37 °C. (c) *C. albicans*: concentrations 25 µg and 50 µg of 7a, positive control (Fluconazole) loaded onto 7-mm wells on agar plates, incubated at 30 °C for 16-20 h.

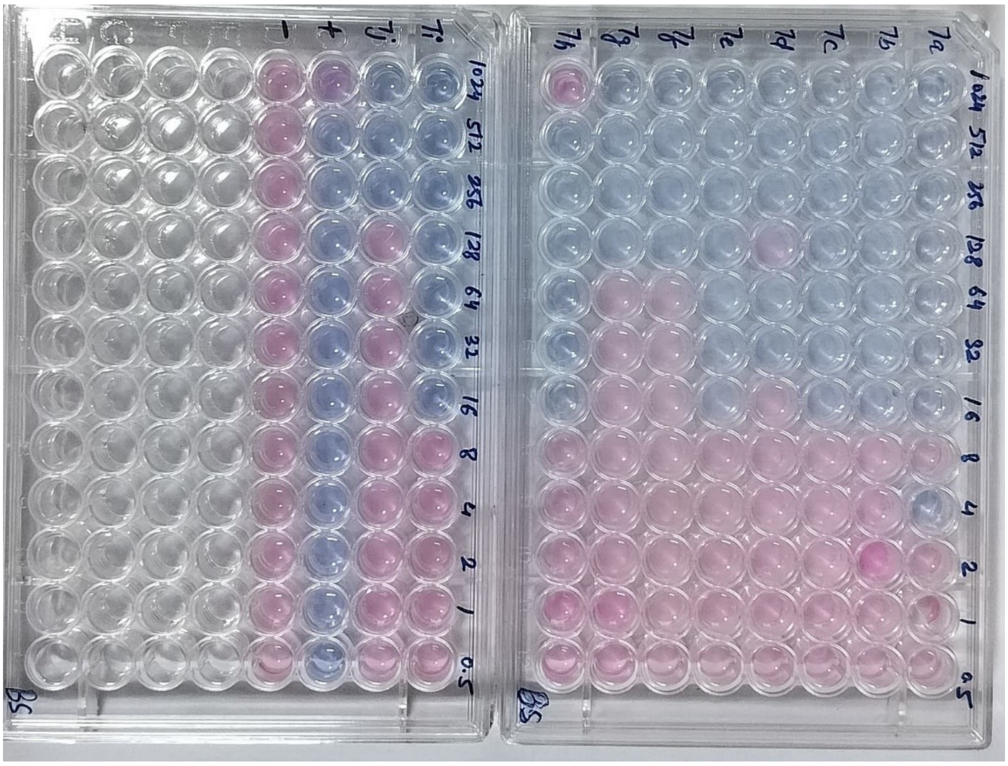

(a) *B. subtilis* gram-positive antibacterial strain

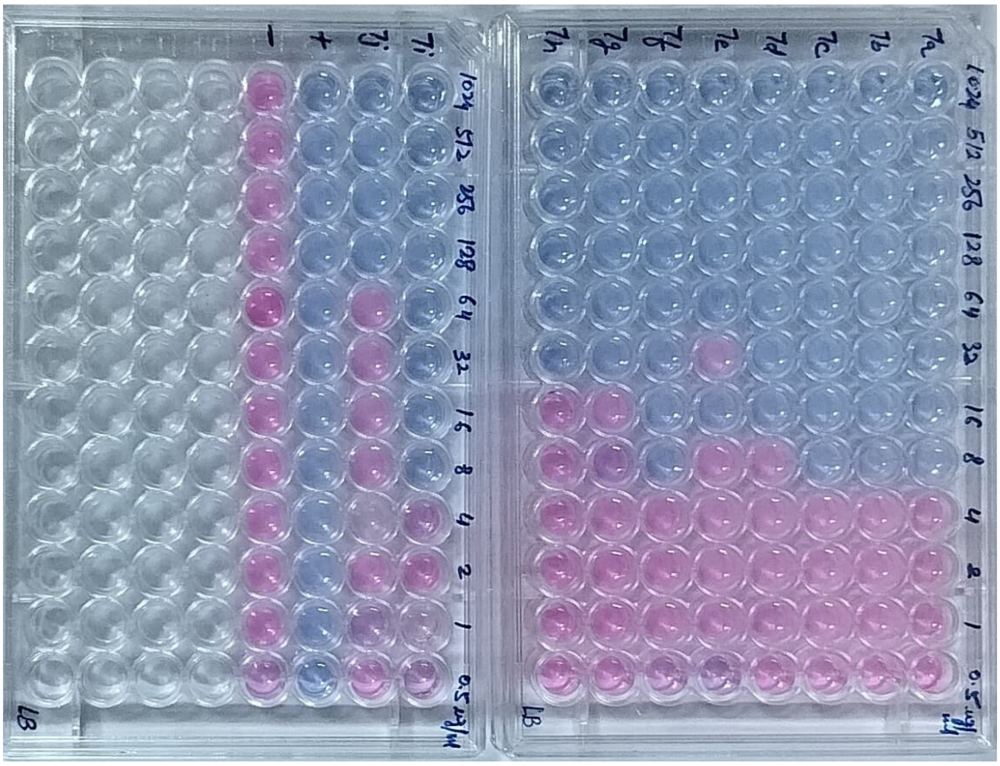

(b) *Lb. rhamnosus* gram-positive antibacterial strain

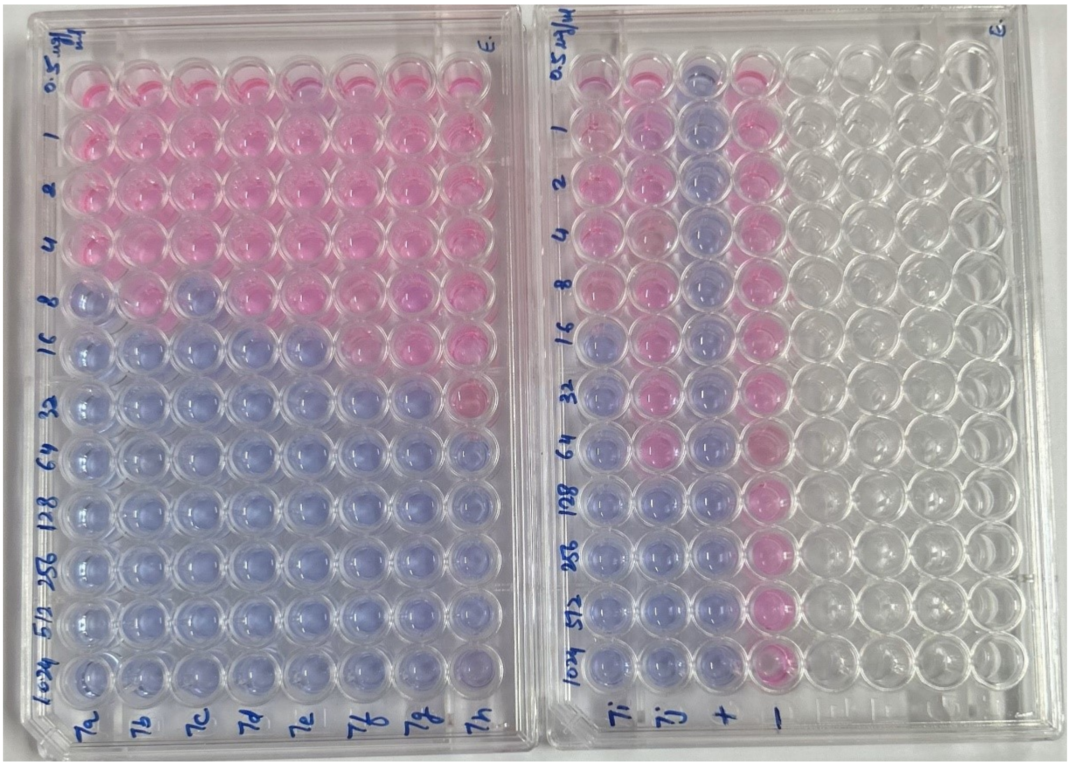

(c) *E.coli* gram-negative antibacterial strain

Figure S28. Minimum Inhibitory Concentration (MIC) values of compounds 7(a-j) against tested bacterial strains **a**(*LB. rhamnosus*), **b**(*B. subtilis*), **c**(E. coli). MIC values were determined using the broth microdilution method, with results expressed in µg/mL. Lower MIC values indicate higher antibacterial potency.
